# Towards pi-extended cycloparaphenylenes as seeds for CNT growth: investigating strain relieving ring-openings and rearrangements†
†Electronic supplementary information (ESI) available: Experimental procedures, spectroscopic data, crystal structure data, and coordinates and energies of DFT calculated stationary points. CCDC 1404657. For ESI and crystallographic data in CIF or other electronic format see DOI: 10.1039/c5sc04218f


**DOI:** 10.1039/c5sc04218f

**Published:** 2016-02-18

**Authors:** Thomas J. Sisto, Lev N. Zakharov, Brittany M. White, Ramesh Jasti

**Affiliations:** a Department of Chemistry & Biochemistry and Materials Science Institute , University of Oregon , Eugene , Oregon 97403-1253 , USA . Email: rjasti@uoregon.edu; b CAMCOR , University of Oregon , Eugene , Oregon 97403-1253 , USA

## Abstract

Despite significant multidisciplinary effort over many years, the preparation of uniform carbon nanotubes (CNTs) is still an unsolved problem in the scientific community. This inaccessibility hampers the commercial use of CNTs in electronic devices due to the sensitive connection between their electronic properties and molecular structure. The [*n*]cycloparaphenylenes ([*n*]CPPs), the smallest horizontal segment of an armchair CNT, hold great promise as “seeds”, or templates, for the preparation of homogenous batches of CNTs. Initial reports towards this goal, however, suggest that it would be advantageous to pi-extend these structures through traditional organic synthesis before their use in CNT growth. Towards this, several strategies have been reported attempting to utilize the Scholl reaction on aryl-substituted cycloparaphenylenes to yield a small CNT for use as a template for larger tubes. Prominently used in polyaromatic hydrocarbon chemistry, the Scholl reaction has afforded numerous extraordinary targets, such as graphene nanoribbons and graphene propellors. In this work, both experimental and computational studies are provided to unravel the complex cationic rearrangements and ring-openings associated with the Scholl reaction in the context of the cycloparaphenylenes—systems that are thermodynamically and kinetically different from flat graphene fragments. Additionally, this work demonstrates the unique reactivity of cycloparaphenylenes in the context of cationic or radical cationic intermediates, which are common reaction pathways for numerous transformations.

## Abstract 2

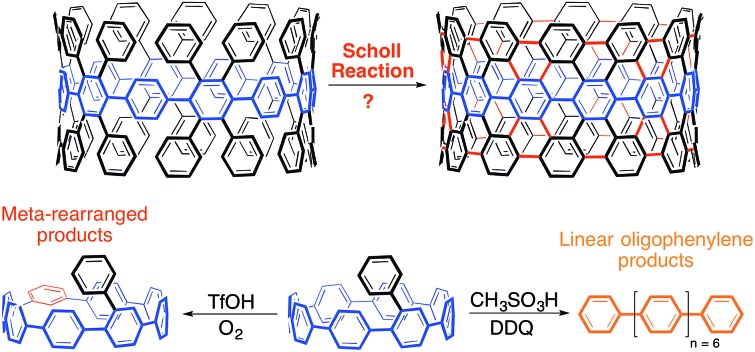
Upon exposure to Scholl reaction conditions, cycloparaphenylenes undergo facile strain-relieving rearrangements and ring-openings.

## Introduction

**Fig. 1:**
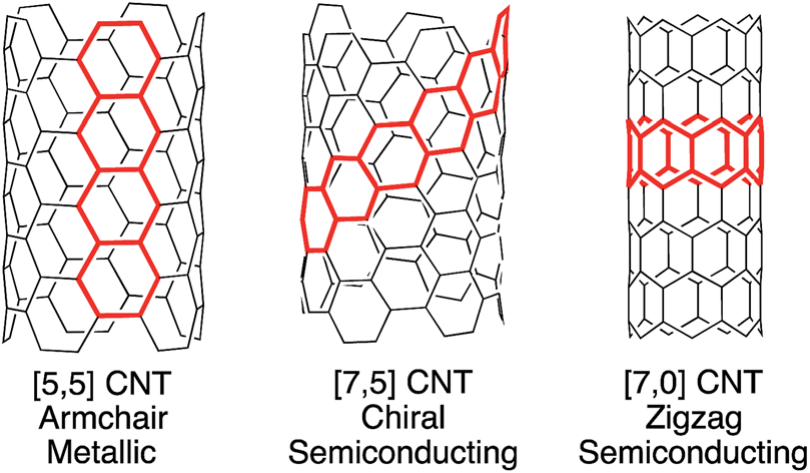
Carbon nanotubes are classified as armchair, chiral, or zigzag based upon their structure. Each type has differing electronic properties.

Carbon nanotubes (CNTs) are among the most promising materials for next generation technology, displaying extraordinary physical and electronic properties.^1^–^6^ Of these, the optoelectronic properties of CNTs are highly dependent upon molecular structure and tube diameter (Fig. 1). For example, certain CNTs are metallic, with conductivity that is 10^3^ times higher than copper, while others are semiconducting, with varying band gaps based upon chiral pitch and diameter.^7^ This relationship between molecular structure (or CNT “chirality”) and properties is exciting in that carbon nanotubes are inherently tunable. However, their commercial use in electronics is currently impractical, since there is no synthetic method or scalable purification technique available to provide uniform nanotubes despite years of multidisciplinary research.

**Fig. 2:**
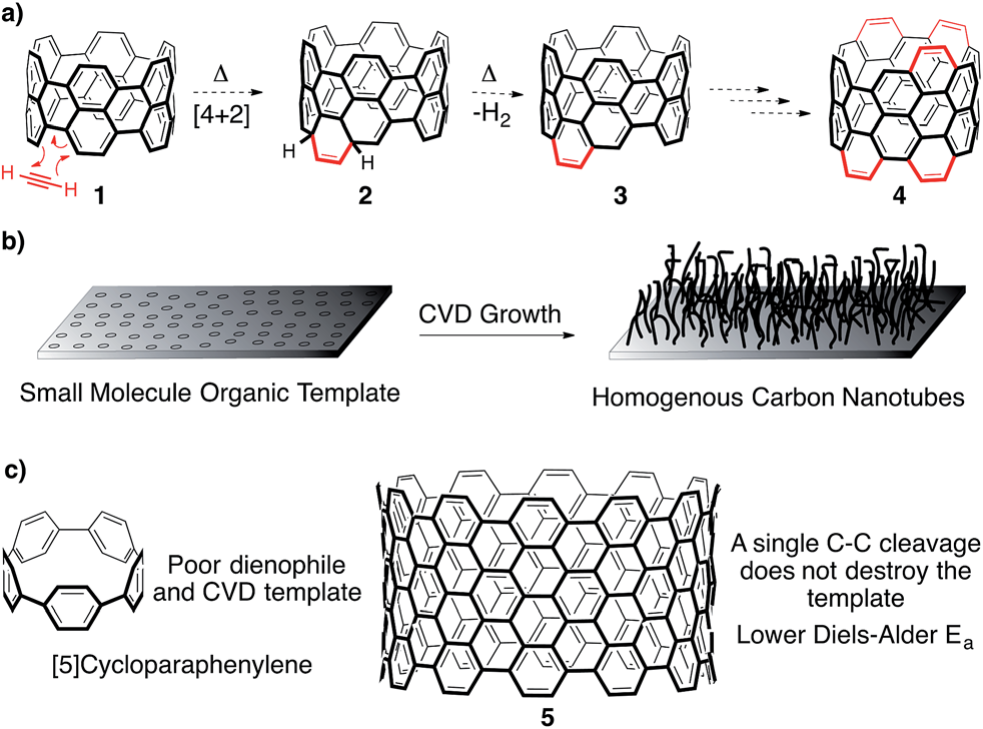
(a) A Diels–Alder polymerization approach to grow nanotubes from a CPP template. (b) Small molecule templates may provide homogenous carbon nanotubes under CVD growth conditions (c) [5]CPP and ultra-short CNT **5**, along with their key differences pertaining to their use as seeds for CNT growth.

One promising approach towards addressing this issue is the utilization of small molecule template to seed the growth of CNTs.^8^–^21^ In a general sense, extending a small CNT fragment of a predefined structure and diameter would provide uniform CNTs, provided no new nanotubes are formed under the growth conditions. Specifically, two methods of growth from a small molecule template have garnered significant attention in the literature. The first is based on a Diels–Alder polymerization strategy,^10^–^15^ while the second is an adaptation of known CNT production methods (Fig. 2a and b).^8^,^9^,^16^,^17^ Importantly, this approach of using a small molecule template to control CNT structure holds the promise of commercial viability. Although the molecular templates may be cost intensive due to multistep synthesis, one milligram of a seed molecule extended to 1 mm length nanotubes would yield over 4 kilograms of homogenous carbon nanotubes.^22^

The [*n*]cycloparaphenylenes ([*n*]CPPs), macrocycles consisting of *n* para-linked benzene rings, are the smallest horizontal segment of an armchair CNT (Fig. 2c).^23^–^28^ Since first reported in 2008 by Jasti and Bertozzi,^20^ CPPs with diameters of [5]-[18] have been accessed by the Jasti,^29^–^31^ Yamago,^32^,^33^ and Itami^34^ laboratories. Notably, certain diameter CPPs have also been prepared on large scale.^35^,^36^ In addition to their interesting photophysical,^37^ electronic,^37^ and host–guest properties,^35^,^38^–^40^ this class of molecules has been met with great excitement as potential templates for the growth of homogenous armchair CNTs.^8^,^12^,^16^ Though the CPPs are exciting as prospects for numerous applications, we hypothesize that a pi-extended CPP, such as **5** (Fig. 2c), is crucial for their success as templates in either a Diels–Alder or CVD growth strategy towards uniform CNTs (*vide infra*).

In regards to Diels–Alder growth of a cycloparaphenylene template (Fig. 2a), the reaction sequence begins with a [4 + 2] cycloaddition between the “bay region” of the template (**1**) and acetylene, or an acetylene equivalent.^10^–^15^ Though this step temporarily breaks the aromaticity of the template structure (**2**), it will rearomatize spontaneously under the reaction conditions through a thermal extrusion of H_2_ (**3**). This process could then continue *ad infinitum* with excess acetylene, since new “bay regions” are created as the reaction proceeds (**4**). The most attractive feature of the Diels–Alder extension strategy is that the reaction conditions will clearly not form new nanotubes, but rather simply extend the seeds provided. In both our and other's hands, however, no cycloparaphenylenes have exhibited Diels–Alder reactivity.^41^ This observation is not surprising since there is no known Diels–Alder reaction of biphenyl or terphenyl, which the reactivity of the CPPs appears to mirror. However, pi-extended cyclic fragments (such as **5**) are expected to participate in this reaction based on calculations reported by the Scott group.^12^ Furthermore, Scott and coworkers have shown experimentally that larger linear PAH fragments, such as bisanthene, readily undergo Diels–Alder benzannulations.^10^,^11^

Alternatively, another mode for extending small molecule templates into homogenous batches of nanotubes is by known chemical vapor deposition growth methods (Fig. 2b).^8^,^9^,^16^,^17^ This has been shown to be possible through groundbreaking studies reported by Smalley and coworkers,^9^ along with collaborative reports by Fasel and Amsharov.^17^ Unfortunately, the methods are impractical for large scale production. Recently, Itami reported CNT growth from CVD conditions that do not create CNTs without CPPs present.^16^ Positing the homolytic scission of an aryl C–H bond of the CPP at high heat followed by radical addition of a carbon source as the mechanism of growth, CNTs are generated from [9]- and [12]CPP. Preliminary data suggests that the CPP template influences the resultant CNT diameters. Unfortunately, the low yields of the process limited the physical characterization to Raman spectroscopy, which showed a distribution of CNT diameters along with various sidewall chiralities. If the mechanism proposed is correct, the almost identical nature of the homolytic bond strength of an aryl C–H bond (113 kcal mol^–1^) and aryl C–C bond (114 kcal mol^–1^) would be expected to lead to undesirable C–C bond fragmentation, and thus low yields, at the high temperatures required. Only one C–C bond cleavage is required to ring-open the CPP to a linear oligophenylene, destroying the template (Fig. 2c). In contrast, a single C–C bond cleavage for a template such as **5** would likely lead to recombination before any ring-opening process could occur (Fig. 2c). Therefore, although encouraged by this initial report from Itami, we have hypothesized that a pi-extended CPP is crucial for high yielding CVD growth.

**Fig. 3:**
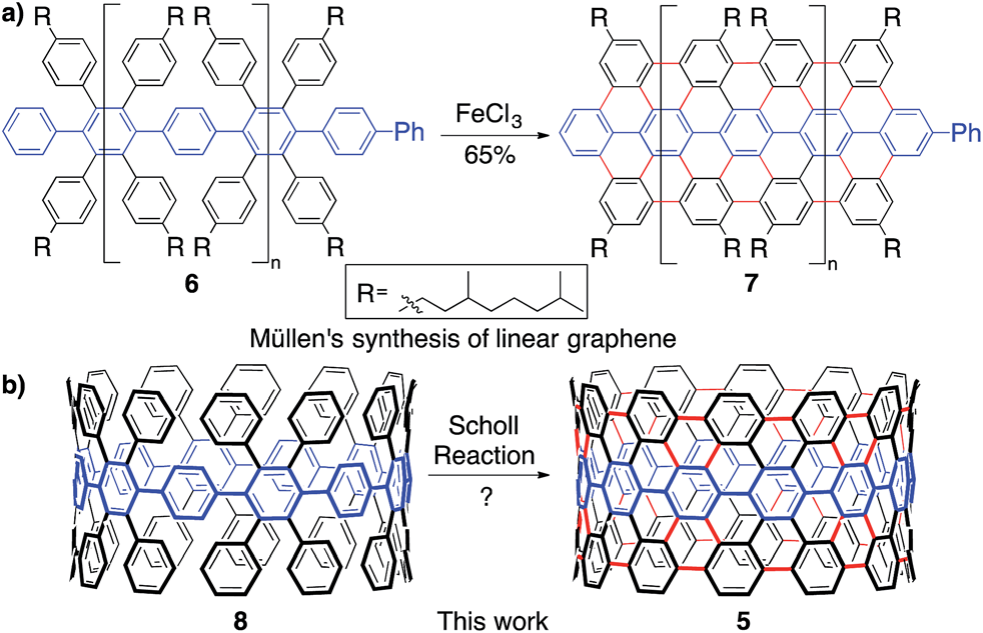
(a) The successful utilization of a Scholl reaction to synthesize linear graphene nanoribbons (**7**) reported by Müllen. (b) Aryl-substituted [12]CPP **8** that is suggested to undergo a Scholl reaction to CNT **5**.

**Fig. 4:**
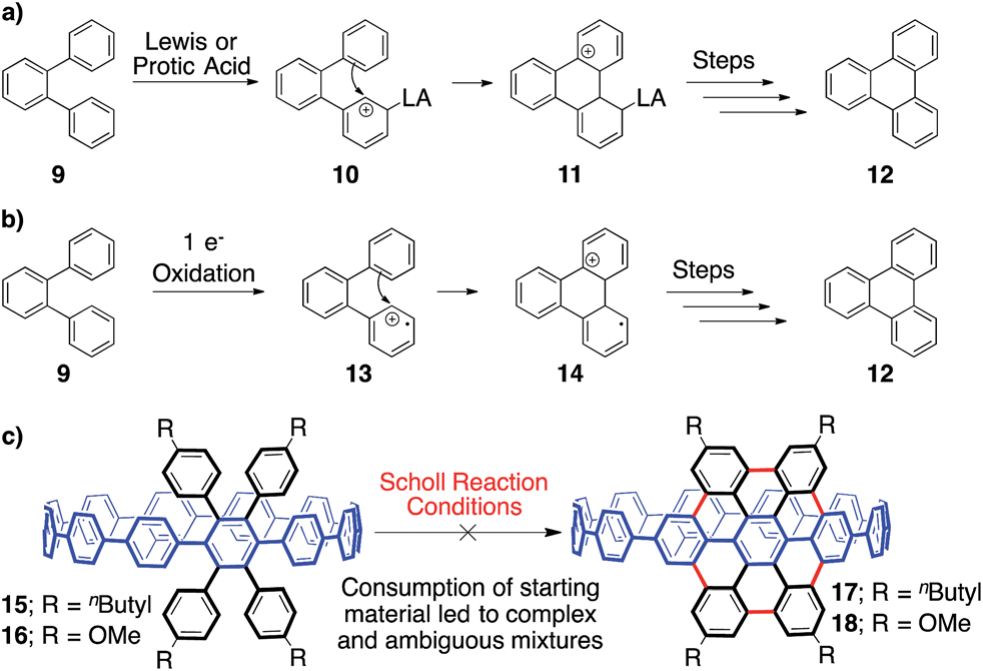
(a) The cationic Scholl mechanism. (b) The radical cationic Scholl mechanism. (c) Investigation of tetraphenyl-substituted [12]CPPs **15** and **16** as model systems for a Scholl reaction towards **5**.

To synthesize an elongated CPP such as **5** (*i.e.* an ultrashort CNT), the most straightforward retrosynthetic disconnection is a Scholl reaction.^42^,^43^ The Scholl reaction, an oxidative biaryl coupling, is attractive since it circumvents the need for complex pre-functionalization patterns. Additionally, this reaction is commonly used in the synthesis of polyaromatic hydrocarbons, including the extraordinary graphene propellors and nanoribbons reported by Müllen and coworkers (**6** to **7**; Fig. 3a).^44^,^45^ Drawing inspiration from these structures, we were attracted to a target such as highly aryl-substituted [12]CPP (**8**) to serve as a platform for the synthesis of ultrashort CNT **5** (Fig. 3b). Towards **8**, we recently reported the synthesis of tetraphenyl-substituted [12]CPP **15**, which also served as a model system to develop methodology to transform CPP **8** into CNT **5** (Fig. 4c).^21^ Simultaneous to us, the Müllen group has also attempted to utilize Scholl chemistry towards the synthesis of targets like CNT **5**, with limited success.^18^,^19^ We herein provide a combined experimental and computational study of the reactivity of the cycloparaphenylenes under a broad cationic scope, indicating the propensity of these kinetically-trapped cyclic structures to undergo strain-relieving rearrangements and ring-openings.

## Results and discussion

### Synthesis and characterization

In approaching a complex synthetic target such as CNT **5**, we envisioned using tetraphenyl-substituted [12]CPP **15** as a model system to probe and optimize a Scholl reaction on a nonplanar, strained system—one quite different than the flat PAHs that populate the Scholl literature (Fig. 3a, 4a and b). The synthesis of compound **15** was reported previously using an oxidative dearomatization/reductive aromatization strategy developed in our group (Fig. 4c).^21^

With model system **3** in hand, we turned our attention towards elucidating the optimal conditions for transforming **15** to **17**. Previous studies of the Scholl reaction clearly demonstrate that success is highly dependent upon reaction conditions.^42^,^46^–^49^ Furthermore, the mechanism, either cationic or radical cationic, is often debated, and is likely substrate and reaction condition dependent (Fig. 4a and b). The cationic pathway begins with a protonation, or coordination, to form a carbocation (**10**), followed by an intramolecular Friedel–Crafts reaction (**11**). Following these steps, dehydrogenation provides the rearomatized product (**12**). The radical pathway, beginning with a one electron oxidation rather than a protonation/coordination (**13**), is similar but mechanistically distinct. Our experimental investigations attempted to probe both cationic and radical cationic based conditions.

Unfortunately, upon extensive reaction screening with **15**, it was apparent that a variety of Scholl conditions were all providing extremely complex and inseparable reaction mixtures. Increasing the electronic density of the appended aryl rings to lower the oxidation potential and increase nucleophilicity (**16**, Fig. 4c) had no effect on the complexity of the results. Although pared down from elaborate substrate **8**, these model systems were simply not informative. Due to the difficulty of separating the complex mixture formed, we were never able to isolate and clearly characterize anything from the reaction. When confronted with this mixture of products, ^1^H NMR spectroscopy was uninformative due to the similarity of the possible products, which contain anywhere from 60–72 overlapping signals from aryl protons. Though crude MALDI-TOF data did not show the desired product, we could not rule out that it might be formed in low yield.

**Fig. 5:**
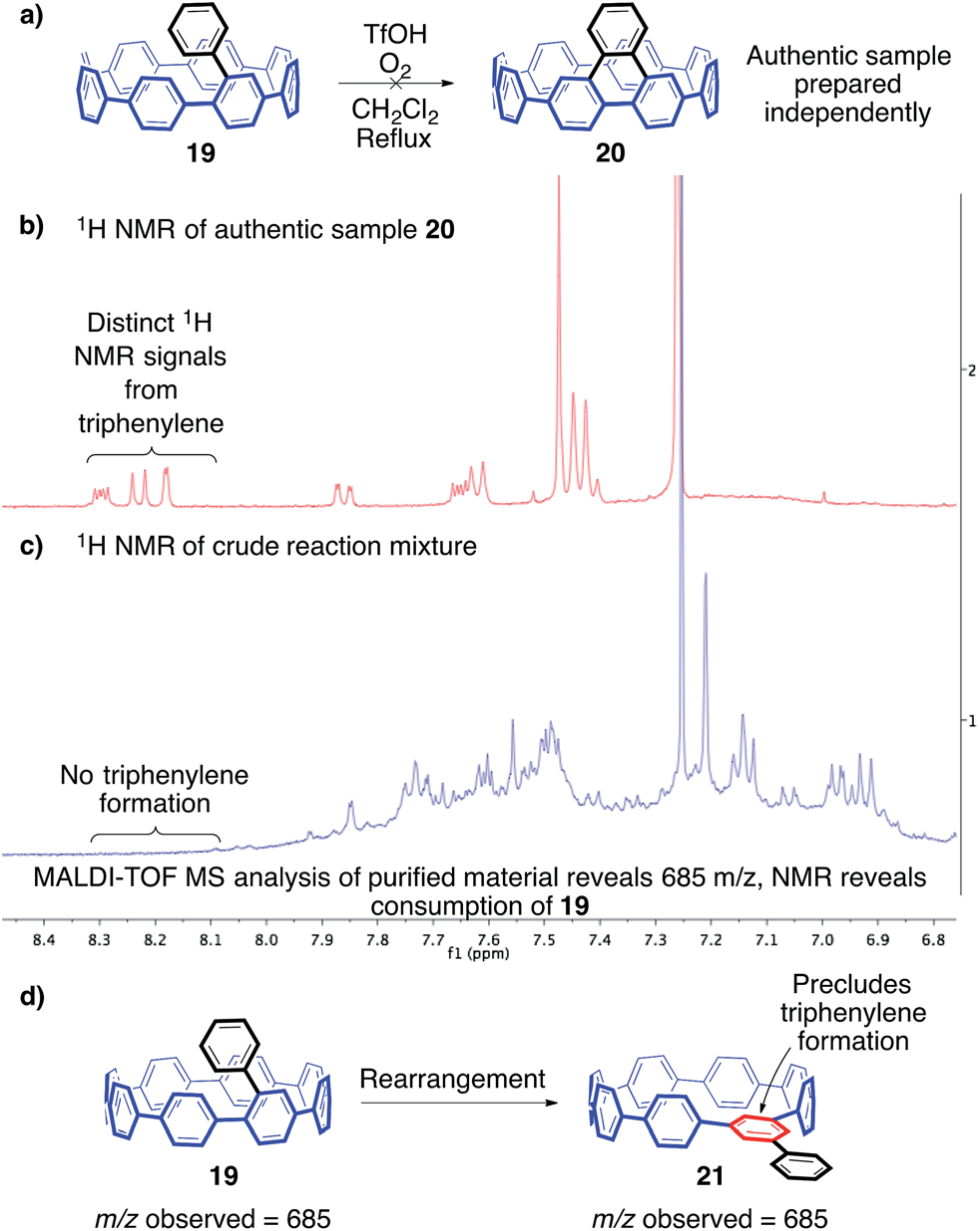
(a) Representative cationic Scholl reaction for the attempted transformation of **19** to **20**. (b) ^1^H NMR of authentic sample **20**. (c) ^1^H NMR of the crude mixture produced from the reaction shown in (a). (d) An isomer such as **21** geometrically precludes triphenylene formation.

In an attempt to better understand the Scholl reaction in the context of the cycloparaphenylenes, we targeted a further simplified model compound, monoaryl-substituted [8]CPP **19** (Fig. 5a). In this case, only one Scholl reaction is necessary to deliver the target compound. Additionally, we surmised that this particular bond-forming step is likely the most difficult, since it is intrinsically tied to the strain of the CPP (Fig. 5a). To avoid an inconclusive outcome similar to our previous studies, we aimed to definitively prove whether any desired product was formed. Since the triphenylene subunit in the desired product is a known PAH with characteristic ^1^H NMR resonances from 8–9 ppm, we anticipated that these unique signals would be easily identifiable by ^1^H NMR of the crude material, even in a low yielding reaction. However, previous experiences with the unique conformations of the CPPs and the subsequent impact on ^1^H NMR resonances led us to be cautious about this assumption. To address this, we synthesized [8]triphenylene-CPP (**20**, Fig. 5a), the product of a successful Scholl reaction of **19**, through a separate synthetic route (ESI, Fig. S3†). The synthesis of authentic sample **20** gratifyingly confirmed that the ^1^H NMR signals of the triphenylene are in fact distinct (Fig. 5b), while also providing us with a standard for additional analytical methods.^50^

With our simplified platform (**19**) and authentic sample (**20**) prepared, we first turned towards probing cationic methods of inducing a Scholl reaction. After numerous reaction screens, the most informative conditions were those reported by Johnson and co-workers utilizing triflic acid and molecular oxygen at 40 °C in dichloromethane.^51^ These conditions avoid the complication of halogenation often observed when utilizing common Scholl reagents such as FeCl_3_. Avoiding these halogenation side reactions is critical due to the potential for multiple chlorinations, which would lead to a complex mixture of regioisomers. Upon exposure to these reaction conditions, **19** was consumed rapidly as evidenced by ^1^H NMR spectroscopy (Fig. 5c). Interestingly, no desired product, or any characteristic triphenylene signals, were observed.^52^ Silica gel chromatography yielded one band, which appeared to contain a complex mixture of products by ^1^H NMR spectroscopy. MALDI-TOF analysis of this mixture revealed a prominent peak corresponding to ions of the same *m*/*z* as the starting material. Clearly, exposure of **19** to these reaction conditions resulted in the production of a complex mixture of compounds isomeric with the starting material. Additionally, since no triphenylene signals were observed, it is reasonable to conclude that these isomers geometrically precluded triphenylene formation (isomers such as **21**). Most importantly, these results indicate that these structural rearrangements are faster than C–C bond formation (Fig. 5d).

**Fig. 6:**
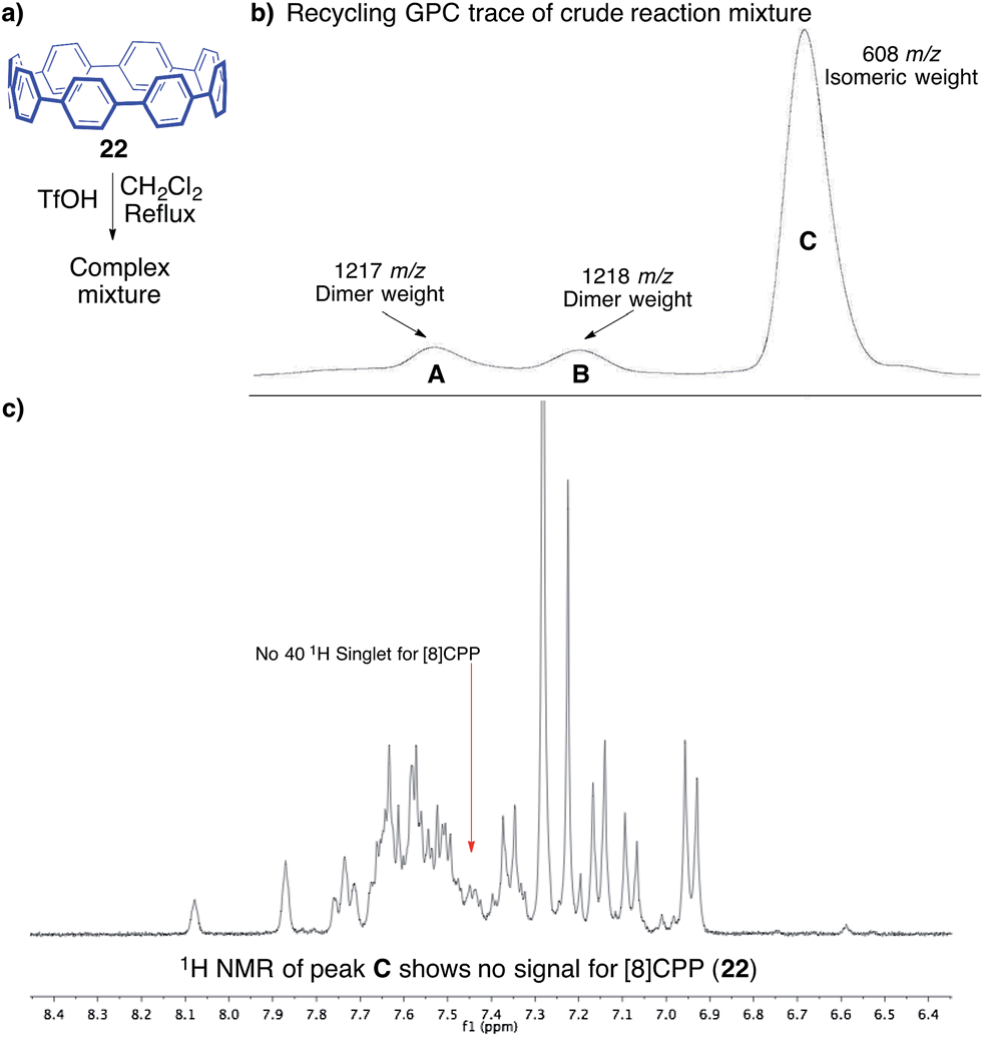
(a) Reaction to investigate the potential cationic rearrangement of [8]CPP. (b) Recycling GPC trace of the crude reaction mixture, along with the corresponding MALDI-TOF masses. (c) ^1^H NMR of fraction C collected from the recycling GPC.

To support our hypothesis of rapid structural rearrangements, we anticipated that exposure of [8]CPP to these reaction conditions would also lead to isomeric compounds. When subjecting [8]CPP (**22**) to triflic acid in dichloromethane at 40 °C, we again observed rapid consumption of the starting material (Fig. 6a).^53^^1^H NMR analysis of the crude reaction mixture, and the single band afforded through silica gel chromatography, displayed what appeared to be a complex mixture of isomers. Since silica gel chromatography did not provide sufficient separation, we turned to recycling GPC as a potential means of separating isomers of different hydrodynamic radius. Three fractions, one major (fraction C, Fig. 6b) and two minor (fractions A and B, Fig. 6b), were separable. Notably, each had different retention times than the starting material, [8]CPP. After four passes through the recycling column to ensure purity, each fraction was collected for further characterization. The major fraction (C) contained enough mass for characterization by ^1^H NMR, which indicated a mixture of compounds that did not include [8]CPP (Fig. 6c). MALDI-TOF MS of this fraction displayed a peak of 608.1 *m*/*z*, corresponding to isomers of [8]CPP.

The two minor fractions contained only enough mass for MALDI-TOF MS analysis. Interestingly, these two fractions each displayed a weight which is assignable as a dimer of [8]CPP (1217 and 1218 *m*/*z*). We attribute the presence of two separable, yet similar, fractions to multiple isomers of these dimers of [8]CPP. Dimerization, and isomerization of these dimers, is certainly a possible product under these cationic conditions, which adds yet another layer of complexity to the reaction profile. This assignment is also supported by the significantly larger hydrodynamic radius (shorter GPC retention time) as compared to [8]CPP.^54^

**Fig. 7:**
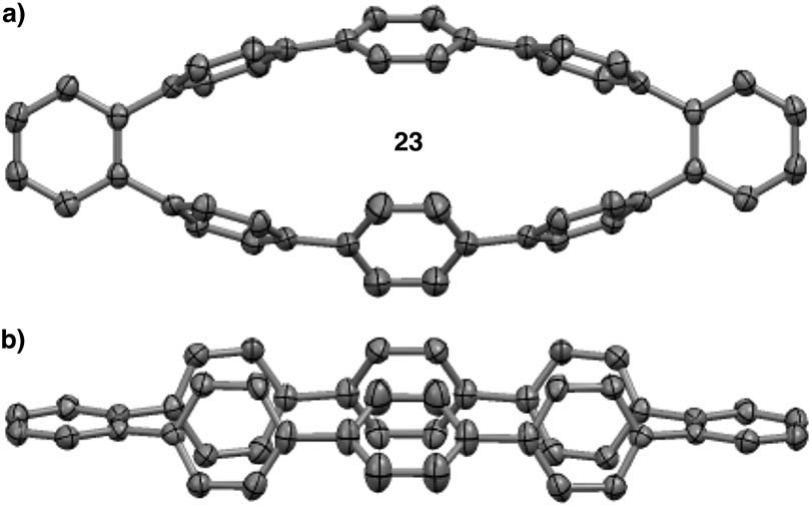
(a) Top down view of the crystal structure of isomer **23**. (b) Side view of the crystal structure of isomer **23**.

The MALDI-TOF analysis, the distinct retention times from [8]CPP (**22**), and the ^1^H NMR analysis showing no singlet for [8]CPP strongly supported the conclusion that several isomers were being formed. However, we still had not isolated a discrete compound to unequivocally prove this fact. Fortunately, slow evaporation of major GPC fraction C from a solution of dichloromethane and hexanes furnished a crystal suitable for X-ray diffraction (Fig. 7). The structure was solved to reveal **23**, a compound that is clearly a rearranged isomer of [8]CPP (**22**).^55^

To potentially avoid the rearrangements we had observed thus far, we next turned towards probing conditions known to induce radical cation mediated Scholl reactions.^49^ Exposing **19** to DDQ and methanesulfonic acid (conditions developed by Rathore and coworkers) led only to insoluble material. Unfortunately, this material was uncharacterizable due to complete insolubility in all organic solvents. Consistent with previous observations in our lab, we have tentatively assigned this material as a linear oligoparaphenylene (OPP) based on its complete insolubility and the change in fluorescence to orange (expected for [8]OPP). A control experiment utilizing unsubstituted [10]CPP also produced an insoluble material with orange fluorescence. Although acid is present in these conditions, we know from our previous studies that acid catalyzed rearrangements do not yield these insoluble products. Therefore we attribute this presumed ring-opening to a radical cation based mechanism.

### Investigation of proposed reaction pathways

**Fig. 8:**
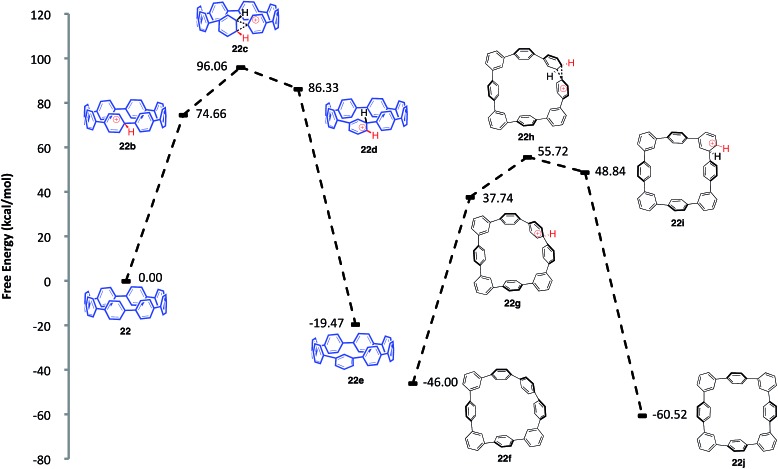
Calculated reaction coordinate for the ipso protonation and subsequent meta-rearrangement of [8]CPP (**22**).

Aware that cationic rearrangements were competitive processes to our desired Scoll reaction, we were still surprised to see no triphenylene formation of any kind, even within rearranged structures. This observation was critical to our understanding of the type of isomers being formed. Additionally, the complete lack of triphenylenes indicated that these rearrangements are occurring much faster than C–C bond formation. From these results, we propose a mechanism of rearrangement involving *ipso* protonation of the CPP backbone, followed by a 1,2-aryl shift (Fig. 8, discussed in detail in a subsequent paragraph). This proposed mechanism is consistent with the formation of isolated compound **23** (Fig. 7).

**Fig. 9:**
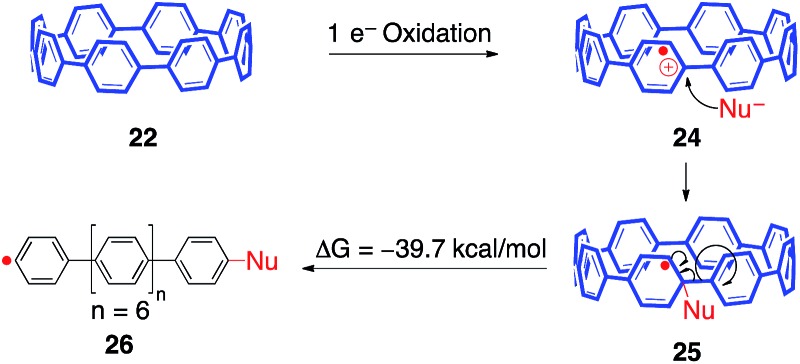
A proposed mechanism for a radical induced ring-opening of the CPPs.

Unfortunately, exposure of both mono-aryl substituted [8]CPP **19** and [10]CPP to oxidative radical Scholl conditions simply afforded insoluble solids. We have tentatively assigned these solids as ring-opened oligoparaphenylenes, which can be explained through a strain relieving homolytic scission of the *para* C–C bond of the CPP backbone (Fig. 9). Starting with a single electron oxidation of [8]CPP (**22**) to afford radical cation **24**, a nucleophilic addition could yield radical **25**. This intermediate may be favorable due to strain relieving pyramidalization. Intermediate **25** may then homolytically fragment to afford linear phenyl radical **26**, providing 39.7 kcal mol^–1^ of strain relief.^56^ This process is the reverse of the widely observed addition of phenyl radical to benzene to afford biphenyl. We postulate that this common reaction is driven in reverse due to strain relief.^57^

To probe the energy profile of our mechanistic proposal for cationic conditions, we turned to DFT calculations at the B3LYP/6-31+(d,p) and B3LYP/6-31(d,p) level of theory.^58^ All calculations were conducted in the gas phase due to the computational time associated with utilizing a solvation method on these large systems. The effect of solvation is expected to be negligible within each charged surface, and is only expected to affect the energies associated with transitioning from neutral to charged surfaces. We utilized Spartan 14 and Gaussian 09 to probe the energetics of protonation, a common first step of both the Scholl reaction and the proposed strain relieving aryl shifts, followed by subsequent rearrangements. All transition states were found to contain only one imaginary mode.^59^

We first turned to [8]CPP (**22**) to investigate the energetics associated with a cationic 1,2-aryl shift in the backbone of a CPP (Fig. 8). It is known that triflic acid will protonate aromatics such as terphenyl, so it was assumed that a CPP will also undergo facile protonation. Upon minimization of protonated [8]CPP (**22b**), a Δ*G* of 74.66 kcal mol^–1^ between **22** and **22b** makes this a reasonable assumption. This protonation energy only seems large due to the fact that the cation is unsolvated, and this energy is actually lower than the Δ*G* of 92.5 kcal mol^–1^ found by Johnson for the unsolvated protonation of terphenyl (which has a calculated Δ*G* of 16.7 kcal mol^–1^ upon PCM solvation).^49^ The activation energy for a 1,2-aryl shift (**22c**) was found to be 21.4 kcal mol^–1^, certainly an accessible energy under the reaction conditions. Upon deprotonation, the single meta-shifted macrocycle (**22e**) was found to be lower in energy by –19.47 kcal mol^–1^ as compared to the original [8]CPP (**22**). Noting that the ^1^H NMR obtained from the experimental conditions showed what appeared to be numerous isomers, we continued the calculations for the [8]CPP system onto a molecule that contained three arbitrarily chosen meta-shifted aryl rings (**22f**). This macrocycle (**22f**) with three shifts was found to lie –46.00 kcal mol^–1^ below the starting [8]CPP (**22**), clearly indicating that under thermodynamic conditions there is an extremely large driving force for [8]CPP to rearrange to 1,2-shifted isomers. Macrocycle **22f** was found to protonate and shift with approximately the same activation energy as that found for the shift from [8]CPP (**22**) to singly shifted **22e**. Since molecule **22f** begins with less strain than [8]CPP (**22**), it is not surprising that there is less strain relief (–14.52 kcal mol^–1^) from a 1,2 shift, though it is still quite large. One can imagine numerous other rearrangement patterns that might energetically lie near the isomers calculated, such as isolated and crystallized **23** (–53.1 kcal mol^–1^ as compared to [8]CPP).

**Fig. 10:**
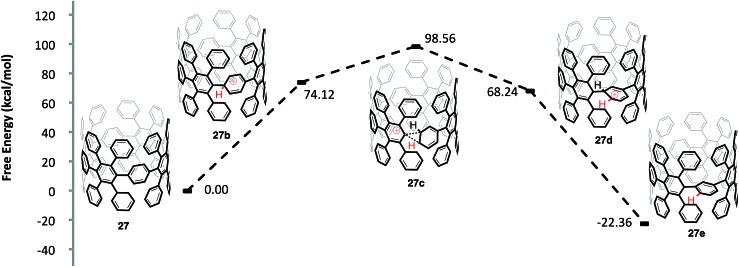
Calculated reaction coordinate for the *ipso* protonation and subsequent meta-rearrangement of aryl-substituted [8]CPP (**27**).

One question that is apparent throughout our investigation of these cationic rearrangements on [8]CPP (**22**) was whether our findings would translate to the more important highly substituted system **27** (Fig. 10). In regards to the viability of the proposed shifts, two major issues arise when comparing aryl-substituted [8]CPP **27***versus* [8]CPP (**22**): (1) the site of protonation and (2) the sterics of the transition state conformation. In regards to protonation, the HOMO of structure **27** is localized on the CPP backbone.^60^ This fact leads us to hypothesize that the strained, high energy pi-system of the CPP backbone will protonate preferentially, just as in the case of [8]CPP. A more difficult question regards the sterics of the transition state and whether the conformation necessary to access the rearrangement will be energetically accessible in the case of **27**. To investigate this question, we modeled the energetics of a 1,2-aryl shift of the precursor (**27**) to an [8,8]CNT similar to **5**. By modeling a smaller diameter system than **5**, we have accentuated the potential for sterics to prevent these 1,2-aryl shifts. The protonation of **27** to form **27b** was similar in energy to the protonation of [8]CPP (**22** to **22b**) with a Δ*G* 74.12 kcal mol^–1^. Interestingly, the Δ*G*^‡^ of 24.44 kcal mol^–1^ from the protonated species (**27b**) to the transition state for a 1,2-aryl shift (**27c**) is also similar to that calculated for the [8]CPP series (**22b** to **22c**). As is the case for [8]CPP, this shift seems certainly accessible under the reaction conditions utilized for Scholl type chemistry, although perhaps slightly slower in this case. Surmising that the transition state is accessible, it is important to also compare the energies of aryl-substituted [8]CPP (**27**) and the product of an aryl shift (**27e**). We found that the shifted macrocycle (**27e**) lies lower in energy by 22.36 kcal mol^–1^ as compared to the starting CPP (**27**), indicating that an extremely large driving force exists for these rearrangements. We also anticipate that there are many lower energy isomers that contain multiple strain relieving shifts, such as those found for [8]CPP (**22f** and **22j**).^61^

## Conclusion

Since recently synthesized, the [*n*]cycloparaphenylenes have garnered significant interest as materials for a variety of next generation applications. To realize their full potential in many of these proposed applications, the CPPs must be chemically modified. Specifically, we propose that pi-extended cycloparaphenylenes such as **5** are necessary for the successful utilization of the CPPs as templates for homogenous CNT growth. Towards this, we have attempted to develop a methodology based upon the Scholl reaction for the synthesis of CNT **5** from aryl substituted CPP **8**. Through our investigations of model systems **15**, **16**, and **19** we have arrived at the conclusion that both cationic and radical cationic reactions are problematic due to the high strain of the CPPs. We propose that radical cations of cycloparaphenylenes undergo ring-openings to afford insoluble linear oligophenylenes. Furthermore, we have shown that cationic conditions provide 1,2-phenyl shifted isomers of the CPP, with rearranged isomer **23** characterized unambiguously by X-ray diffraction.

The mechanism of cationic rearrangement has been investigated computationally. The 1,2-aryl shift has a calculated activation energy barrier of 21–24 kcal mol^–1^, with an approximately 20 kcal mol^–1^ stabilization associated with each strain relieving rearrangement. This holds true for both standard CPPs and highly aryl-substituted CPPs. When considering the viability of conducting Scholl type chemistry on a substrate such as **8** to synthesize CNT **5**, it is important to understand that 36 C–C bond closures must occur before a single 1,2-aryl shift.

In a broader sense, we note that there have been very few reports of successful reactions upon the CPPs since first accessed in 2008. Underscoring this, our mechanistic study has demonstrated that cationic intermediates that are common to many reaction pathways (*e.g.* halogenation, nitration, *etc.*) will pose problems in these cyclic systems. Given these results, we propose that synthetic transformations that avoid cationic or radical cationic intermediates are preferred for the successful realization of CNT **5**. Development of these alternative strategies towards CNT **5** are underway in our laboratory and will be reported in due course.

## Supplementary Material

Supplementary information

Crystal structure data
