# Supplementary material for: Towards pi-extended cycloparaphenylenes as seeds for CNT growth: investigating strain relieving ring-openings and rearrangements
Source: Chem Sci. 2016 Feb 18;7(6):3681–8. doi: 10.1039/c5sc04218f (PMC6008588; doi:10.1039/c5sc04218f)

Electronic Supplementary Material (ESI) for Chemical Science.
This journal is © The Royal Society of Chemistry 2016

TOWARDS PI-EXTENDED CYCLOPARAPHENYLENES AS SEEDS FOR CNT GROWTH: INVESTIGATING STRAIN RELIEVING RING-OPENINGS AND REARRANGEMENTS

Thomas J. Sisto†, Lev Zakharov‡, Brittany M. White†, and Ramesh Jasti*,†

†Department of Chemistry & Biochemistry and Materials Science Institute, University of Oregon, Eugene, Oregon 97403-1253, United States

‡Camcor, University of Oregon, Eugene, Oregon 97403-1253

Table Of Contents

1) **General Experimental……………………………………….…………S2-S3**
2) **Procedures and Characterization………………………………….S4-S20**
3) **Spectra………………………………………………………………..S21-S31**
4) **Triflic Acid Scholl Reaction Procedures and Characterization…S32-43**
5) **Computational Data…………………………...………………S44-104**
6) **X-Ray Crystallography Data…………………………………S105-106**
7) **CNT Yield from CPP Templated Growth…………………………...S107**
8) **HOMO Orbital Coefficients of Fully Aryl-Substituted 12……………......S107**

General Experimental Details. ^{1}H NMR spectra were recorded at 500 MHz on a Varian VNMRS, at 400 MHz on a Varian VNMRS, or at 600 MHz on a Bruker Avance-III-HD spectrometer. ^{13}C NMR spectra were recorded at 125 MHz on a Varian VNMRS, 100 MHz on a Varian VNMRS, or 150 MHz on a Bruker Avance-III-HD spectrometer. All NMR spectra in $CDCl_3$ were referenced to TMS. All reagents were obtained commercially. Tetrahydrofuran, dicholoromethane, and dimethylformamide were dried by filtration through alumina according to the methods described by Grubbs[1]. Silica column chromatography was conducted with Zeochem Zeoprep 60 Eco 40-63 μm silica gel. Thin Layer Chromatography (TLC) was performed using Sorbent Technologies Silica Gel XHT TLC plates or Sorbent Technologies Alumina TLC plates respectively. Developed plates were visualized using UV light at wavelengths of 254 and 365 nm. Gel permeation chromatography was performed using a Japan Analytical Industry Co. Ltd. recycling preparative HPLC fitted with Jaigel-2H polystyrene/divinylbenzene columns at a flow rate of 3.5 mL/min. All glassware was oven or flame dried and cooled under an inert atmosphere of nitrogen unless otherwise noted. Moisture sensitive reactions were carried out under an inert atmosphere of nitrogen using standard syringe/septa technique. Calculations were performed using Gaussian 09. All structures were optimized at the B3LYP/6-31+G(d,p) or B3LYP/6-31(d,p) as noted. Vibrational analysis was conducted for all structures, with minima displaying no imaginary modes and transition states displaying one imaginary mode.

---

[1] Pangborn, A. B.; Giardello, M. A; Grubbs, R. H.; Rosen, R. K.; Timmers, F. J. *Organometallics* **1996,** *15,* 1518-1520.

Complete Gaussian Reference:

Gaussian 09, Revision B.01, M. J. Frisch, G. W. Trucks, H. B. Schlegel, G. E. Scuseria, M. A. Robb, J. R. Cheeseman, G. Scalmani, V. Barone, B. Mennucci, G. A. Petersson, H. Nakatsuji, M. Caricato, X. Li, H. P. Hratchian, A. F. Izmaylov, J. Bloino, G. Zheng, J. L. Sonnenberg, M. Hada, M. Ehara, K. Toyota, R. Fukuda, J. Hasegawa, M. Ishida, T. Nakajima, Y. Honda, O. Kitao, H. Nakai, T. Vreven, J. A. Montgomery, Jr., J. E. Peralta, F. Ogliaro, M. Bearpark, J. J. Heyd, E. Brothers, K. N. Kudin, V. N. Staroverov, T. Keith, R. Kobayashi, J. Normand, K. Raghavachari, A. Rendell, J. C. Burant, S. S. Iyengar, J. Tomasi, M. Cossi, N. Rega, J. M. Millam, M. Klene, J. E. Knox, J. B. Cross, V. Bakken, C. Adamo, J. Jaramillo, R. Gomperts, R. E. Stratmann, O. Yazyev, A. J. Austin, R. Cammi, C. Pomelli, J. W. Ochterski, R. L. Martin, K. Morokuma, V. G. Zakrzewski, G. A. Voth, P. Salvador, J. J. Dannenberg, S. Dapprich, A. D. Daniels, O. Farkas, J. B. Foresman, J. V. Ortiz, J. Cioslowski, and D. J. Fox, Gaussian, Inc., Wallingford CT, 2010.

Procedures and Characterization.

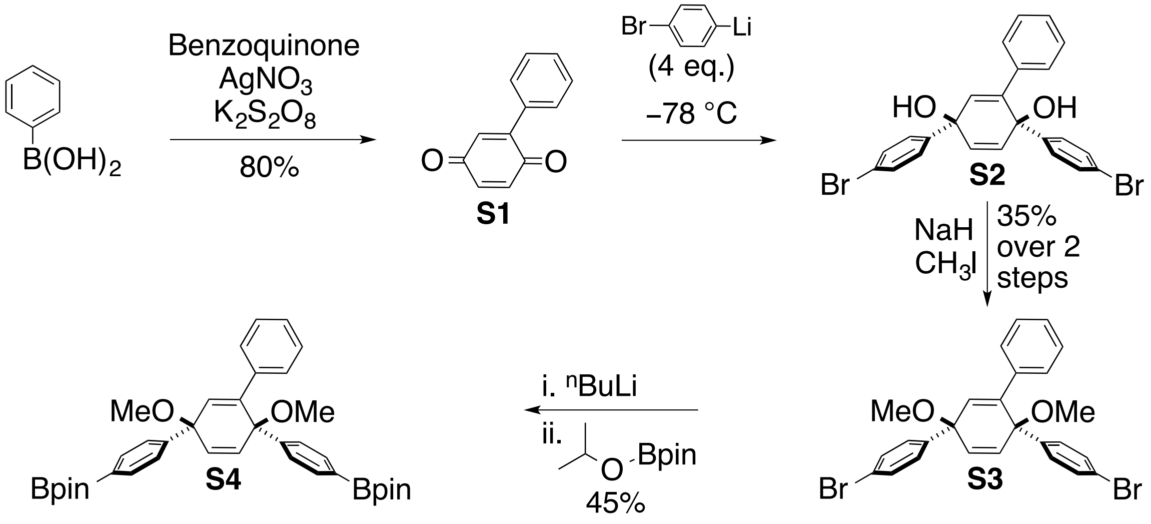

Figure S1. Synthetic route to mono-aryl [8]CPP **19**.

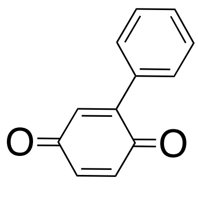

2-Phenyl-1,4-benzoquinone S1. A procedure outlined by Baran was followed[2]. Purification was achieved using silica gel chromatography (hexanes/ethyl acetate, 9:1), followed by recrystallization in hot hexanes. All spectroscopic data was consistent with those reported in the literature.

---

[2] Fujiwara, Y.; Domingo, V.; Seiple, I.B.; Gianatassio, R.; Del Bel, M.; Baran, P. S. *J. Am. Chem. Soc.* **2011**, *133*, 3292-3295

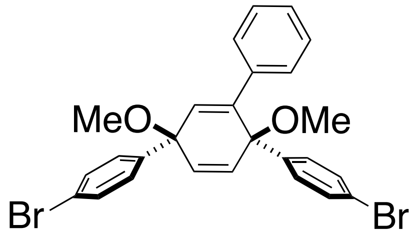

Dibromide S3. To a dry 500 mL round bottom flask charged with a large magnetic stir bar, 1,4-dibromobenzene (16.15 g, 68.46 mmol, 4.2 equiv), and THF (300 mL) at −78 °C was added 2.5 M ^{n}BuLi dropwise (28 mL, 70.09 mmol, 4.3 equiv), at which point the reaction mixture became a slurry. The slurry was allowed to stir for 30 minutes at −78 °C. This reaction mixture was cannulated dropwise into a separately cooled (−78 °C) dry 1 L round bottom flask charged with a spinning magnetic stir bar, quinone **S1** (3 g, 16.3 mmol, 1 equiv), and dry THF (360 mL), at which point the new reaction mixture was allowed to continue to stir for 15 minutes. Upon completion, the reaction mixture was quenched with H_2O (150 mL) and allowed to warm to room temperature. Upon seperation of the layers, the aqueous layer was extracted with ethyl acetate (3 X 150 mL) and the combined organic layers were washed with a saturated brine solution (150 mL) and dried over sodium sulfate. Concentration under reduced pressure afforded a brown oil that was crudely purified (this crude purification is not necessary, however subsequent purification after the next step becomes significantly easier) via silica gel chromatography (flushed with 1:9 ethyl acetate:hexanes and eluted with 5:5 ethyl acetate:hexanes) to afford a semi-crude brown diol (**S2**) that was used in the next reaction.

To a dry 250 mL round bottom flask was added a stir bar, sodium hydride (2.608 g as a 60% suspension in oil, 65.2 mmol, 4 equiv), and dry THF (130 mL). This slurry was cooled to 0 °C, at which point the semi-crude diol (**S2**) was introduced dropwise as a solution in dry THF (20 mL). The reaction mixture was allowed to stir for 30 minutes at 0°C. Neat methyl iodide was then injected as a stream (13.8 g, 6.06 mL, 97.8 mmol, 6 equiv) and the reaction mixture was allowed to warm to room temperature and stir for 16 hours. The excess sodium hydride was quenched slowly by the addition of H_2O (50 mL) and the layers were separated. The aqueous layer was extracted with ethyl acetate (3 X 50 mL) and the combined organic layers were washed with a saturated brine solution (100 mL), dried over sodium sulfate, and concentrated under reduced pressure to afford a crude oil. The crude oil was purified by silica gel chromatography (0:10 to 1:9 dichloromethane:hexanes) to afford **S3** as a colorless oil (3.1 g, 36%) IR (neat) 3055.62, 3028.78, 2985.25, 2938.62, 2823.21, 2246.63, 1586.82, 1484.16, 1395.28, 1171.56, 1072.42, 1009.09, 951.27, 907.31, 826.09, 730.29 cm^{-1}; ^1H NMR (400 MHz, $CDCl_3$) δ 7.48 (d, J = 8.38 Hz, 2H), 7.35 (overlap, 4H), 7.27-7.23 (overlap, 2H), 7.21-7.16 (overlap, 5H), 6.48 (d, J = 1.52 Hz, 1H), 6.06 (dd, J = 9.99, 1.52 Hz, 1H), 6.02 (d, J = 9.99 Hz, 1H), 3.46 (s, 3H), 3.35 (s, 3H); ^{13}C NMR (400 MHz, $CDCl_3$) δ 142.48, 142.32, 140.52, 137.57, 136.96, 134.42, 131.64, 131.42, 129.48, 128.08, 127.99, 127.95, 127.88, 127.77, 121.95, 121.36, 77.55, 75.44, 52.07, 51.73; HRMS was attempted by ESI or MALDI for this compound, however no sample ionized. Subsequent transformations of this compound are characterized with HRMS.

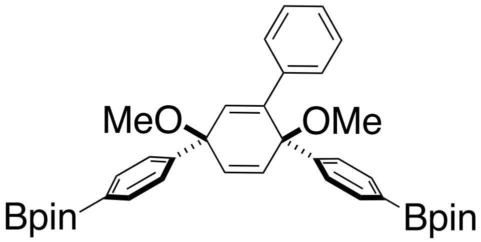

Diboronate S4. To a 50 mL dry roundbottom flask charged with a dry stirbar, dibromide **S3** (500 mg, 0.95 mmol, 1 equiv) and THF (10 mL) at −78 °C was introduced 2.5 M ^{n}BuLi (0.836 mL, 2.09 mmol, 2.2 equiv.) dropwise over the course of 2 minutes. The reaction was stirred an additional 30 seconds, at which point neat isopropyl pinacol borate (707 mg, 0.775 mL, 3.8 mmol, 4 equiv.) was introduced quickly as a stream[3]. Upon introduction of isopropyl pinacol borate the reaction mixture was stirred for 1 hour at −78 °C, at which point the reaction was quenched with H_2O (20 mL) and allowed to warm to room temperature. The reaction mixture was diluted with ethyl acetate (30 mL), the layers were separated, and the aqueous layer was extracted (3 X 20 mL) with ethyl acetate. The organic layers were combined, washed with brine, and dried over sodium sulfate before being evaporated under reduced pressure to afford a crude oil. This oil was purified via silica gel chromatography (1:19 ethyl acetate:hexanes) to afford **S4** as an oil (265 mg, 45%). IR (neat) 2978.67, 2933.95, 2823.93, 1609.31, 1397.89, 1358.54, 1319.91, 1270.52, 1143.58, 1086.59, 1018.18, 950.54, 858.61, 737.26 cm^{-1}; ^{1}H NMR (500 MHz, $CDCl_3$) δ 7.83 (d, *J* = 8.45 Hz, 2H), 7.71 (d, *J* = 8.45 Hz, 2H), 7.45 (d, *J* = 8.45 Hz, 2H), 7.40 (d, *J* = 8.45 Hz, 2H) 7.29-7.27 (overlap, 2H), 7.19-7.13 (overlap, 3H),

---

3 The timing associated with the introduction, subsequent stir, and quench of ^{n}BuLi with isopropyl pinacol borate is critical to the success of the reaction.

6.56 (d, J = 2.23, 1H), 6.11 (dd, J = 10.05, 2.23 Hz, 1H), 6.05 (d, J = 10.05 Hz, 1H), 3.50 (s, 3H), 3.39 (s, 3H), 1.38 (s, 6H), 1.37 (s, 6H), 1.36 (s, 6H), 1.35 (s, 6H); ^{13}C NMR (500 MHz, $CDCl_3$) δ 146.47, 146.45, 140.53, 138.05, 136.83, 135.04, 134.83, 134.54, 129.45, 127.93, 127.85, 127.65, 125.60, 125.45, 83.79, 83.74, 78.11, 75.92, 51.95, 51.63, 24.94, 24.87, C-B signals not observed; HRMS (Q-TOF ES+) *m/z* calculated for $C_{37}H_{44}B_2O_5$ (M − OMe)$^+$ 589.3309; found, 589.3297.

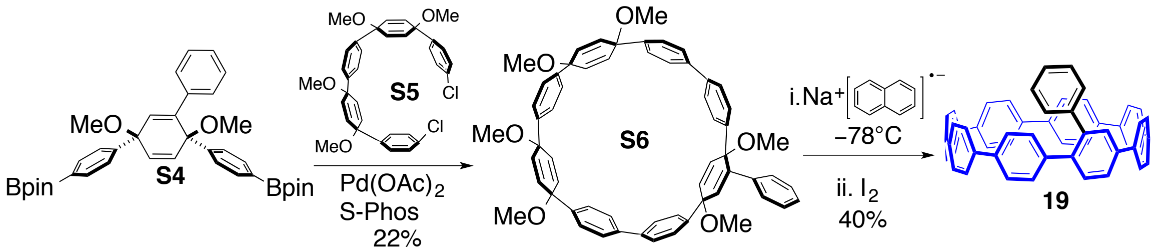

Figure S2. Synthetic route to mono-aryl [8]CPP **19**.

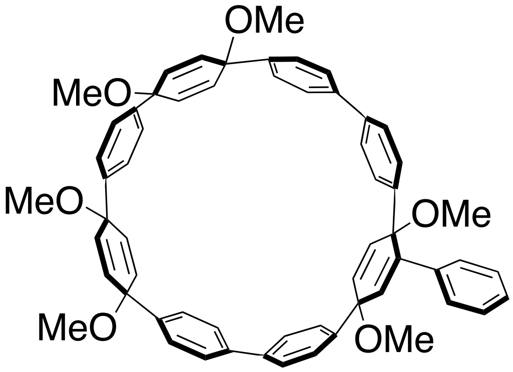

Macrocycle S6. A 100 mL schlenck flask was charged with a stirbar, dichloride **S5**[4] (92.8 mg, 0.161 mmol, 1 equiv.), diboronate **S4** (100 mg, 0.161 mmol, 1 equiv.), $Pd(OAc)_2$ (10.8 mg, 0.0481 mmol, 0.3 equiv), S-Phos (26.4 mg, 0.0644 mmol, 0.4 equiv.), and Cs_2CO_3 (261.6 mg, 0.805 mmol, 5 equiv.), to which was added sparged (N_2, 1 hour) dioxane (54 mL) and sparged (N_2, 1 hour) H_2O (6 mL). The reaction mixture was sealed and placed into a preheated oil bath at 80 °C and stirred for 16 hours. Upon completion the reaction mixture was cooled, diluted with dichloromethane (50 mL), filtered through celite, and placed in a separatory funnel containing H_2O (75 mL). The

[4] Dichloride **S5** was prepared as outlined in Li, P.; Sisto, T. J.; Darzi, E. R.; Jasti, R. *Org. Lett.* **2014**, *16*, 182-185

layers were separated, at which point the aqueous layer was washed with dichloromethane (3 X 25 mL). The organic layers were subsequently combined, washed with brine, dried over sodium sulfate, and evaporated under reduced pressure. The crude mixture was purified via silica gel chromatography (1:19 ethyl acetate:dichloromethane) to afford **S6** as a white solid (30 mg, 22%). IR (neat) 3028.85, 2982.23, 2933.25, 2896.86, 2822.76, 2246.29, 1491.15, 1448.94, 1397.35, 1174.43, 1077.54, 1016.70, 1005.88, 950.14, 908.69, 822.60, 729.84 cm^{-1}; ^{1}H NMR (500 MHz, $CDCl_3$) δ 7.54-7.47 (overlap, 12H), 7.42 (d, J = 8.49 Hz, 2H), 7.39-7.36 (m, 2H), 7.33 (d, 8.49 Hz, 2H), 7.27-7.24 (m, 2H), 7.21-7.18 (overlap, 3H), 7.11 (d, J = 8.49 Hz, 2H), 6.75 (d, J = 2.24 Hz, 1H), 6.27 (d, J = 10.01 Hz, 1H), 6.23 (dd, J = 10.01, 2.24 Hz, 1H), 6.17-6.02 (overlap, 8H), 3.52 (s, 3H), 3.48 (s, 3H), 3.47 (s, 3H), 3.41 (s, 3H), 3.40 (s, 3H), 3.20 (s, 3H); ^{13}C NMR (500 MHz, $CDCl_3$) δ 143.33, 142.85, 142.82, 140.96, 140.20, 140.15, 139.85, 139.71, 139.52, 138.13, 137.56, 135.76, 133.95, 133.50, 133.40, 133.19, 133.11, 132.83, 132.37, 128.90, 127.95, 127.63, 127.50, 127.08, 126.84, 126.73, 126.62, 126.52, 126.26 (3), 126.25 (3), 78.95, 76.36, 74.61, 74.56, 74.08, 74.06, 52.10, 52.03, 51.82, 51.72; MALDI-TOF *m/z* calculated for $C_{59}H_{52}O_5$ $(M - OMe)^+$ 840.3; found, 840.3.

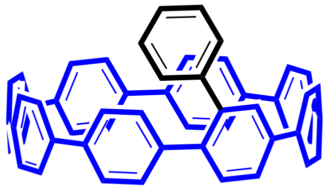

Mono-aryl [8]CPP 19. A solution of sodium napthalenide was freshly prepared by charging a dry 100 mL roundbottom with a dry stirbar, THF (60 mL), and sodium metal (690 mg, 30 mmol, 1 eq.). The solution was stirred for 5 minutes at room temperature, at

which point naphthalene (4.23 g, 33 mmol, 1.1 eq.) was added, immediately resulting in a dark green solution. The mixture was stirred for 3 hours at room temperature, at which point it was utilized.

While the sodium napthalenide was being prepared, a dry 25 mL roundbottom flask was charged with a dry stirbar, macrocycle **S6** (100 mg, 0.144 mmol, 1 eq.), and THF (10 mL). This reaction mixture was cooled to −78 °C, at which point sodium napthalenide (3 mL, 1.5 mmol, 10 eq.) was introduced dropwise, immediately resulting in a vivid purple reaction mixture that became dark green upon completion of introduction. The reaction mixture was stirred for 30 minutes at −78 °C, at which point the reaction was quenched with a solution of iodine in dry THF (titrated into reaction mixture until a red color persists). The reaction mixture was warmed to room temperature and transferred to a separatory funnel, where it was diluted with dichloromethane (50 mL) and a solution of saturated sodium thiosulfate (enough to quench the excess iodine). The layers were separated, at which point the aqueous layer was extracted with dichloromethane (3 X 20 mL). The organic layers were combined, washed with brine, and dried over sodium sulfate. The organic layer was then evaporated under reduced pressure to afford a crude yellow solid, which was crudely purified by silica column chromatography (0:10 to 50:50 dichloromethane:hexanes). This material was then crystallized by supersaturation (sonication) in dichloromethane followed by careful introduction of a top layer of hexanes (32 mg, 40%). IR (neat) 3075.80, 3022.13, 2246.83, 1898.87, 1584.53, 1481.06, 1443.53, 1390.91, 906.39, 812.15, 727.01 cm^{-1}; ^{1}H NMR (500 MHz, $CDCl_3$) δ 7.92 (d, J = 1.82 Hz, 1H), 7.74-7.72 (overlap, 2H) 7.62-7.55 (overlap, 6H), 7.53-7.46 (overlap,

16H), 7.44-7.32 (overlap, 6H), 7.25 (s broad, 1H), 7.17-7.11 (overlap broad, 3H), 7.07 (d, *J* = 8.50 Hz, 1H); ^{13}C NMR (500 MHz, $CDCl_3$) δ 141.41, 140.11, 139.68, 138.24, 138.22, 138.09, 137.97, 137.77, 137.75, 137.74, 137.72, 137.67, 137.65, 137.61, 137.57, 137.50, 137.43, 134.68, 129.80, 129.50, 128.78, 127.51 (12), 127.48, 127.42, 127.36, 127.29, 125.25; MALDI-TOF *m/z* calculated for $C_{54}H_{36}$ $(M)^+$ 684.88; found, 685.3.

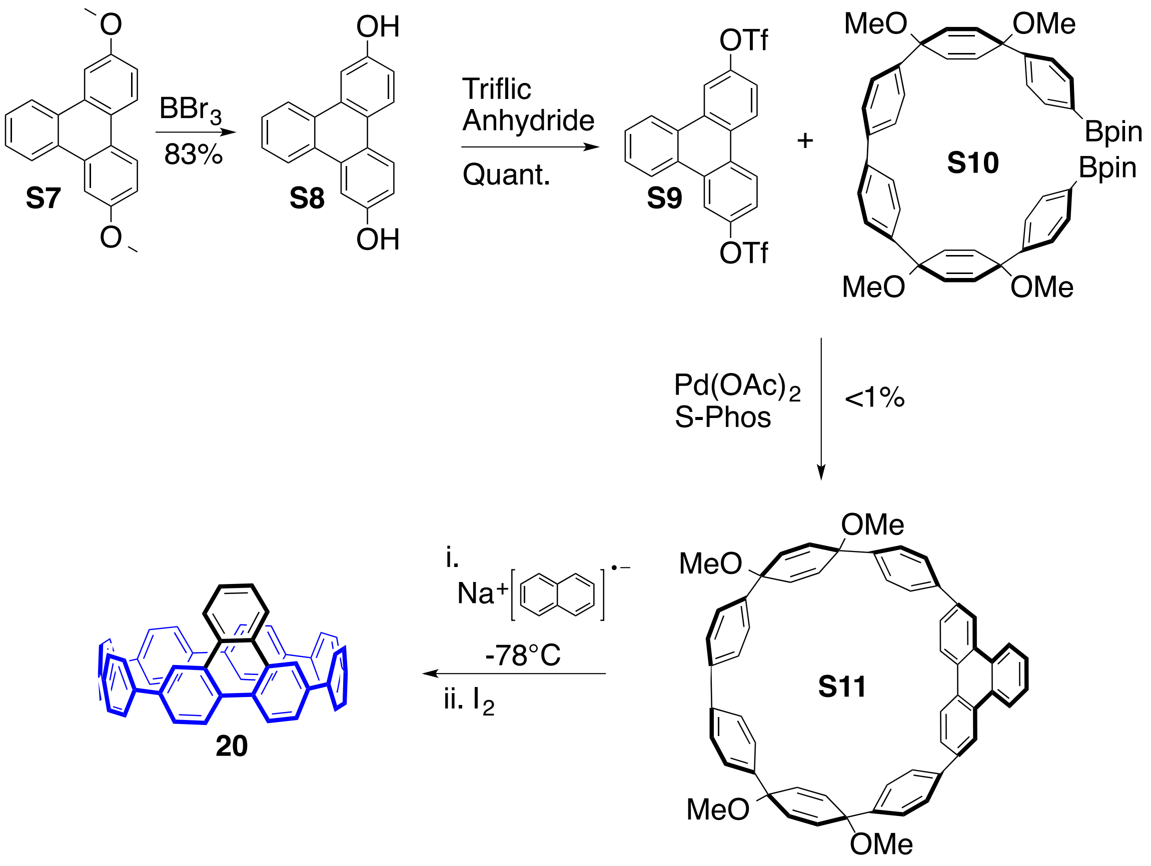

Figure S3: Synthetic route to authentic sample **20**.

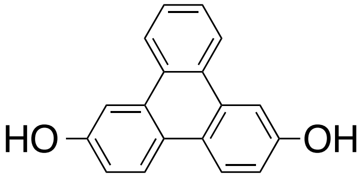

Dihydroxytriphenylene S8: CAUTION: BBr_3 liberates HBr gas upon quench. Be aware that a large volume of gas is liberated quickly from a small volume of liquid. Use appropriately sized glassware, and vent the liberated gas through glass adaptors to a pH basic bubbler quench.

To a 100 mL dry roundbottom flask (advise using a larger multi-necked flask) charged with a dry stirbar, was added literature known dimethoxytriphenylene[5] **S7** (1 gram, 3.47 mmol, 1 eq.). To the reaction flask, 50 mL of dry dichloromethane was added. The reaction mixture was cooled to 0 °C. Once cooled, boron tribromide (13.03 g, 4.93 mL, 52.02 mmol, 15 eq.) was added dropwise. The reaction mixture was stirred for 4 hours, at which point water was added extremely slowly (CAUTION). The reaction mixture was subsequently placed into a separatory funnel and diluted with water and ethyl acetate (150 mL of each). The organic layer was separated, and the aquoues layer was extracted with ethyl acetate (3 X 50 mL). The organic layers were combined, washed with brine, and dried over sodium sulfate. Once dry, the organic layer was evaporated under reduced pressure to afford a solid, which was washed with chloroform to afford a white solid (750 mg, 83%). IR (neat): 3263.31, 1613.16, 1584.97, 1502.47, 1428.99, 1363.86, 1338.26, 1286.34, 1242.02,1188.40, 901.57869.14, 856.58, 848.74, 804.21, 759.77 cm^{-1}; ^{1}H NMR (500 MHz, DMSO-d_6) δ 9.73 (s, 2H), 8.52 (dd, AAB', *J* = 6.92, 3.65 Hz, 2H), 8.44 (d, *J* = 8.88 Hz, 2H), 7.95 (d, *J* = 2.47 Hz, 2H), 7.64 (dd, AAB', *J* = 6.92, 3.65 Hz, 2H), 7.12 (dd, *J* = 8.88, 2.47 Hz, 2H); ^{13}C NMR (600 MHz, DMSO-d_6) δ 156.45, 129.82, 129.70, 127.79, 124.87, 123.85, 122.88, 117.64, 108.05; HRMS (Q-TOF ES+) *m/z* calculated for $C_{18}H_{12}O_2$ $(M)^+$ 260.08373; found 260.08365.

5 King, B. T.; Kroulik, J.; Robertson, C. R.; Rempala, P.; Hilton, C. L.; Korinek, J. D.; Gortari, L. M. *J. Org. Chem.* **2007**, *72*, 2279-2288

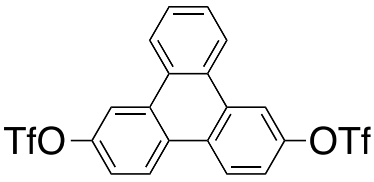

Di-triflatetriphenylene S9: A 100 mL dry roundbottom flask was charged with a dry stirbar, at which point **S8** (500 mg, 1.92 mmol, 1 eq.) was added. To the vessel was added dichloromethane (15 mL) and distilled pyridine (15 mL), at which point the reaction mixture was cooled to 0 °C. Once cool, triflic anyhydride (2.166 g, 1.3 mL, 7.68 mmol, 4 eq) was added dropwise. The reaction was allowed to warm to room temperature and stirred for one hour. The reaction was then quenched dropwise with H_2O. Once quenched, the reaction mixture was transferred to a separatory funnel and diluted with ethyl acetate (75 mL) and H_2O (75 mL). The layers were separated, and the aqueous layer was extracted with ethyl acetate (3 X 50 mL). The organic layers were combined, washed with brine, and dried over sodium sulfate. Once dry, the organic layer was evaporated under reduced pressure to afford a solid. This solid was eluted with dichloromethane through a pad of silica to afford a white solid (quantitative). IR (neat) 1615.33, 1582.73, 1497.63, 1437.09, 1417.82, 1243.57, 1206.07, 1137.92, 1030.45, 926.18, 869.48, 828.53, 805.08, 758.67, 650.95, 624.93, 596.05, 574.70; ^{1}H NMR (500 MHz, DMSO-d_6) δ 8.63 (d, *J* = 9.10 Hz, 2H), 8.51 (dd, ABB' *J* =6.78, 3.53 Hz, 2H), 8.47 (d, *J* = 2.46 Hz, 2H), 7.77 (dd, AAB', *J* = 6.78, 3.53 Hz, 2H), 7.58 (dd, *J* = 9.10, 2.46 Hz, 2H); ^{13}C NMR (150 MHz, DMSO-d_6) δ 149.11, 131.79, 128.93, 128.85, 128.24, 126.00, 123.61, 120.39, 118.85 (q, *J* = 321 Hz, 4th peak overlapped), 115.96; HRMS (Q-TOF ES+) *m/z* calculated for $C_{20}H_{10}O_6S_2F_6$ $(M)^+$ 523.98229; found 523.98089.

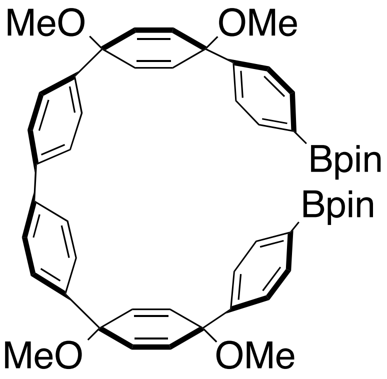

6-Ring Diboronate S10: A dry 25 mL flask was charged with a large (reaction is heterogeneous) dry stirbar, previously reported 6-ring dichloride[6] (1 g, 1.535 mmol, 1 eq.), bis(pinacolato)diboron (2.34 g, 9.21 mmol, 6 eq.), $Pd(OAc)_2$ (72.5 mg, 0.324 mmol, 0.21 eq.), S-Phos (251.7 mg, 0.614 mmol, 0.4 eq.), and oven dried, ground K_3PO_4 (1.953 g, 9.21 mmol, 6 eq.). The flask was evacuated and backfilled with N_2 (6X). Degassed DMF (7 mL; sparged with N_2 serarately) was added to the reaction mixture, which was subsequently heated to 60 °C. The heterogeneous mixture was allowed to stir for 16 hours, at which point it was cooled. Once cooled, the reaction mixture was dissolved in dichloromethane, passed through a pad of celite, and concentrated under reduced pressure to afford a solid. This solid was washed with hexanes to remove the excess bis(pinacolato)diboron and afford clean product (850 mg, 66%). IR (neat) 2978.16, 2935.75, 2822.47, 1609.60, 1491.38, 1398.22, 1360.73, 1320.34, 1271.92, 1144.77, 1087.09, 1018.68, 950.59, 858.91, 823.19, 760.67, 739.31, 659.23, 539.99; ^{1}H NMR (600 MHz, $CDCl_3$) δ 7.83 (d, J = 8.66 Hz, 4H), 7.58 (d, J = 8.66 Hz, 4H), 7.51 (d, J = 8.66 Hz,

---

6 Sisto, T. J.; Golder, M. R.; Hirst, E. S.; Jasti, R. *J. Am. Chem. Soc.* **2011**, *133*, 15800-15802

4H), 7.49 (d, *J* = 8.66 Hz, 4H), 6.20 (d, *J* = 10.37 Hz, 4H), 6.16 (d, *J* = 10.37 Hz, 4H), 3.50 (s, 6H), 3.49 (s, 6H), 1.38 (s, 24H); ^{13}C NMR (150 MHz, DMSO-d_6) δ 146.48, 142.48, 140.07, 134.98, 133.41, 133.32, 127.17, 126.46, 125.38, 83.79, 75.00, 74.75, 52.04, 52.01, 24.90; HRMS (Q-TOF ES+) *m/z* calculated for $C_{52}H_{60}B_2O_8Na$ $(M + Na)^+$ 857.4372; found 857.4387.

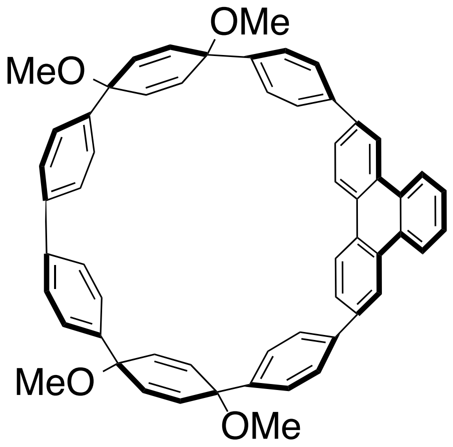

Triphenylene [8]macrocycle S11: A 100 mL schlenck flask was charged with a stirbar, ditriflate **S9** (73.4 mg, 0.14 mmol, 1.2 equiv.), diboronate **S10** (100 mg, 0.12 mmol, 1 equiv.), $Pd(OAc)_2$ (6 mg, 0.024 mmol, 0.21 equiv), S-Phos (20 mg, 0.048 mmol, 0.4 equiv.), and K_3PO_4 (78.2 mg, 0.24 mmol, 2 equiv.), to which was added sparged (N_2, 1 hour) DMF (54 mL) and sparged (N_2, 1 hour) H_2O (6 mL). The reaction mixture was sealed and placed into a preheated oil bath at 120 °C and stirred for 16 hours. Upon completion the reaction mixture was cooled, diluted with dichloromethane (50 mL), filtered through celite, and placed in a separatory funnel containing H_2O (75 mL). The layers were separated, at which point the aqueous layer was extracted with dichloromethane (3 X 25 mL). The organic layers were subsequently combined, washed

with brine, dried over sodium sulfate, and evaporated under reduced pressure. The crude mixture was purified via silica gel chromatography (1:19 ethyl acetate:dichloromethane), followed by preparatory TLC (1:19 ethyl acetate:dichloromethance) to afford **S11** as a white solid (<1 mg, 1%). IR (neat) 3028.00, 2933.39, 2821.27, 2245.57, 1604.33, 1489.91, 1441.51, 1395.47, 1227.19, 1173.88, 1080.41, 1026.21, 1016.94, 1005.27, 950.19, 907.38, 842.08, 821.41, 804.97, 762.52, 730.63, 666.08 cm^{-1}; NMR (500 MHz, $CDCl_3$) δ 8.41 (d, *J* = 8.74 Hz, 2H), 8.38 (dd, ABB', *J* = 7.11, 3.90 Hz, 2H), 8.21 (d, *J* = 1.60 Hz, 2H), 7.76 (dd, *J* = 8.74, 1.60 Hz, 2H), 7.67 (dd, ABB', *J* = 7.11, 3.90 Hz, 2H), 7.45 (d, *J* = 8.65 Hz, 4H), 7.42 (d, *J* = 8.65 Hz, 4H), 7.17 (d, *J* = 8.41 Hz, 4H), 7.06 (d, *J* = 8.41 Hz, 4H), 6.24 (d, *J* = 10.52 Hz, 4H), 5.98-5.92 (overlap, 4H), 3.45 (s, 6H), 3.38 (s, 6H). ^{13}C NMR of acceptable signal to noise was unobtainable overnight in a shigemi tube due to the extremely low quantity of sample obtained. However, the compound is confirmed through HRMS, and subsequent transformations of this compound are charaterized. HRMS (Q-TOF ES+) *m/z* calculated for $C_{58}H_{46}O_4Na$ $(M+Na)^+$ 829.3294; found 829.3303.

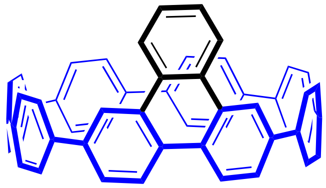

Authentic Sample 20: A solution of sodium naphthalenide was freshly prepared by charging a dry 100 mL roundbottom with a dry stirbar, THF (60 mL), and sodium metal (690 mg, 30 mmol, 1 eq.). The solution was stirred for 5 minutes at room temperature, at which point naphthalene (4.23 g, 33 mmol, 1.1 eq.) was added, immediately resulting in a

dark green solution. The mixture was stirred for 3 hours at room temperature, at which point it was utilized.

While the sodium naphthalenide was being prepared, a dry 5 mL roundbottom flask was charged with a dry stirbar, macrocycle **S11** (<1 mg), and THF (3 mL). This reaction mixture was cooled to −78 °C, at which point sodium napthalenide (1 mL, 0.5 mmol) was introduced dropwise, immediately resulting in a pink reaction mixture that became dark green upon completion of introduction. The reaction mixture was stirred for 30 minutes at −78 °C, at which point the reaction was quenched with a solution of iodine in dry THF (titrated into reaction mixture until a red color persists). The reaction mixture was warmed to room temperature and transferred to a separatory funnel, where it was diluted with dichloromethane (10 mL) and a solution of saturated sodium thiosulfate (enough to quench the excess iodine). The layers were separated, at which point the aqueous layer was extracted with dichloromethane (3 X 10 mL). The organic layers were combined, washed with brine, and dried over sodium sulfate. The organic layer was then evaporated under reduced pressure to afford a crude yellow solid, which was purified by silica column chromatography (0:10 to 50:50 dichloromethane:hexanes) to afford a yellow solid with an extremely distinct green fluorescence (<1 mg). IR (neat) 2920.33, 2850.22, 812.70, 731.07; NMR (500 MHz, $CDCl_3$) δ 8.30 (dd, ABB', *J* = 7.16, 3.90 Hz, 2H), 8.23 (d, *J* = 8.95 Hz, 2H), 8.18 (d, *J* = 1.80 Hz, 2H), 7.86 (dd, *J* = 8.95, 1.80 Hz, 2H), 7.65 (dd, ABB', *J* = 7.16, 3.90 Hz, 2H), 7.62 (d, *J* = 8.52 Hz, 4H), 7.48-7.40 (overlap, 14H), 7.21-7.08 (broad, 6H); ^{13}C NMR of acceptable signal to noise was unobtainable

overnight in a shigemi tube due to the extremely low quantity of sample obtained. MALDI-TOF *m/z* calculated for $C_{548}H_{34}$ $(M)^+$ 682.27; found 682.03.

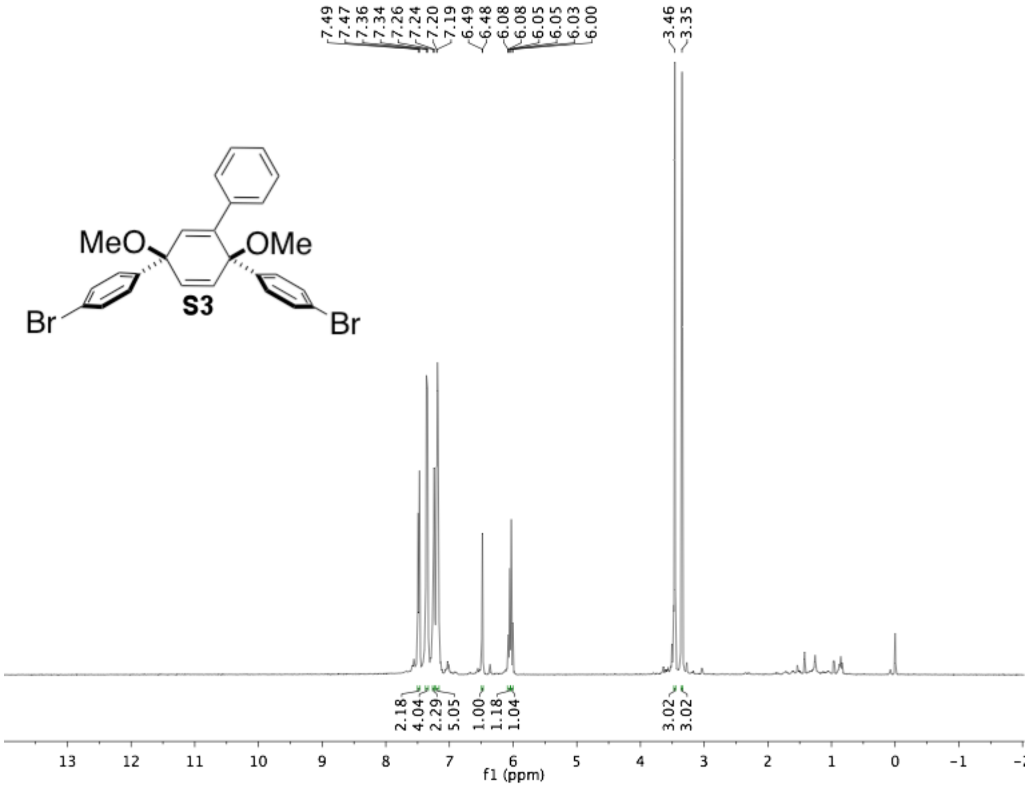
7.49
7.47
7.36
7.34
7.26
7.24
7.20
7.19
6.49
6.48
6.08
6.08
6.05
6.05
6.03
6.00
3.46
3.35
MeO
OMe
Br
Br
S3
2.18
4.04
2.29
5.05
1.00
1.18
1.04
3.02
3.02
f1 (ppm)

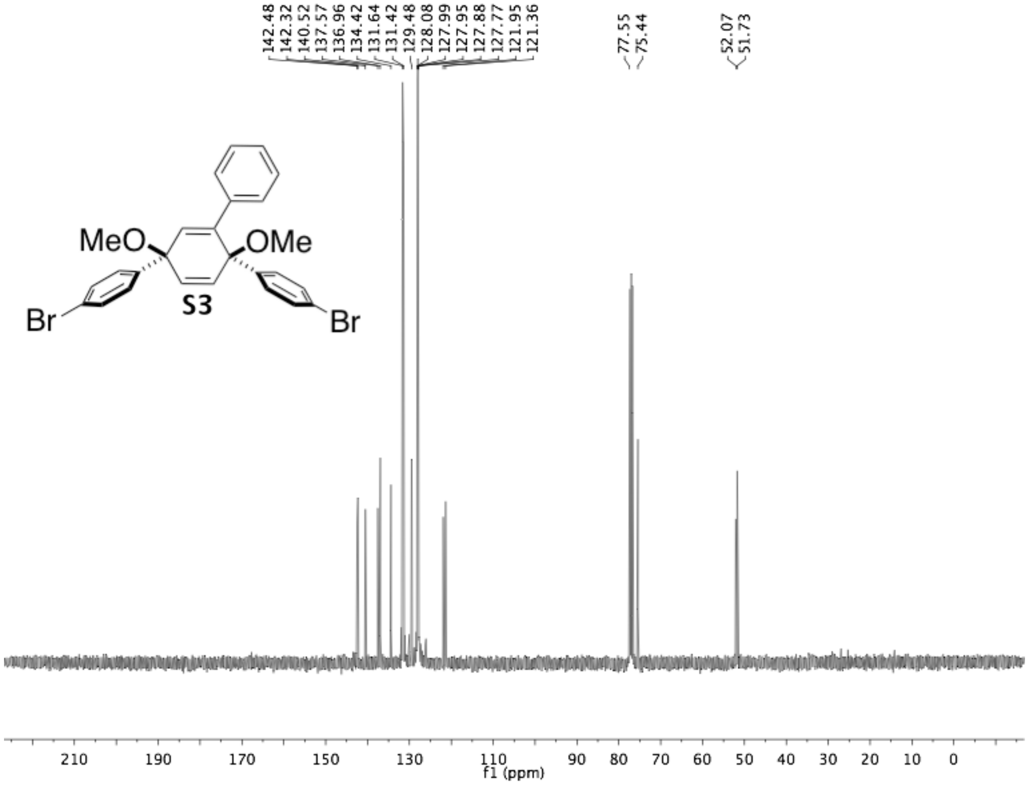
142.48
142.32
140.52
137.57
136.96
134.42
131.64
131.42
129.48
128.08
127.99
127.95
127.88
127.77
121.95
121.36
77.55
75.44
52.07
51.73
MeO
OMe
Br
Br
S3
f1 (ppm)

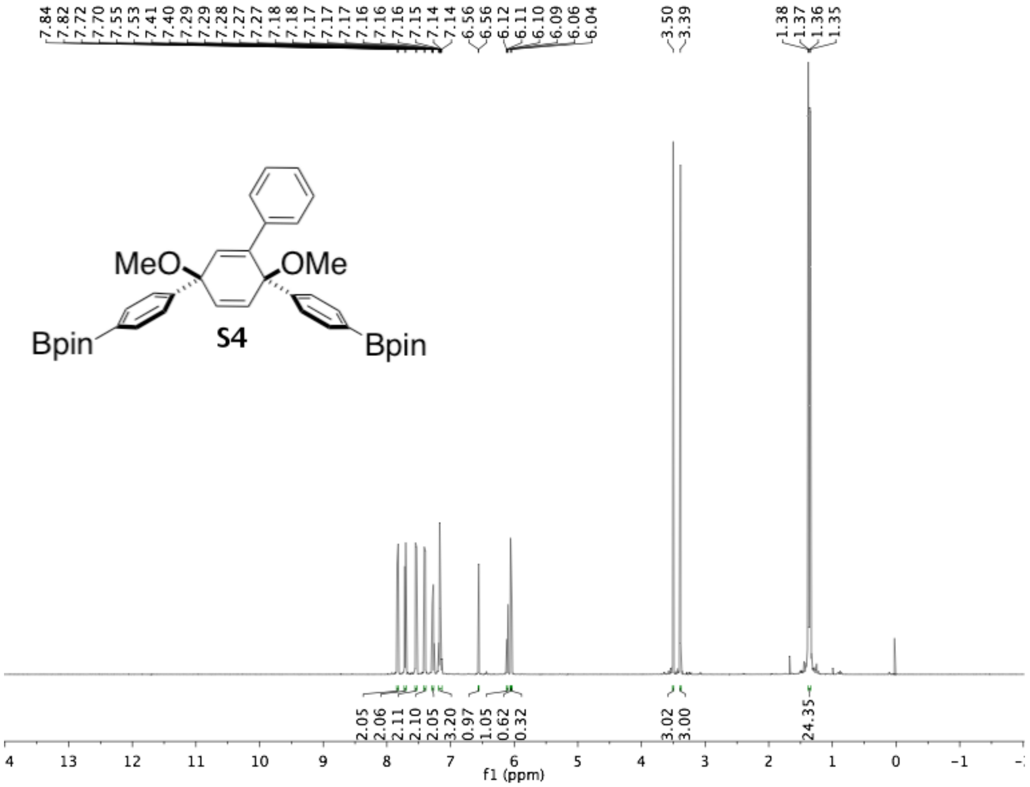
7.84
7.82
7.72
7.70
7.55
7.53
7.41
7.40
7.29
7.29
7.28
7.27
7.27
7.18
7.18
7.17
7.17
7.17
7.16
7.16
7.16
7.15
7.14
7.14
6.56
6.56
6.12
6.11
6.10
6.09
6.06
6.04
3.50
3.39
1.38
1.37
1.36
1.35
MeO
OMe
Bpin
S4
Bpin
2.05
2.06
2.11
2.10
2.05
3.20
0.97
1.05
0.62
0.32
3.02
3.00
24.35
f1 (ppm)

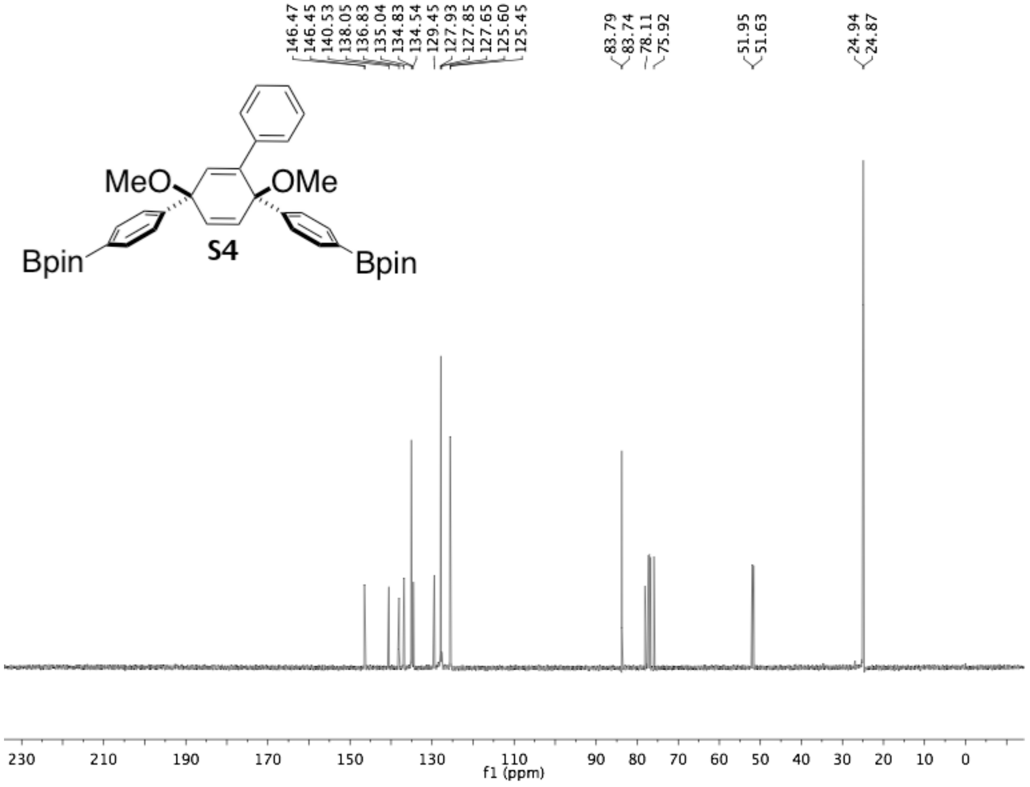
146.47
146.45
140.53
138.05
136.83
135.04
134.83
134.54
129.45
127.93
127.85
127.65
125.60
125.45
83.79
83.74
78.11
75.92
51.95
51.63
24.94
24.87
MeO
OMe
Bpin
S4
Bpin
f1 (ppm)

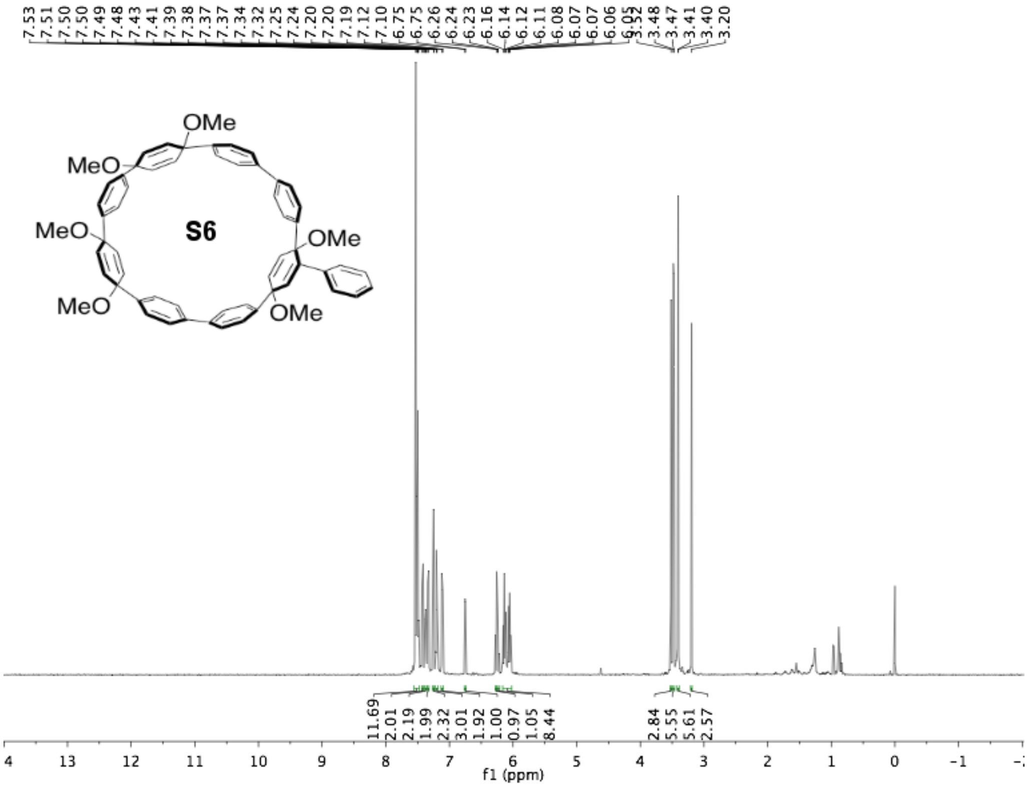
7.53
7.51
7.50
7.50
7.49
7.48
7.43
7.41
7.39
7.38
7.37
7.37
7.34
7.32
7.25
7.24
7.20
7.20
7.19
7.12
7.10
6.75
6.75
6.26
6.24
6.23
6.16
6.14
6.12
6.11
6.08
6.07
6.07
6.06
6.05
3.52
3.48
3.47
3.41
3.40
3.20
OMe
MeO
MeO
MeO
S6
OMe
OMe
11.69
2.01
2.19
1.99
2.32
3.01
1.92
1.00
0.97
1.05
8.44
2.84
5.55
5.61
2.57
f1 (ppm)

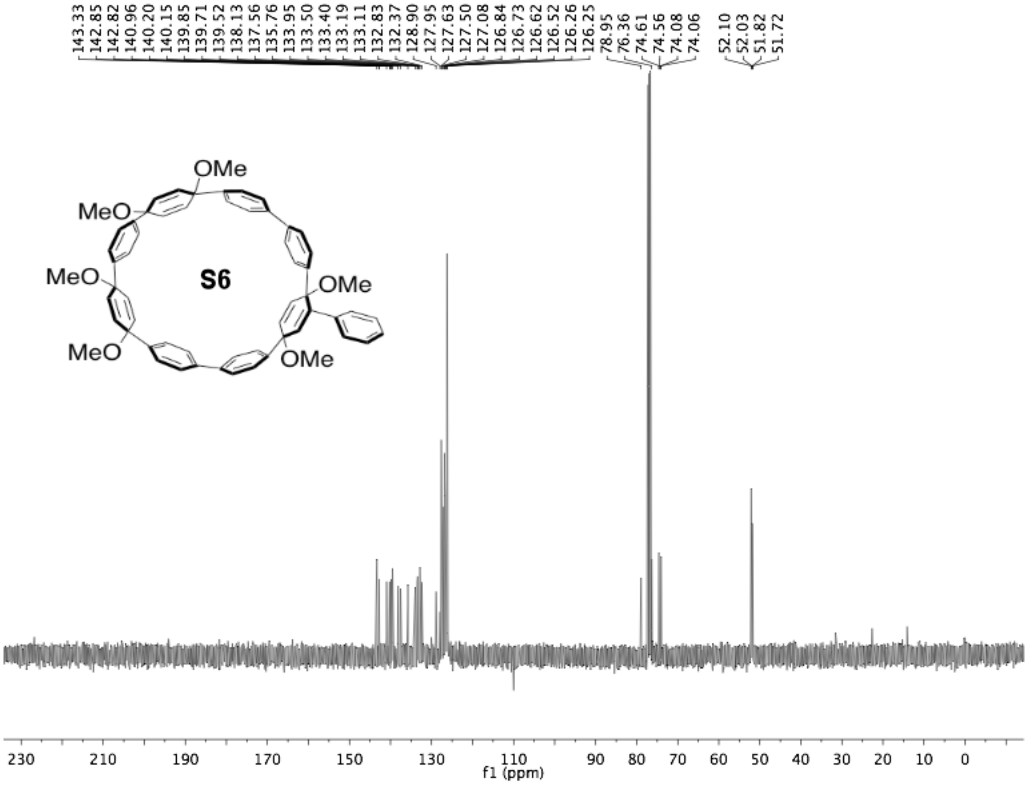
143.33
142.85
142.82
140.96
140.20
140.15
139.85
139.71
139.52
138.13
137.56
135.76
133.95
133.50
133.40
133.19
133.11
132.83
132.37
128.90
127.95
127.63
127.50
127.08
126.84
126.73
126.62
126.52
126.26
126.25
78.95
76.36
74.61
74.56
74.08
74.06
52.10
52.03
51.82
51.72
OMe
MeO
MeO
MeO
S6
OMe
OMe
f1 (ppm)

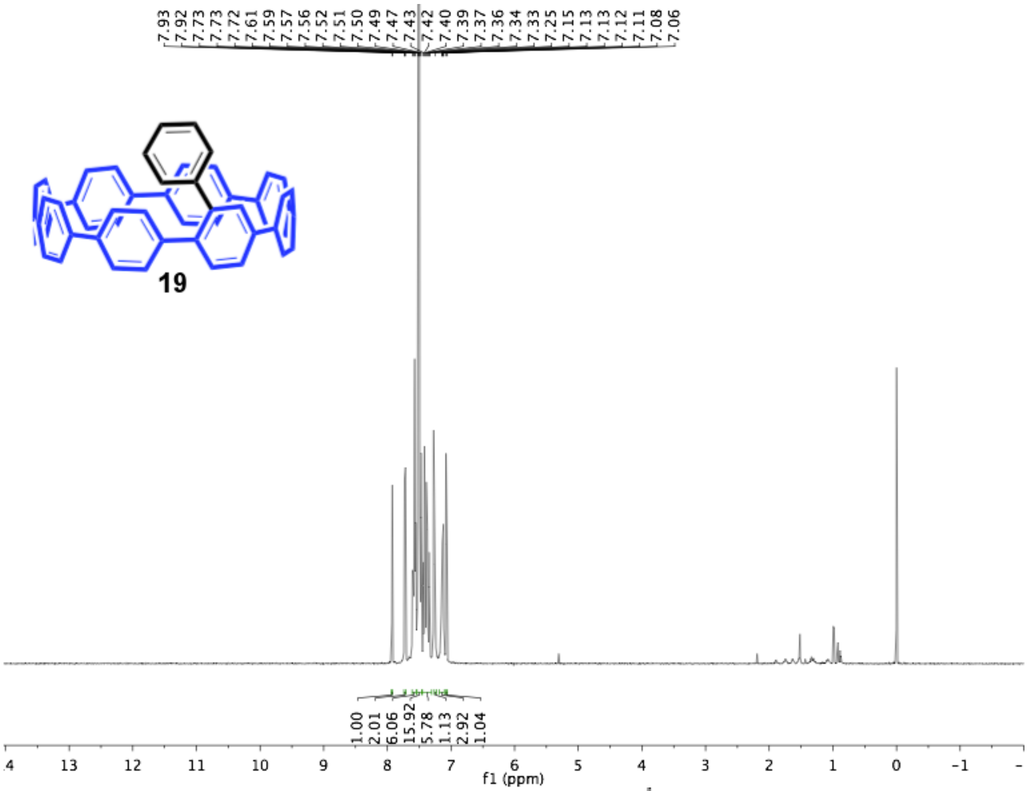
7.93
7.92
7.73
7.73
7.72
7.61
7.59
7.57
7.56
7.52
7.51
7.50
7.49
7.47
7.43
7.42
7.40
7.39
7.37
7.36
7.34
7.33
7.25
7.15
7.13
7.13
7.12
7.11
7.08
7.06
19
1.00
2.01
6.06
15.92
5.78
1.13
2.92
1.04
f1 (ppm)

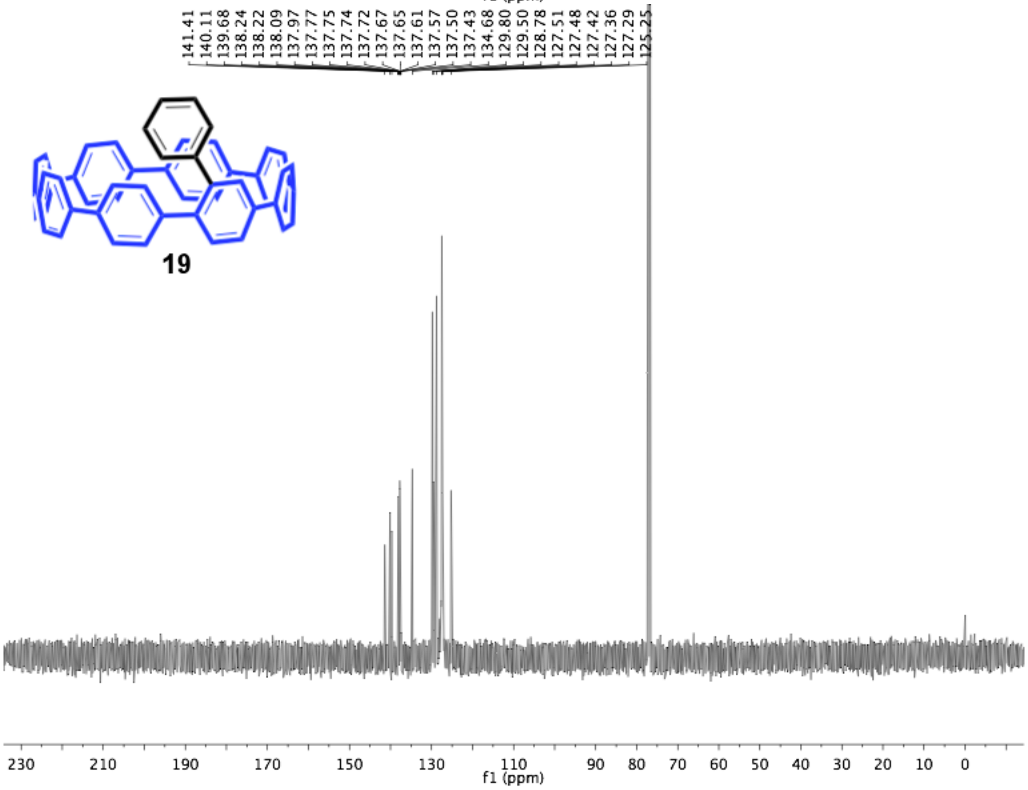
141.41
140.11
139.68
138.24
138.22
138.09
137.97
137.77
137.75
137.74
137.72
137.67
137.65
137.61
137.57
137.50
137.43
134.68
129.80
129.50
128.78
127.51
127.48
127.42
127.36
127.29
125.25
19
f1 (ppm)

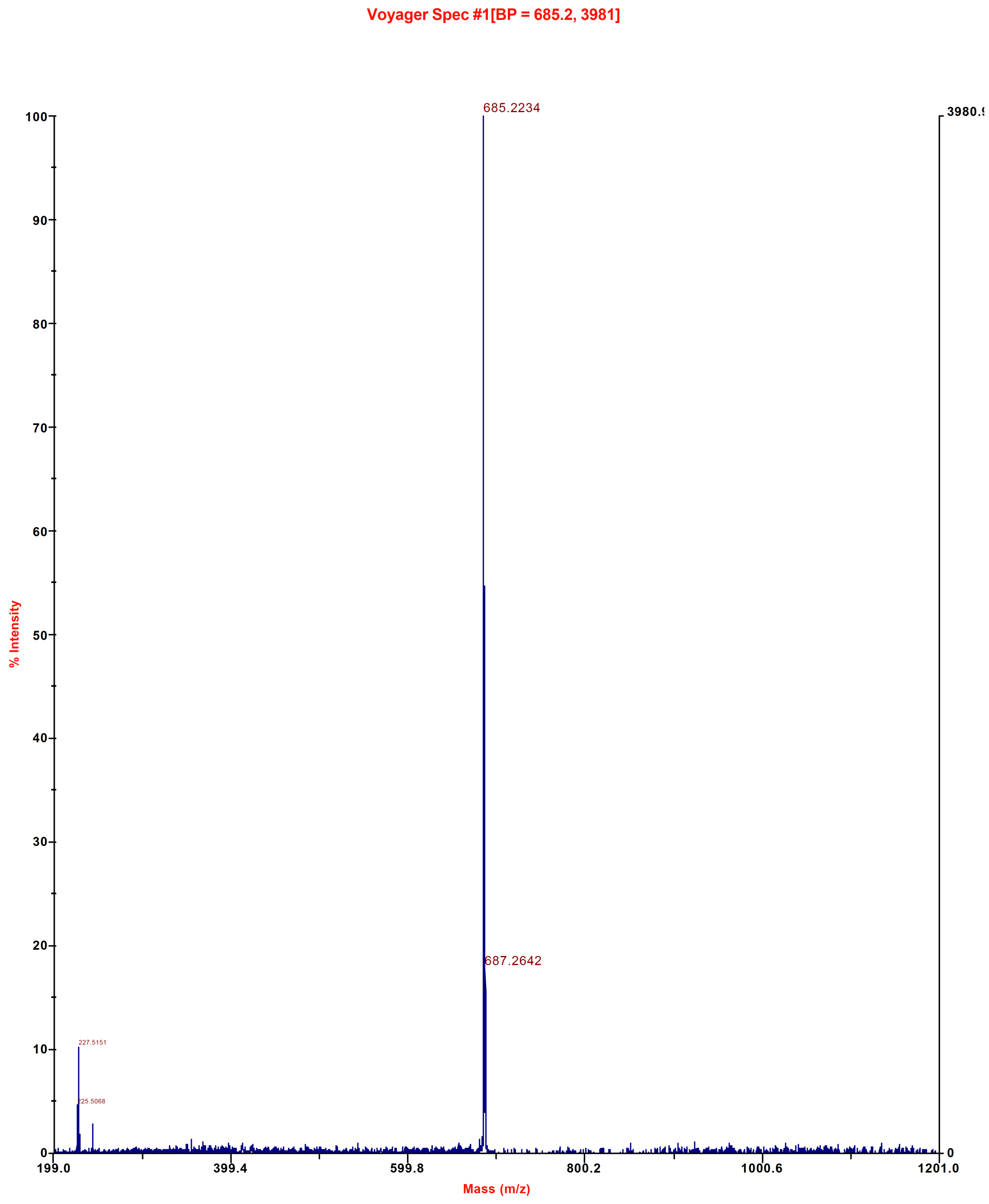

C:\...\MonoAryl CPP_0001.dat
Acquired: 19:06:00, January 29, 2014

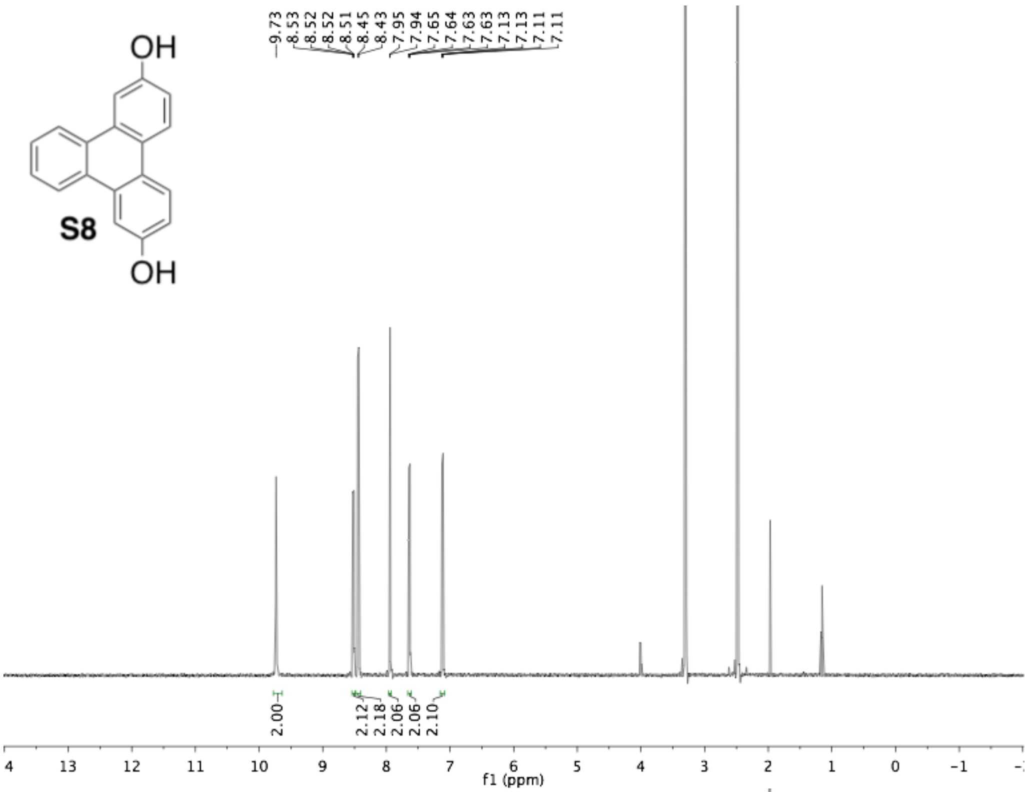
OH
S8
OH
9.73
8.53
8.52
8.52
8.51
8.45
8.43
7.95
7.94
7.65
7.64
7.63
7.63
7.13
7.13
7.11
7.11
2.00
2.12
2.18
2.06
2.06
2.10
f1 (ppm)

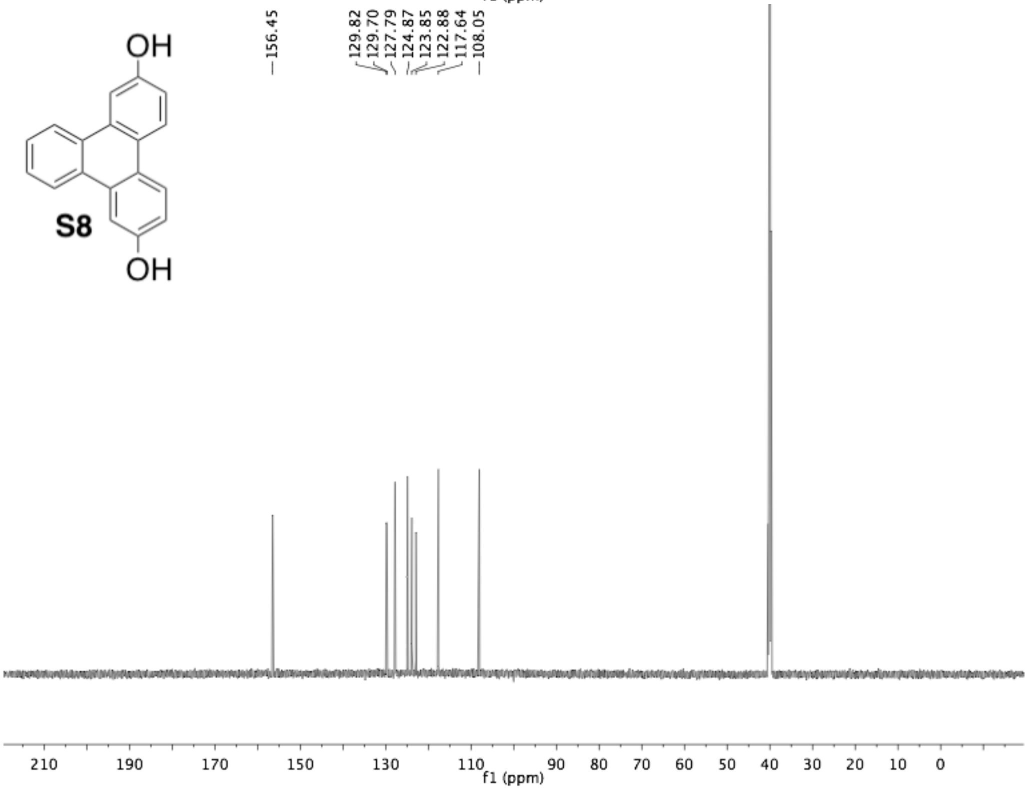
OH
S8
OH
156.45
129.82
129.70
127.79
124.87
123.85
122.88
117.64
108.05
f1 (ppm)

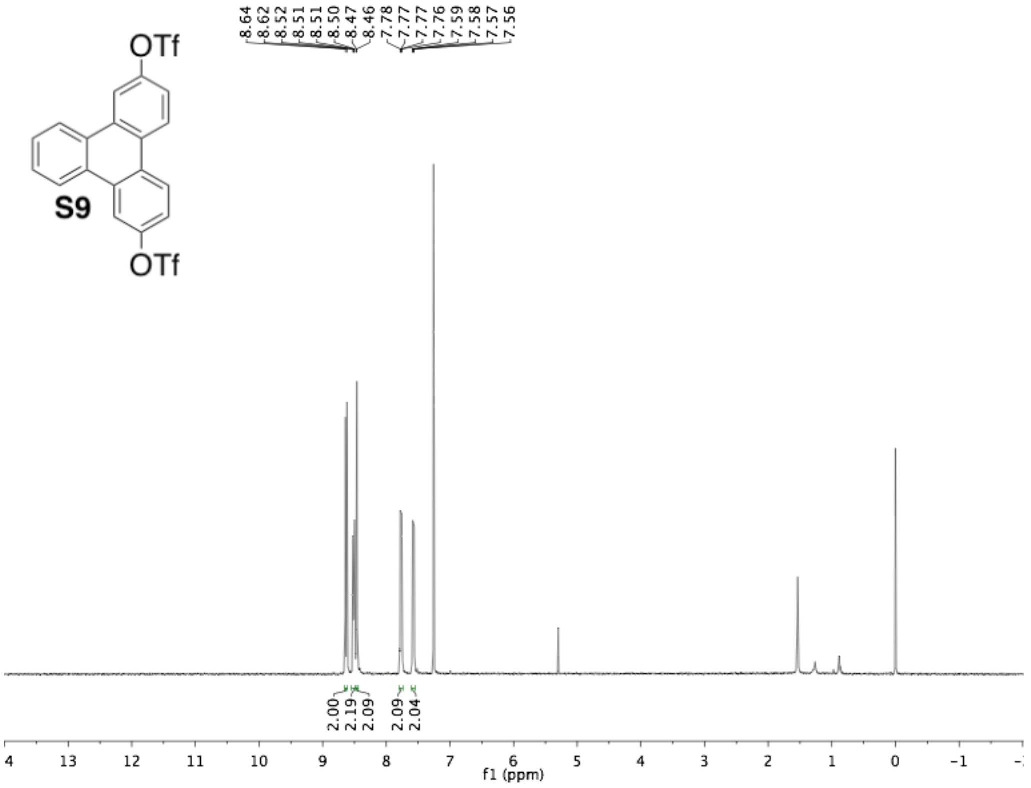
OTf
S9
OTf
8.64
8.62
8.52
8.51
8.51
8.50
8.47
8.46
7.78
7.77
7.77
7.76
7.59
7.58
7.57
7.56
2.00
2.19
2.09
2.09
2.04
f1 (ppm)

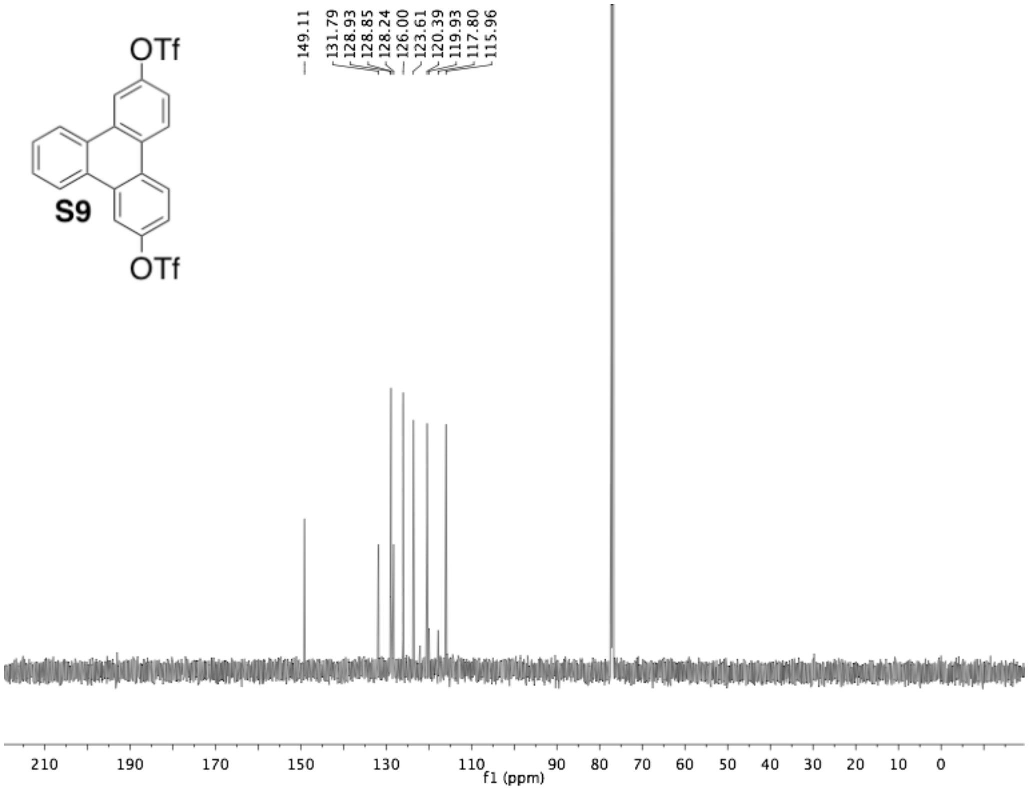
OTf
S9
OTf
149.11
131.79
128.93
128.85
128.24
126.00
123.61
120.39
119.93
117.80
115.96
f1 (ppm)

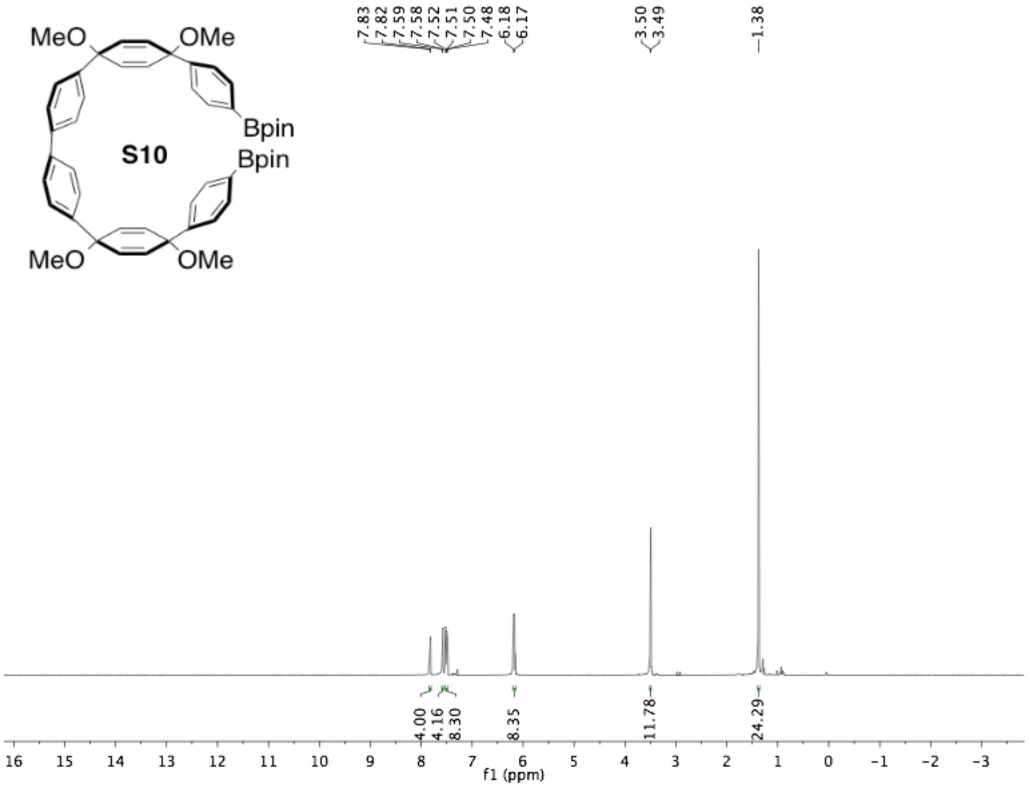
MeO
OMe
Bpin
S10
Bpin
MeO
OMe
7.83
7.82
7.59
7.58
7.52
7.51
7.50
7.48
6.18
6.17
3.50
3.49
1.38
4.00
4.16
8.30
8.35
11.78
24.29
f1 (ppm)

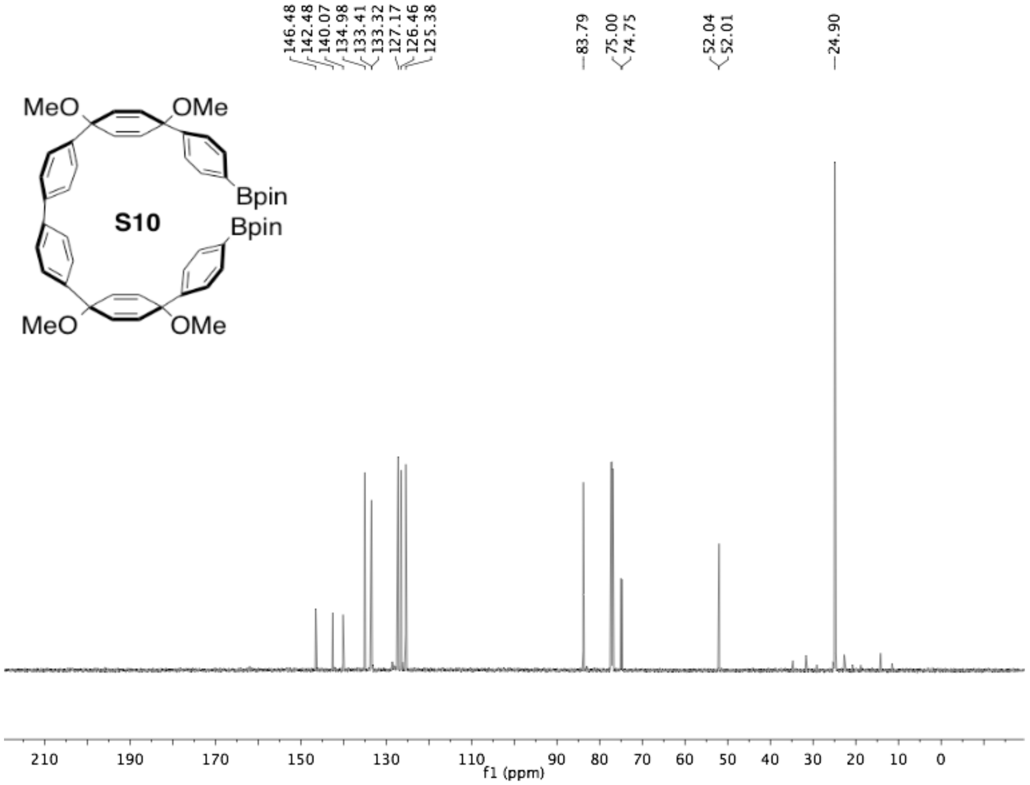
MeO
OMe
Bpin
S10
Bpin
MeO
OMe
146.48
142.48
140.07
134.98
133.41
133.32
127.17
126.46
125.38
83.79
75.00
74.75
52.04
52.01
24.90
f1 (ppm)

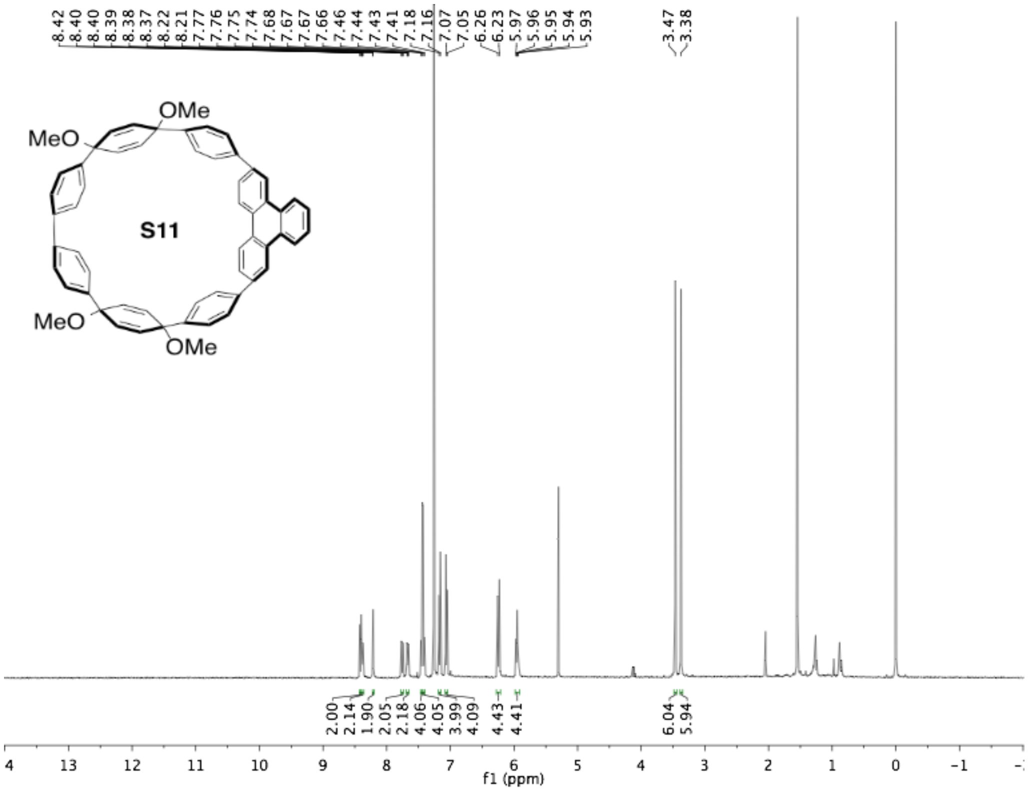
8.42
8.40
8.40
8.39
8.38
8.37
8.22
8.21
7.77
7.76
7.75
7.74
7.68
7.67
7.66
7.46
7.44
7.43
7.41
7.18
7.16
7.07
7.05
6.26
6.23
5.97
5.96
5.95
5.94
5.93
3.47
3.38
OMe
MeO
S11
MeO
OMe
2.00
2.14
1.90
2.05
2.18
4.06
4.05
3.99
4.09
4.43
4.41
6.04
5.94
f1 (ppm)

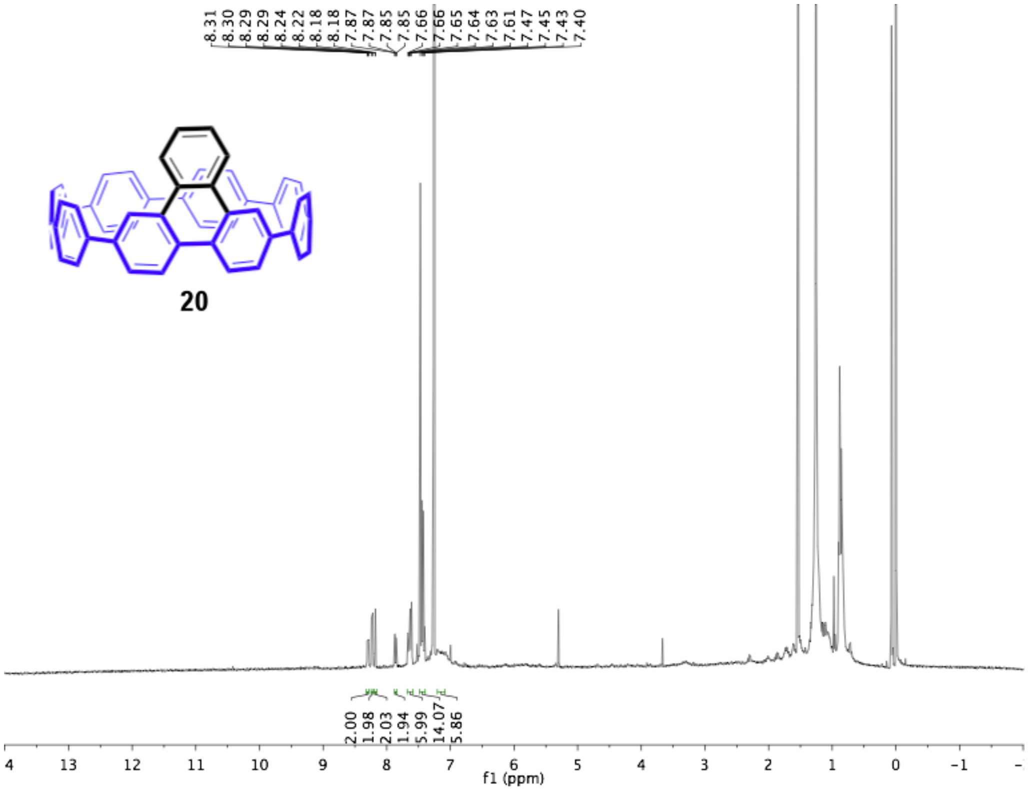
8.31
8.30
8.29
8.29
8.24
8.22
8.18
8.18
7.87
7.87
7.85
7.85
7.66
7.66
7.65
7.64
7.63
7.61
7.47
7.45
7.43
7.40
20
2.00
1.98
2.03
1.94
5.99
14.07
5.86
f1 (ppm)

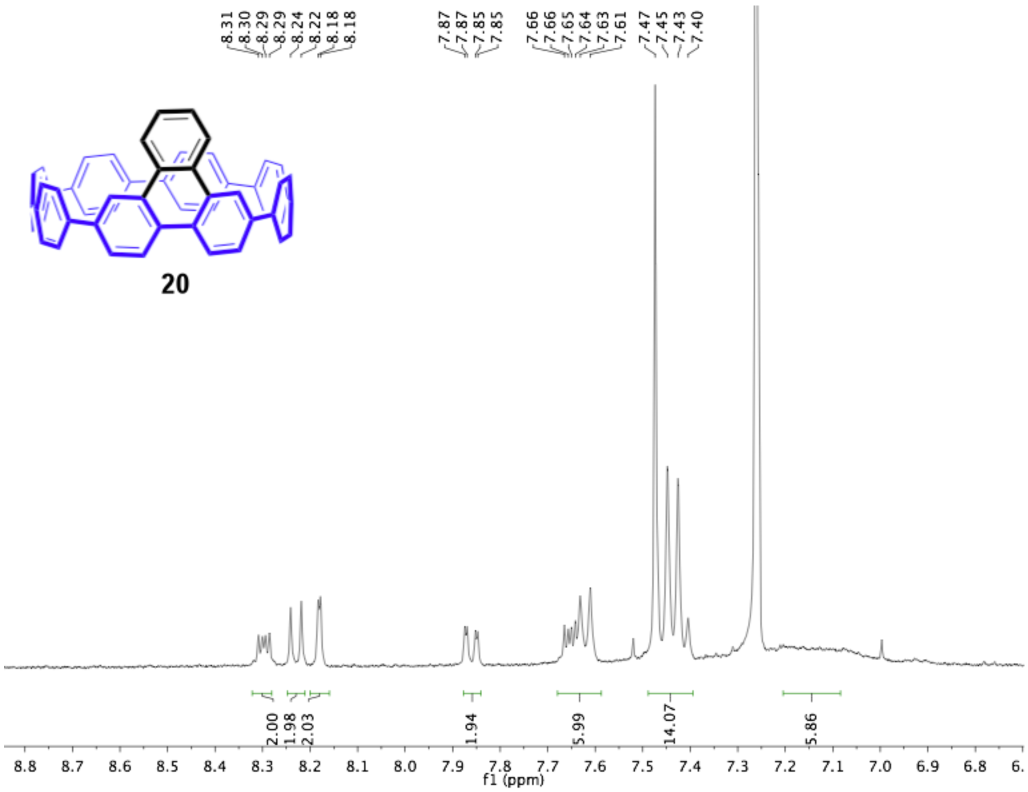
8.31
8.30
8.29
8.29
8.24
8.22
8.18
8.18
7.87
7.87
7.85
7.85
7.66
7.66
7.65
7.64
7.63
7.61
7.47
7.45
7.43
7.40
20
2.00
1.98
2.03
1.94
5.99
14.07
5.86
8.8 8.7 8.6 8.5 8.4 8.3 8.2 8.1 8.0 7.9 7.8 7.7 7.6 7.5 7.4 7.3 7.2 7.1 7.0 6.9 6.8 6.
f1 (ppm)

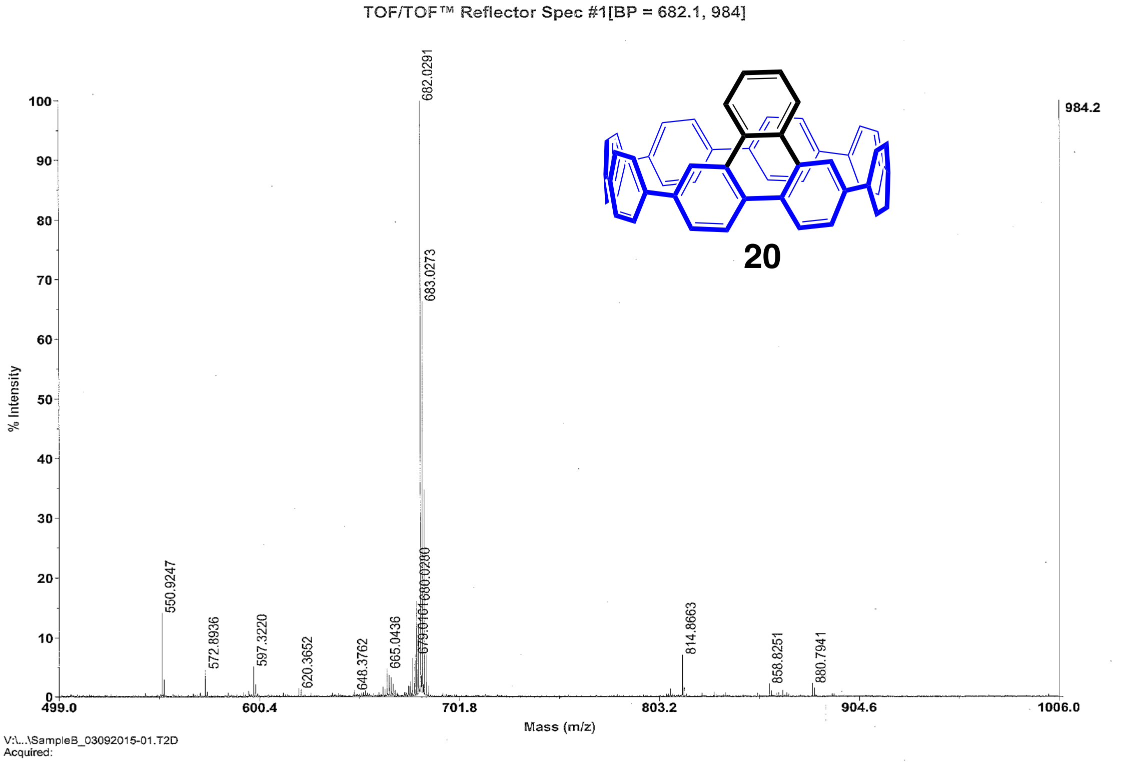
TOF/TOF™ Reflector Spec #1[BP = 682.1, 984]
% Intensity
100
90
80
70
60
50
40
30
20
10
0
682.0291
683.0273
550.9247
572.8936
597.3220
620.3652
648.3762
665.0436
679.0161
680.0280
814.8663
858.8251
880.7941
984.2
20
499.0
600.4
701.8
803.2
904.6
1006.0
Mass (m/z)
V:\...\SampleB_03092015-01.T2D
Acquired:

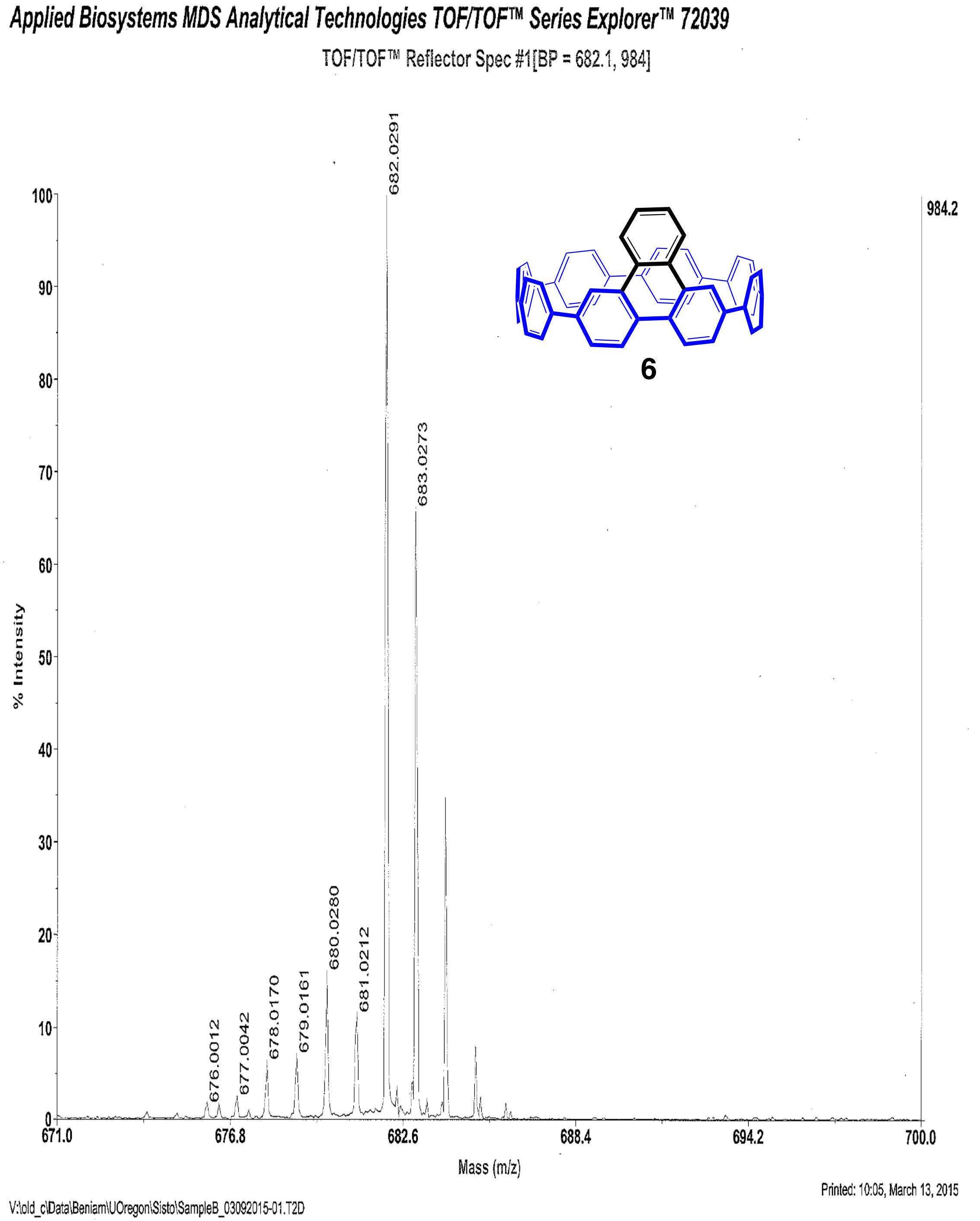

Applied Biosystems MDS Analytical Technologies TOF/TOF™ Series Explorer™ 72039
TOF/TOF™ Reflector Spec #1[BP = 682.1, 984]
682.0291
683.0273
680.0280
681.0212
679.0161
678.0170
677.0042
676.0012
984.2
6
% Intensity
100
90
80
70
60
50
40
30
20
10
0
671.0
676.8
682.6
688.4
694.2
700.0
Mass (m/z)
Printed: 10:05, March 13, 2015
V:\old_c\Data\Beniam\UOregon\Sisto\SampleB_03092015-01.T2D

Triflic Acid Scholl Reaction on Mono-aryl [8]CPP (19): A 2 mL oven dried vial with cap was charged with a dry stirbar, mono-aryl substituted [8]CPP **5** (10 mg, 0.0146 mmol, 1 eq.), and dichloromethane (0.110 mL, sparged with O_2 seperately). Trifluoromethanesulfonic acid (0.002 mL, or one drop, 0.0292, 2 eq.) was added and the reaction was heated to reflux after being sealed. The reaction was monitored by thin layer chromatography, and the reaction was stirred for one hour. At one hour, the reaction was quenched by addition of sodium bicarbonate. The reaction was diluted with dichloromethane (5 mL), and the aqueous and organic layers were separated. The aqueous layer was extracted with dichloromethane (3 X 3 mL), washed with brine, and dried over sodium sulfate. The solvent was removed under reduced pressure to yield a crude solid, which was purified by preparatory thin layer chromatography (5:95 ethyl acetate:dichloromethane) to afford a single band (4 mg). Characterization included ^{1}H NMR (crude and purified material), and MALDI-TOF MS.

Triflic Acid Scholl Reaction on [8]CPP (22): A 2 mL oven dried vial with cap was purged with argon after being charged with a dry stirbar, [8]CPP (**7**) (8.5 mg, 0.014 mmol, 1 eq.), and dichloromethane (0.5 mL, sparged with argon to quell dimerization/oligomerization). Trifluoromethanesulfonic acid (0.006 mL, or three drops, 0.0876, 6 eq.) was added and the reaction was heated to reflux after being sealed. The reaction was monitored by thin layer chromatography, and the reaction was stirred for one hour. At one hour, the reaction was quenched by addition of sodium bicarbonate. The reaction was diluted with dichloromethane (5 mL), and the aqueous and organic layers were separated. The aqueous layer was extracted with dichloromethane (3 X 3 mL),

washed with brine, and dried over sodium sulfate. The solvent was removed under reduced pressure to yield a crude solid (4.5 mg), which was purified by recycling GPC. The purification was conducted by recycling the material 3 times, at which point fractions were collected due to overlap (the peak with the shortest R_t began to overlap the peaks with the longest R_t). These collected fractions were then injected again (separate runs for each previously collected fraction) as the mixture contained compounds with very different R_t. The amounts ultimately collected from the GPC were less than 1 mg. Characterization included ^{1}H NMR, MALDI-TOF MS, and single crystal X-Ray diffraction.

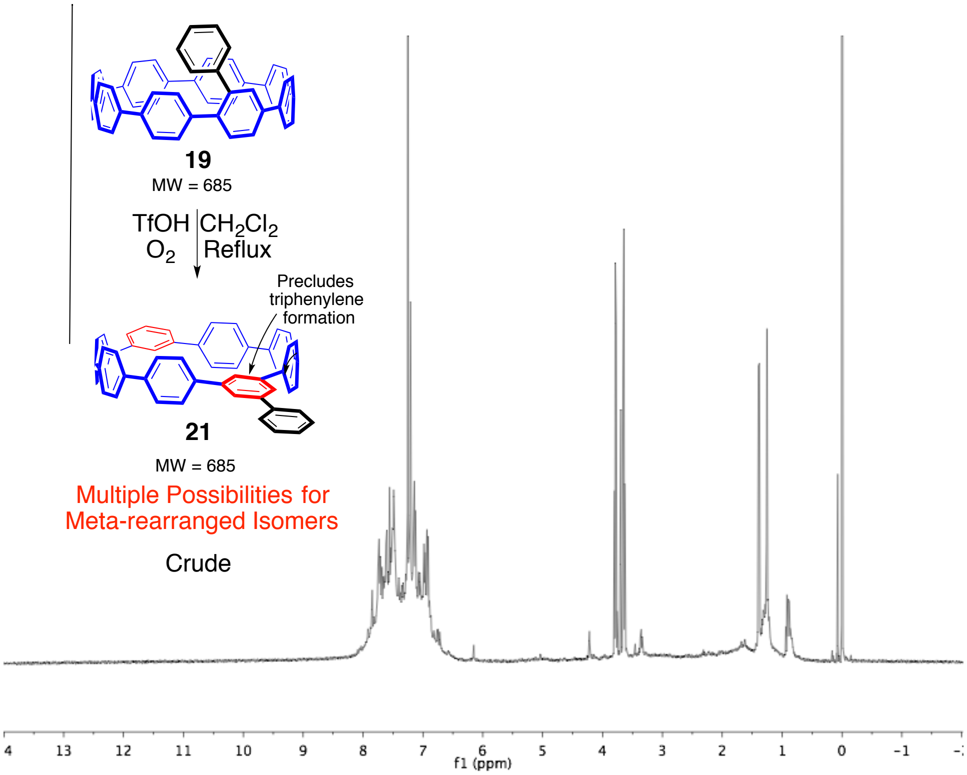
19
MW = 685
TfOH
O_2
CH_2Cl_2
Reflux
Precludes
triphenylene
formation
21
MW = 685
Multiple Possibilities for
Meta-rearranged Isomers
Crude
f1 (ppm)

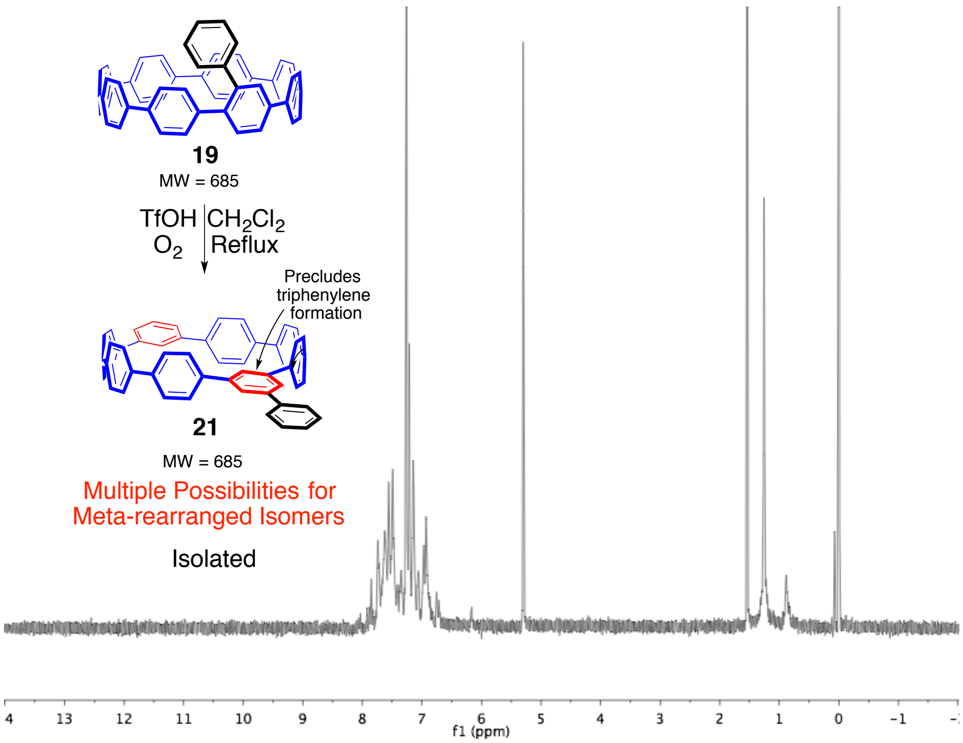
19
MW = 685
TfOH
O_2
CH_2Cl_2
Reflux
Precludes
triphenylene
formation
21
MW = 685
Multiple Possibilities for
Meta-rearranged Isomers
Isolated
f1 (ppm)

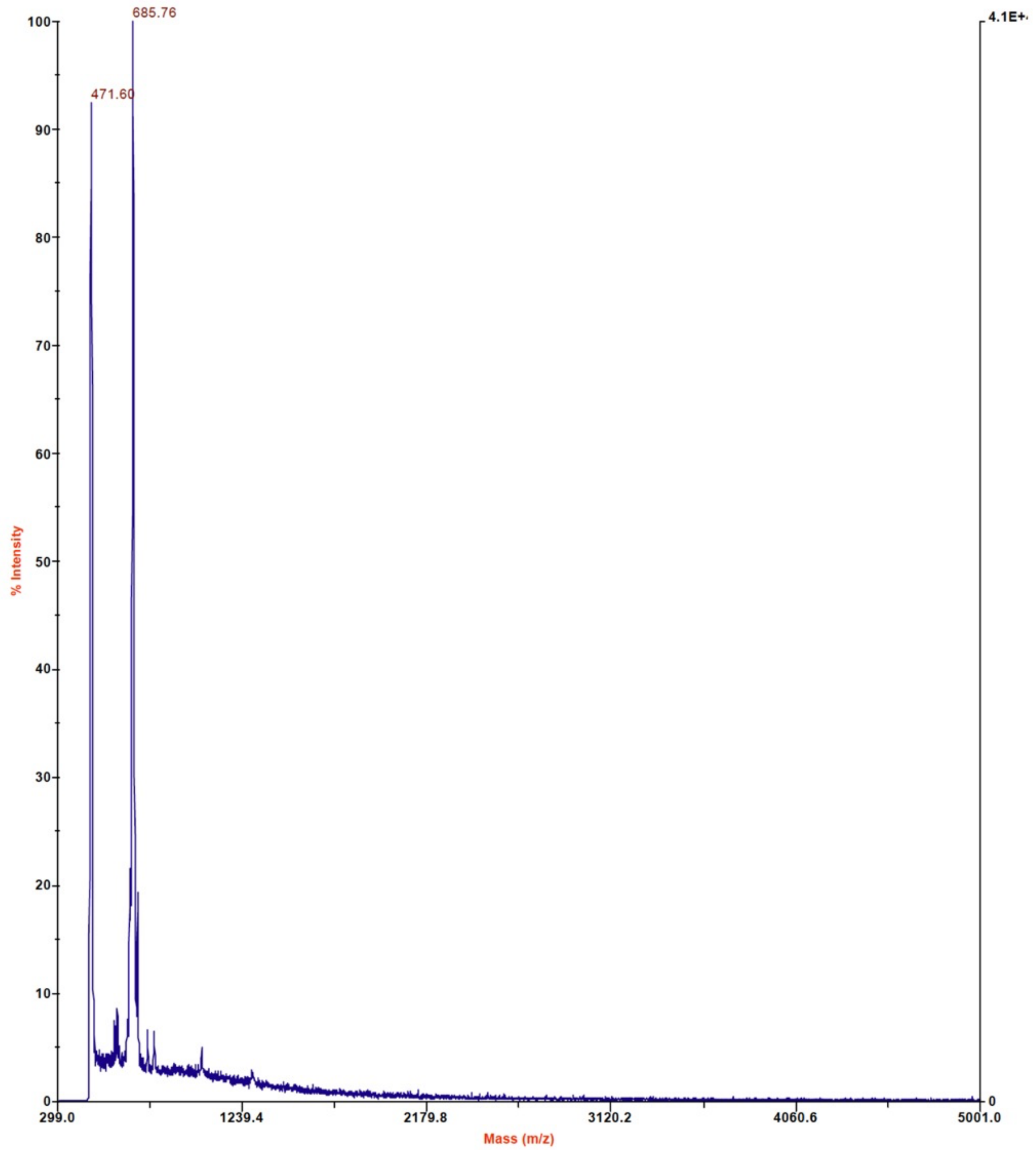

The MALDI-TOF spectra obtained of the purified material from the attempted Scholl reaction of **19** with triflic acid. The peak at 471.60 is potentially attributable to the dimer with 2 sodium adducts and 3^+ charge $(2M+2Na)^{+3}$.

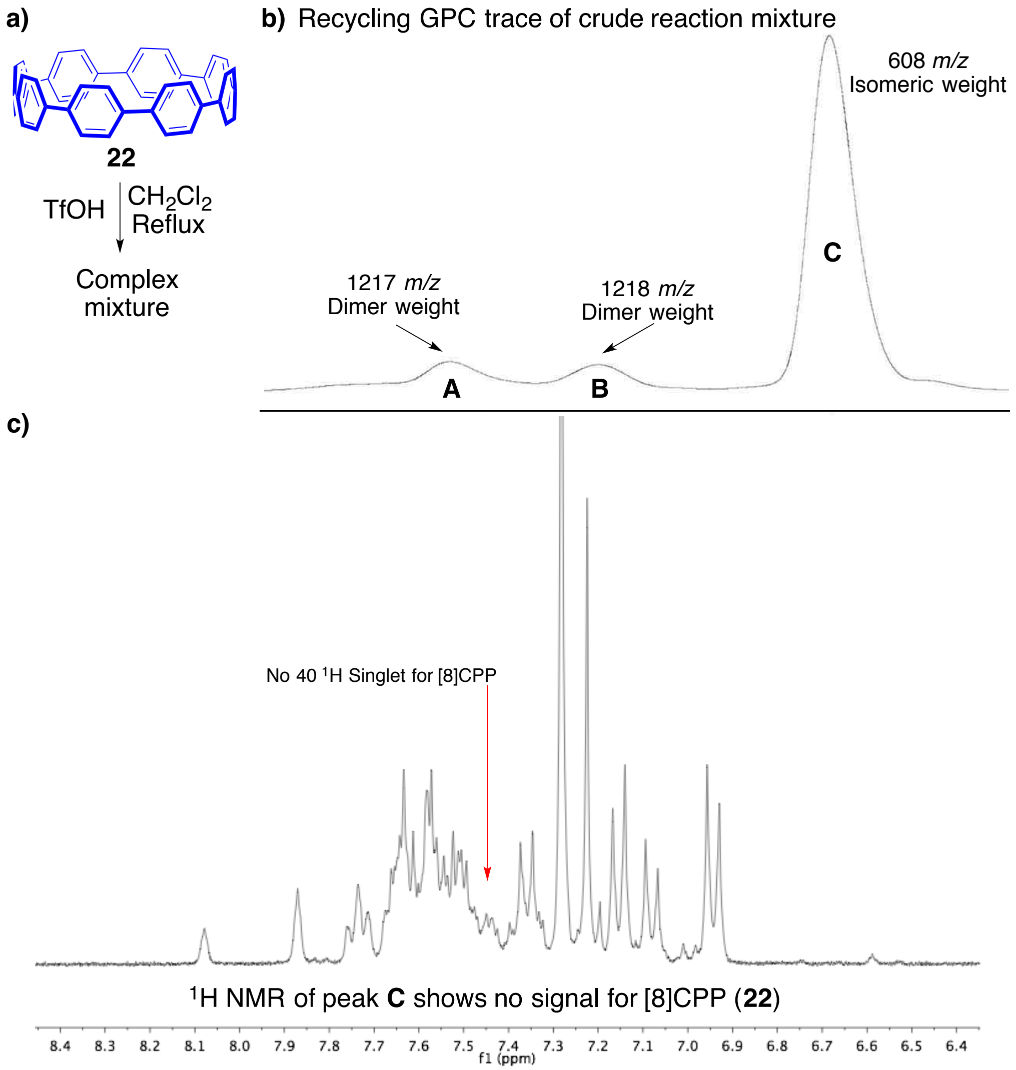

Figure 6. a) Reaction conditions to investigate the potential cationic rearrangement of [8]CPP. b) The recycling GPC trace from the first of four cycles of the crude reaction mixture, along with the corresponding MALDI-TOF masses. c) The ^{1}H NMR of peak C collected from the recycling GPC. The spectra shows a complex mixture of compounds and no [8]CPP, while MALDI-TOF displays simply the isomeric 608.1 m/z.

Figure 6 from the article. Raw data is presented in the following pages.

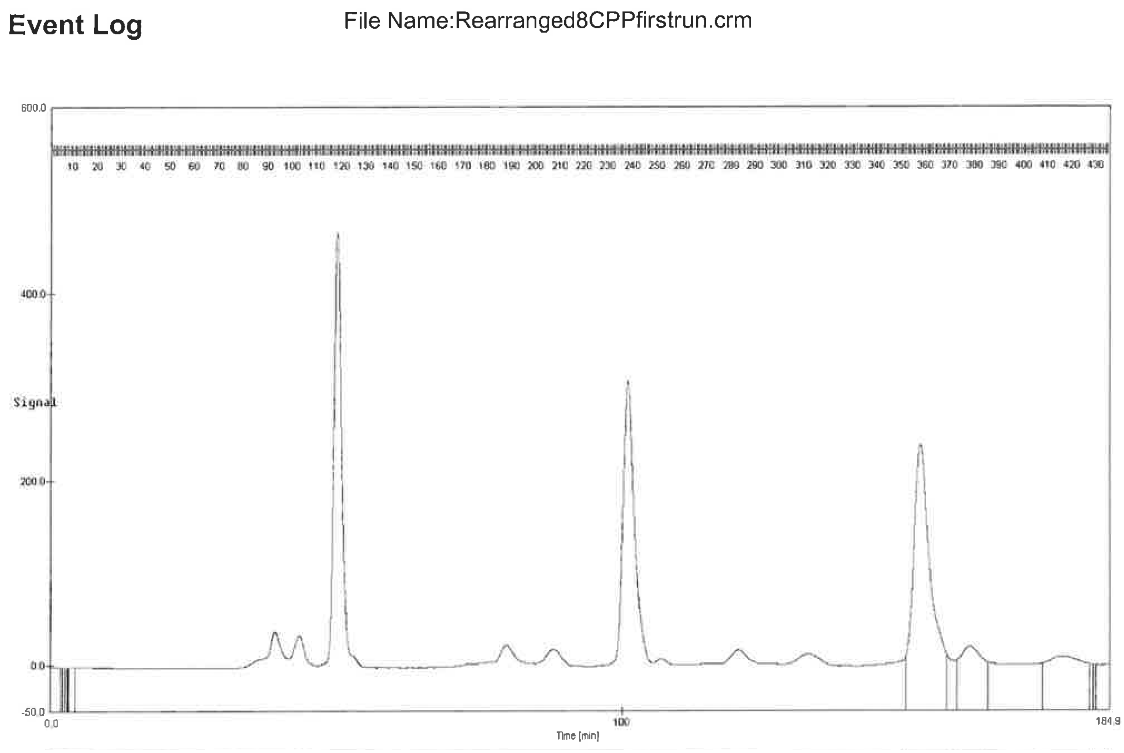

Initial injection of the crude material from the exposure of [8]CPP (**22**) to triflic acid Scholl conditions. The material was recycled 3 times before collection and subsequently each fraction was put through a second, individual injection.

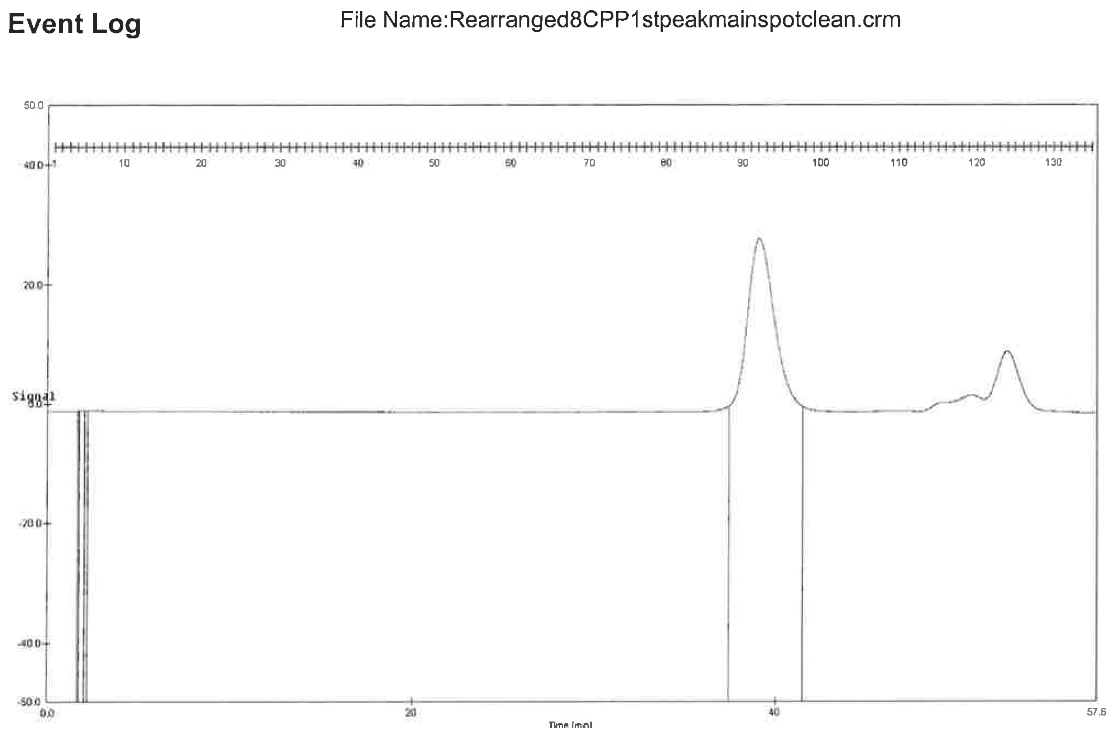

The final collection of peak **A**.

Event Log File Name:Rearranged8CPP2ndpeakmainspotclean.crm

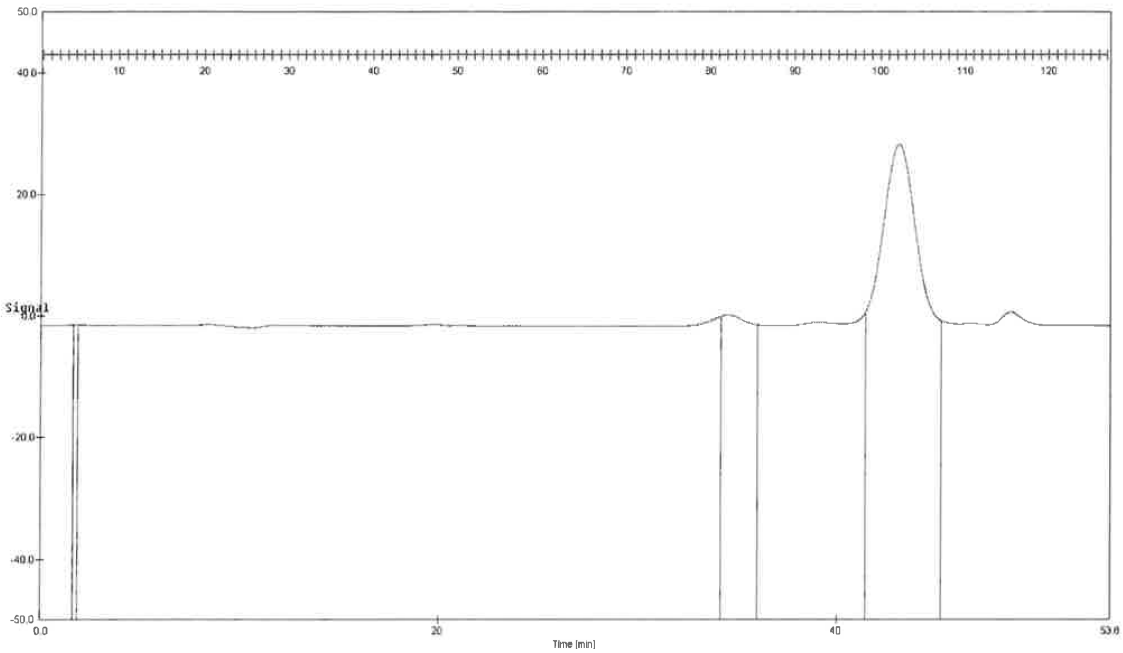

Final collection of peak **B**.

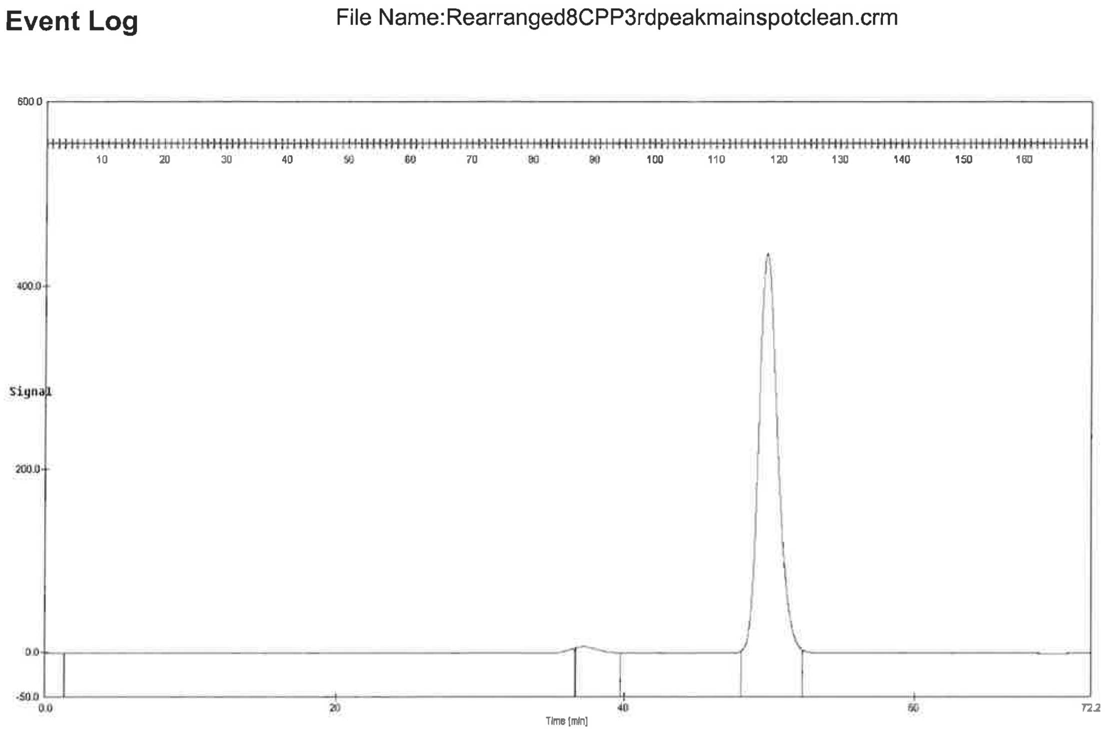

Final collection of peak **C.**

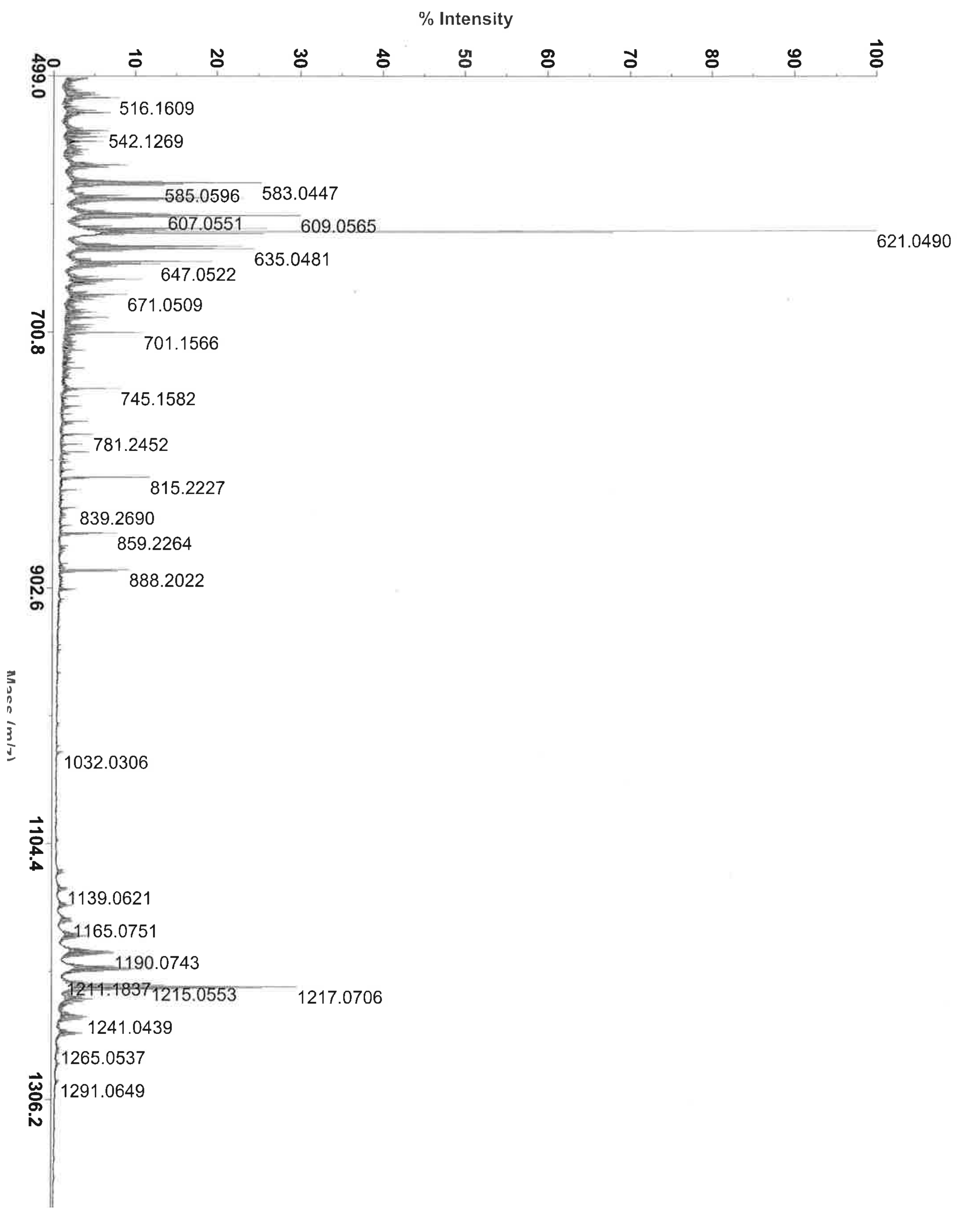

MALDI-TOF spectrum of GPC peak **A**.

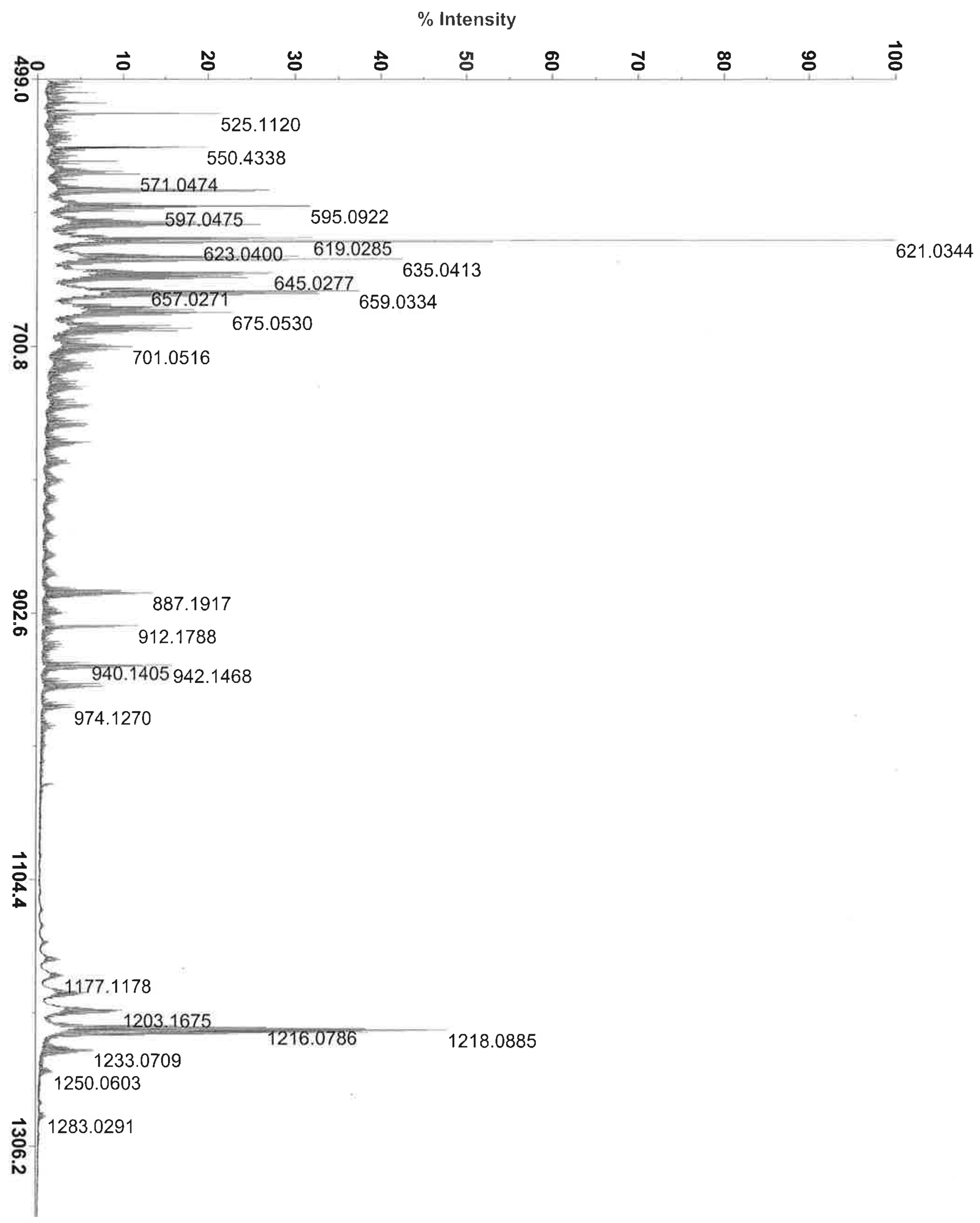

MALDI-TOF spectrum of GPC peak **B**.

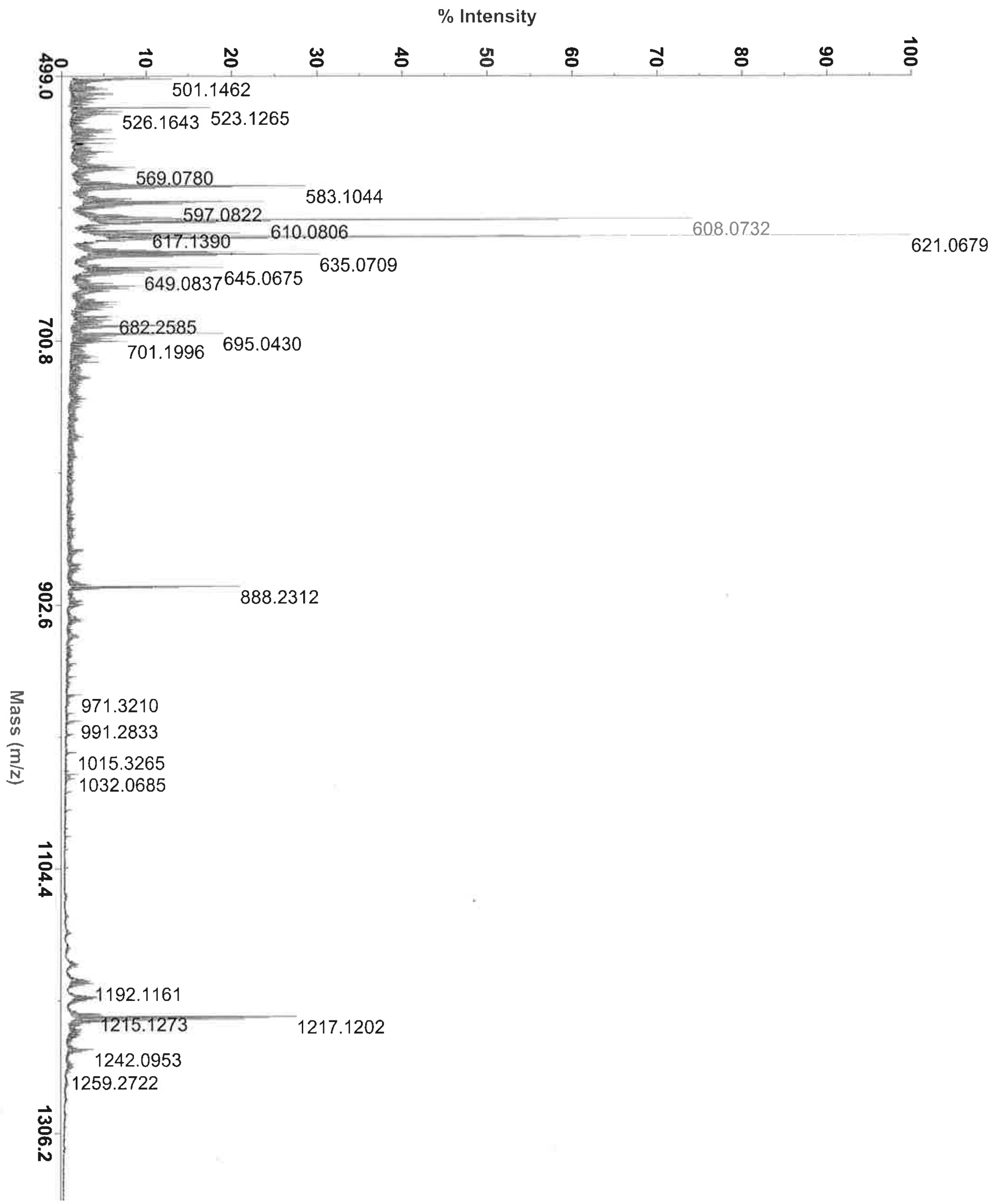

MALDI-TOF spectrum of GPC peak **C**.

Computational Data: Calculations performed on [8]CPP (**22-22e**), and compound **23** were conducted with B3LYP/6-31+(d,p), while the calculations conducted on highly aryl-substituted [8]CPP (**27-27e**) were performed with B3LYP/6-31(d,p) due to the high computational cost of these larger molecules. Radicals **25** and **26** (Nu = H) were minimized at the B3LYP/6-31(d) level of theory.

A value of 962.034875 Hartree/Particle was used for TfOH as reported in the literature[7].

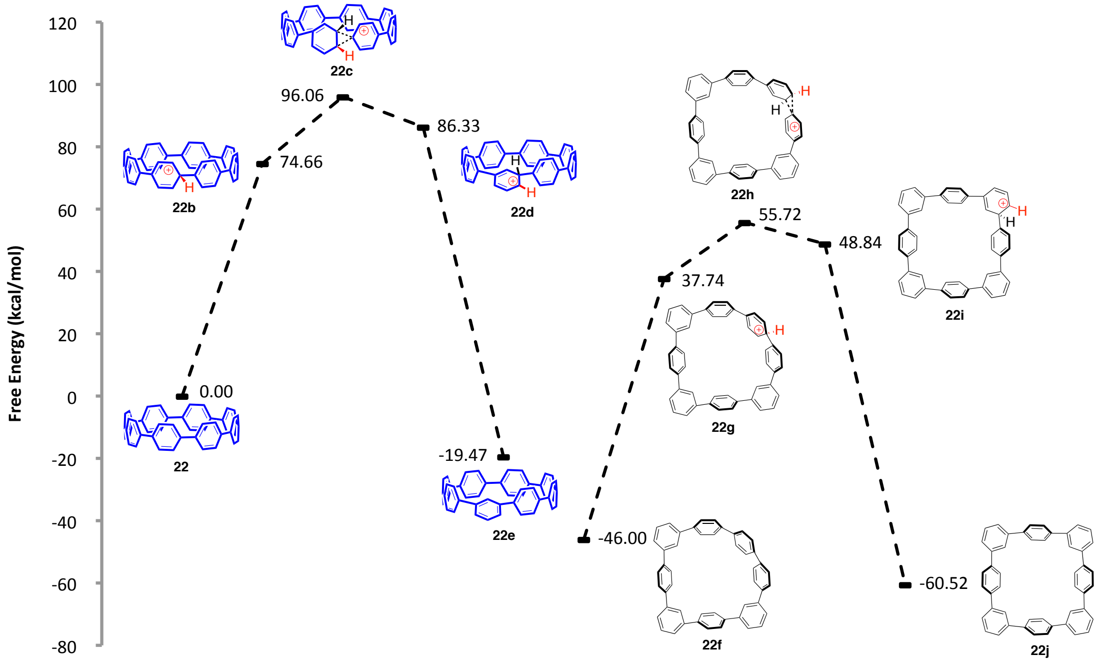

Figure 8. Calculated reaction coordinate for the *ipso* protonation and subsequent meta-rearrangement of [8]CPP (22).

7 Ajaz, A.; McLaughlin, E. C.; Skraba, S. L.; Thamatam, R.; Johnson, R. P. *J. Org. Chem.* **2012**, *77*, 9487-9495

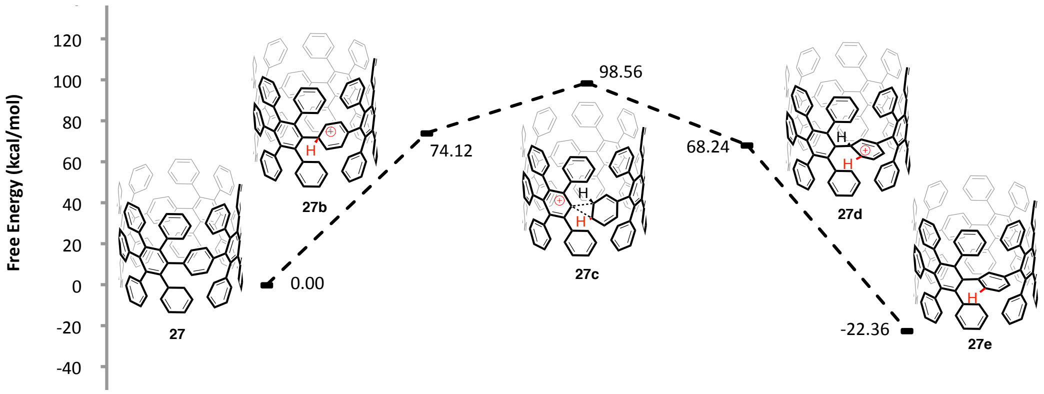

Figure 10. Calculated reaction coordinate for the *ipso* protonation and subsequent meta-rearrangement of fully aryl-substituted [8]CPP (27)

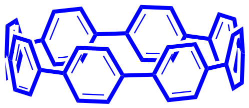

22

Charge = 0 Multiplicity = 1

H	3.55858	5.28597	1.87681
C	3.6996	4.69415	0.97448
C	4.05048	3.062	-1.24256
C	2.78719	4.79961	-0.08682
C	4.69415	3.6996	0.97448
C	4.79961	2.78719	-0.08682
C	3.062	4.05048	-1.24256
H	5.28597	3.55858	1.87681
H	2.39583	4.09289	-2.10228
H	4.09289	2.39583	-2.10228
C	1.423	5.36468	0.08682
C	-1.423	5.36468	0.08682
C	0.69896	5.02929	1.24256
C	0.70326	5.93528	-0.97448
C	-0.70326	5.93528	-0.97448
C	-0.69896	5.02929	1.24256
H	1.2	4.58822	2.10228
H	1.22145	6.25404	-1.87681
H	-1.22145	6.25404	-1.87681
H	-1.2	4.58822	2.10228
C	-2.78719	4.79961	-0.08682
C	-4.79961	2.78719	-0.08682
C	-3.062	4.05048	-1.24256
C	-3.6996	4.69415	0.97448

C	-4.69415	3.6996	0.97448
C	-4.05048	3.062	-1.24256
H	-2.39583	4.09289	-2.10228
H	-3.55858	5.28597	1.87681
H	-5.28597	3.55858	1.87681
H	-4.09289	2.39583	-2.10228
C	-5.36468	1.423	0.08682
C	-5.36468	-1.423	0.08682
C	-5.02929	0.69896	1.24256
C	-5.93528	0.70326	-0.97448
C	-5.93528	-0.70326	-0.97448
C	-5.02929	-0.69896	1.24256
H	-4.58822	1.2	2.10228
H	-6.25404	1.22145	-1.87681
H	-6.25404	-1.22145	-1.87681
H	-4.58822	-1.2	2.10228
C	-4.05048	-3.062	-1.24256
C	-3.6996	-4.69415	0.97448
C	-4.79961	-2.78719	-0.08682
C	-3.062	-4.05048	-1.24256
C	-2.78719	-4.79961	-0.08682
C	-4.69415	-3.6996	0.97448
H	-2.39583	-4.09289	-2.10228
H	-5.28597	-3.55858	1.87681
H	-3.55858	-5.28597	1.87681
C	5.36468	1.423	0.08682
C	5.36468	-1.423	0.08682
C	5.93528	0.70326	-0.97448
C	5.02929	0.69896	1.24256
C	5.02929	-0.69896	1.24256
C	5.93528	-0.70326	-0.97448
H	6.25404	1.22145	-1.87681
H	4.58822	1.2	2.10228
H	4.58822	-1.2	2.10228
H	6.25404	-1.22145	-1.87681
C	4.79961	-2.78719	-0.08682
C	2.78719	-4.79961	-0.08682
C	4.05048	-3.062	-1.24256
C	4.69415	-3.6996	0.97448
C	3.6996	-4.69415	0.97448
C	3.062	-4.05048	-1.24256
H	4.09289	-2.39583	-2.10228
H	5.28597	-3.55858	1.87681
H	3.55858	-5.28597	1.87681

H	2.39583	-4.09289	-2.10228
C	1.423	-5.36468	0.08682
C	-1.423	-5.36468	0.08682
C	0.70326	-5.93528	-0.97448
C	0.69896	-5.02929	1.24256
C	-0.69896	-5.02929	1.24256
C	-0.70326	-5.93528	-0.97448
H	1.22145	-6.25404	-1.87681
H	1.2	-4.58822	2.10228
H	-1.2	-4.58822	2.10228
H	-1.22145	-6.25404	-1.87681
H	-4.09289	-2.39583	-2.10228

Zero-point correction= 0.643127 (Hartree/Particle)
Thermal correction to Energy= 0.677934
Thermal correction to Enthalpy= 0.678878
Thermal correction to Gibbs Free Energy= 0.579414
Sum of electronic and zero-point Energies= -1847.800681
Sum of electronic and thermal Energies= -1847.765874
Sum of electronic and thermal Enthalpies= -1847.764929
Sum of electronic and thermal Free Energies= -1847.864394

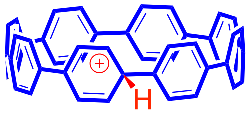

22b

Charge = 1 Multiplicity = 1

H	4.613	1.10108	-1.89312
C	4.57024	1.86366	-1.12342
C	4.36642	3.81879	0.84848
C	5.52753	1.90161	-0.10337
C	3.51572	2.7744	-1.14194
C	3.35643	3.7331	-0.12742
C	5.43744	2.92337	0.85581
H	2.75963	2.68833	-1.91613
H	6.19066	3.01081	1.63515
H	4.30576	4.57977	1.62101
C	6.58323	0.76475	0.06011
C	5.40116	-1.90254	0.09614
C	6.61616	-0.15719	-1.11591
C	6.2255	0.0356	1.32003
C	5.66617	-1.19993	1.3264

C	6.04354	-1.38442	-1.09616
H	7.07497	0.20914	-2.03058
H	5.32858	-1.61728	2.26693
H	6.0632	-1.99439	-1.99238
H	7.56937	1.23963	0.17608
C	2.05874	4.4519	-0.04074
C	-0.7315	5.00848	-0.00361
C	1.33375	4.42709	1.1625
C	1.40345	4.94679	-1.18362
C	0.04154	5.23518	-1.16079
C	-0.03384	4.69279	1.17834
H	1.8117	4.0734	2.07162
H	1.9559	5.06356	-2.11196
H	-0.43688	5.57741	-2.07327
H	-0.58198	4.52792	2.10001
C	4.45567	-2.97487	0.04984
C	2.02914	-4.50412	0.00647
C	3.83306	-3.35472	-1.17766
C	3.92849	-3.57848	1.23365
C	2.77754	-4.32992	1.20729
C	2.66353	-4.08396	-1.1956
H	4.2146	-2.97797	-2.11941
H	4.43266	-3.44367	2.18372
H	2.41721	-4.75818	2.13517
H	2.17353	-4.24402	-2.1485
C	-2.20005	4.78861	-0.06514
C	-4.6546	3.34587	0.00402
C	-2.75309	4.2348	-1.23507
C	-3.02831	4.81544	1.07656
C	-4.22806	4.10908	1.11125
C	-3.95204	3.53207	-1.20229
H	-2.17504	4.21956	-2.15276
H	-2.69879	5.3316	1.9735
H	-4.79837	4.08926	2.03548
H	-4.2579	2.99472	-2.09362
C	-5.50207	2.13486	0.13558
C	-5.95109	-0.67087	0.11364
C	-6.10481	1.50489	-0.97632
C	-5.41457	1.37761	1.32003
C	-5.63469	0.00507	1.30993
C	-6.31766	0.131	-0.99012
H	-6.32004	2.07906	-1.87256
H	-5.00731	1.82764	2.21912
H	-5.39126	-0.55999	2.20345

H	-6.69379	-0.33282	-1.89744
C	0.62518	-4.90045	0.02366
C	-2.22878	-4.75059	-0.03303
C	-0.10554	-5.17582	-1.1619
C	-0.13253	-4.77899	1.21517
C	-1.51408	-4.70821	1.18712
C	-1.48784	-5.11676	-1.1859
H	0.41707	-5.40397	-2.08493
H	0.36307	-4.61375	2.16459
H	-2.03628	-4.48169	2.10947
H	-2.00212	-5.29493	-2.12437
C	-5.50551	-2.074	-0.03226
C	-3.57703	-4.16556	-0.10822
C	-4.91245	-2.47439	-1.2486
C	-5.3222	-2.9358	1.07492
C	-4.37432	-3.9485	1.04123
C	-3.97051	-3.4933	-1.286
H	-5.06409	-1.87973	-2.14268
H	-5.86514	-2.75382	1.99724
H	-4.20646	-4.53568	1.93894
H	-3.43018	-3.6553	-2.21227
H	6.33964	0.57389	2.25606

Zero-point correction= 0.656012 (Hartree/Particle)
Thermal correction to Energy= 0.691352
Thermal correction to Enthalpy= 0.692296
Thermal correction to Gibbs Free Energy= 0.589679
Sum of electronic and zero-point Energies= -1848.154477
Sum of electronic and thermal Energies= -1848.119137
Sum of electronic and thermal Enthalpies= -1848.118193
Sum of electronic and thermal Free Energies= -1848.220811

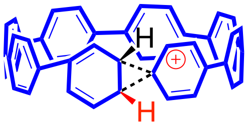

22c

Charge = 1 Multiplicity = 1

H	4.16474	-0.56509	1.02238
C	4.14763	-1.53812	0.54793
C	4.02151	-4.04064	-0.69552
C	5.14	-1.90062	-0.38879
C	3.10507	-2.39339	0.82073

C	2.9661	-3.6444	0.16195
C	5.09861	-3.20226	-0.94683
H	2.32207	-2.05496	1.48866
H	5.87786	-3.52684	-1.63114
H	3.98759	-5.01045	-1.18139
C	6.87236	-1.17071	0.00272
C	5.55392	1.38742	-0.09825
C	6.99812	-0.24529	1.08462
C	6.0675	-0.79681	-1.15028
C	5.49305	0.54857	-1.18106
C	6.3776	0.97567	1.02705
H	7.62362	-0.50748	1.93124
H	4.90205	0.81446	-2.05085
H	6.51992	1.67803	1.84315
H	7.50286	-2.05086	-0.03666
H	6.33532	-1.21898	-2.11566
C	1.69175	-4.35773	0.29396
C	-1.10745	-4.91311	0.26474
C	1.0879	-4.99987	-0.81007
C	0.90341	-4.20922	1.45677
C	-0.45047	-4.49255	1.44588
C	-0.2762	-5.27197	-0.82163
H	1.66327	-5.18602	-1.71219
H	1.34792	-3.8188	2.3671
H	-1.025	-4.27542	2.3383
H	-0.71144	-5.68845	-1.72371
C	4.68538	2.58459	-0.02049
C	2.40282	4.28671	0.01474
C	4.0064	2.86116	1.1803
C	4.31008	3.31646	-1.16363
C	3.20595	4.16115	-1.14002
C	2.88553	3.68728	1.19388
H	4.27826	2.34297	2.09591
H	4.87009	3.20303	-2.08787
H	2.93287	4.69202	-2.04633
H	2.31739	3.77545	2.11364
C	-2.56264	-4.68897	0.12702
C	-4.91254	-3.09439	-0.15696
C	-3.37059	-4.3717	1.24112
C	-3.13186	-4.44226	-1.1413
C	-4.28704	-3.68214	-1.27859
C	-4.51003	-3.5907	1.10298
H	-3.05938	-4.65117	2.24228
H	-2.60771	-4.73791	-2.04388

H	-4.62863	-3.44805	-2.28079
H	-5.01571	-3.26392	2.0047
C	-5.65028	-1.80923	-0.25874
C	-5.86201	1.02992	-0.14412
C	-6.19545	-1.16151	0.87268
C	-5.49975	-1.01679	-1.41521
C	-5.60437	0.36777	-1.36004
C	-6.2971	0.22559	0.92997
H	-6.45706	-1.73272	1.7577
H	-5.14548	-1.45776	-2.33962
H	-5.31974	0.9404	-2.23656
H	-6.62932	0.68926	1.85431
C	1.0088	4.79179	-0.04153
C	-1.84062	4.84056	0.00914
C	0.30775	5.22781	1.10121
C	0.25493	4.58228	-1.21148
C	-1.13524	4.60839	-1.18697
C	-1.08469	5.25641	1.12454
H	0.85191	5.49973	2.00119
H	0.74695	4.2657	-2.12539
H	-1.67509	4.308	-2.07885
H	-1.5897	5.54325	2.04223
C	-5.31064	2.39668	0.03929
C	-3.2347	4.33913	0.116
C	-4.62288	2.6791	1.23516
C	-5.11184	3.28918	-1.03496
C	-4.09347	4.23823	-0.99811
C	-3.60674	3.62933	1.2726
H	-4.76699	2.04297	2.10226
H	-5.70128	3.18526	-1.94137
H	-3.91486	4.85267	-1.87601
H	-2.99881	3.70091	2.16882

Imaginary Frequency = -233.3030

Zero-point correction= 0.654625 (Hartree/Particle)
Thermal correction to Energy= 0.689484
Thermal correction to Enthalpy= 0.690428
Thermal correction to Gibbs Free Energy= 0.589212
Sum of electronic and zero-point Energies= -1848.121289
Sum of electronic and thermal Energies= -1848.086430
Sum of electronic and thermal Enthalpies= -1848.085486
Sum of electronic and thermal Free Energies= -1848.186702

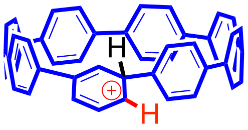

22d

Charge = 1 Multiplicity = 1

H	5.0486	-2.19032	2.58131
C	4.74736	-2.44016	1.56681
C	3.93983	-3.10297	-1.00856
C	5.43263	-1.89465	0.47189
C	3.64388	-3.26402	1.37253
C	3.17257	-3.57231	0.07876
C	5.03033	-2.25712	-0.82125
H	3.10439	-3.61768	2.24417
H	5.55945	-1.87441	-1.68933
H	3.66801	-3.37738	-2.02201
C	7.52606	-0.61065	-0.30025
C	5.6331	1.55063	0.01919
C	7.64146	0.52717	-1.04685
C	6.46103	-0.74738	0.72247
C	5.5791	0.44089	0.85753
C	6.70434	1.58667	-0.90132
H	8.44296	0.6331	-1.77092
H	4.79444	0.36951	1.60238
H	6.82426	2.46453	-1.53014
C	1.84379	-4.20036	-0.10431
C	-0.95655	-4.74341	-0.2011
C	1.08249	-3.91655	-1.25547
C	1.20135	-4.925	0.92334
C	-0.1637	-5.18729	0.87836
C	-0.27871	-4.18741	-1.30555
H	1.52661	-3.37555	-2.08404
H	1.76572	-5.25989	1.78816
H	-0.62868	-5.70561	1.71157
H	-0.84596	-3.84607	-2.16501
C	4.56915	2.58557	0.053
C	2.16398	4.11254	0.00028
C	3.8547	2.85394	1.23392
C	4.13088	3.20842	-1.12927
C	2.96198	3.96582	-1.15112
C	2.68071	3.59841	1.20647
H	4.19268	2.44861	2.18343

H	4.66214	3.04916	-2.06363
H	2.63961	4.40132	-2.09118
H	2.11615	3.71398	2.1255
C	-2.42328	-4.57427	-0.11343
C	-4.89354	-3.16443	-0.06007
C	-2.98777	-4.14669	1.10481
C	-3.25022	-4.51449	-1.25661
C	-4.46312	-3.83783	-1.2273
C	-4.19323	-3.4555	1.13017
H	-2.40904	-4.21322	2.02055
H	-2.9079	-4.94389	-2.19378
H	-5.03918	-3.75696	-2.1438
H	-4.50609	-3.00404	2.06517
C	-5.7386	-1.94965	-0.11384
C	-6.09758	0.86837	-0.00389
C	-6.25277	-1.32687	1.04734
C	-5.719	-1.16729	-1.28772
C	-5.89491	0.20932	-1.23402
C	-6.42939	0.05076	1.10084
H	-6.42662	-1.91111	1.94599
H	-5.39288	-1.60326	-2.2255
H	-5.69678	0.78646	-2.13068
H	-6.73353	0.50455	2.0394
C	0.75579	4.57802	-0.06498
C	-2.0913	4.65309	0.00767
C	0.06883	5.08443	1.05661
C	-0.00941	4.29317	-1.21057
C	-1.39902	4.33343	-1.17597
C	-1.32339	5.12363	1.09172
H	0.62368	5.40947	1.93197
H	0.47233	3.91398	-2.10588
H	-1.95077	3.98093	-2.0411
H	-1.81849	5.46784	1.99503
C	-5.57422	2.24713	0.14758
C	-3.49199	4.18116	0.14764
C	-4.88021	2.56907	1.33169
C	-5.38851	3.11423	-0.95204
C	-4.36557	4.05715	-0.95344
C	-3.86078	3.51541	1.33152
H	-5.01721	1.96169	2.21986
H	-5.98839	2.98973	-1.84868
H	-4.19146	4.64527	-1.85006
H	-3.24493	3.61314	2.21983
H	8.22025	-1.43649	-0.42779

H	6.94537	-0.97002	1.68977

Zero-point correction= 0.654692 (Hartree/Particle)
Thermal correction to Energy= 0.690223
Thermal correction to Enthalpy= 0.691167
Thermal correction to Gibbs Free Energy= 0.588027
Sum of electronic and zero-point Energies= -1848.135540
Sum of electronic and thermal Energies= -1848.100009
Sum of electronic and thermal Enthalpies= -1848.099064
Sum of electronic and thermal Free Energies= -1848.202204

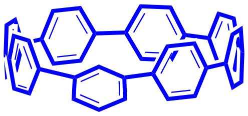

22e

Charge = 0 Multiplicity = 1

H	-2.12934	4.19183	2.01067
C	-1.25963	4.03202	2.61661
C	0.9886	3.57575	4.14522
C	-0.14341	4.83887	2.43254
C	-1.22505	2.97013	3.49649
C	-0.0753	2.68385	4.2263
C	0.95643	4.63841	3.25591
H	-2.0598	2.30045	3.54466
H	1.82144	5.26213	3.1441
H	1.87326	3.40064	4.72553
C	0.20864	7.06247	1.20828
C	-0.06231	5.70211	-1.21483
C	0.3356	7.7319	0.
C	-0.06231	5.70211	1.21483
C	-0.19512	5.045	0.
C	0.20864	7.06247	-1.20828
H	0.53534	8.78535	0.
H	-0.35888	3.98701	0.
H	0.31386	7.59431	-2.13327
C	0.06527	1.30923	4.79527
C	0.074	-1.49331	4.98565
C	1.18765	0.57277	4.43547
C	-0.98637	0.63345	5.40781
C	-0.98199	-0.74826	5.50244
C	1.19169	-0.80333	4.52905
H	2.01137	1.06515	3.95953

H	-1.84136	1.18247	5.75113
H	-1.83519	-1.25105	5.9144
H	2.01657	-1.35028	4.11983
C	-0.14341	4.83887	-2.43254
C	-0.0753	2.68385	-4.2263
C	-1.25963	4.03202	-2.61661
C	0.95643	4.63841	-3.25591
C	0.9886	3.57575	-4.14522
C	-1.22505	2.97013	-3.49649
H	-2.12934	4.19183	-2.01067
H	1.82144	5.26213	-3.1441
H	1.87326	3.40064	-4.72553
H	-2.0598	2.30045	-3.54466
C	-0.06655	-2.91809	4.55257
C	-0.04744	-5.06857	2.7481
C	-1.20344	-3.24812	3.82463
C	1.00518	-3.80659	4.51637
C	1.01362	-4.86894	3.62817
C	-1.1936	-4.30421	2.93729
H	-2.04715	-2.58832	3.83649
H	1.87146	-3.61842	5.12024
H	1.8871	-5.4874	3.55695
H	-2.02872	-4.43448	2.27982
C	0.12082	-5.70618	1.40362
C	0.12082	-5.70618	-1.40362
C	-0.93846	-6.26561	0.69245
C	1.26245	-5.36045	0.68954
C	1.26245	-5.36045	-0.68954
C	-0.93846	-6.26561	-0.69245
H	-1.80676	-6.61343	1.2175
H	2.09883	-4.93473	1.20518
H	2.09883	-4.93473	-1.20518
H	-1.80676	-6.61343	-1.2175
C	0.06527	1.30923	-4.79527
C	0.074	-1.49331	-4.98565
C	-0.98637	0.63345	-5.40781
C	1.18765	0.57277	-4.43547
C	1.19169	-0.80333	-4.52905
C	-0.98199	-0.74826	-5.50244
H	-1.84136	1.18247	-5.75113
H	2.01137	1.06515	-3.95953
H	2.01657	-1.35028	-4.11983
H	-1.83519	-1.25105	-5.9144
C	-0.04744	-5.06857	-2.7481

C	-0.06655	-2.91809	-4.55257
C	-1.1936	-4.30421	-2.93729
C	1.01362	-4.86894	-3.62817
C	1.00518	-3.80659	-4.51637
C	-1.20344	-3.24812	-3.82463
H	-2.02872	-4.43448	-2.27982
H	1.8871	-5.4874	-3.55695
H	1.87146	-3.61842	-5.12024
H	-2.04715	-2.58832	-3.83649
H	0.31386	7.59431	2.13327

Zero-point correction= 0.644077 (Hartree/Particle)
Thermal correction to Energy= 0.679075
Thermal correction to Enthalpy= 0.680019
Thermal correction to Gibbs Free Energy= 0.578247
Sum of electronic and zero-point Energies= -1847.829588
Sum of electronic and thermal Energies= -1847.794591
Sum of electronic and thermal Enthalpies= -1847.793646
Sum of electronic and thermal Free Energies= -1847.895419

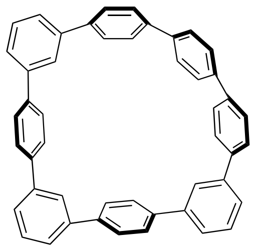

22f

Charge = 0 Multiplicity = 1

C	-6.96062	-1.52404	-0.45097
C	-7.59747	-0.28597	-0.59705
C	-6.9192	0.91217	-0.35105
C	-5.57735	0.88993	0.06414
C	-4.96512	-0.36128	0.23011
C	-5.62504	-1.57298	-0.02655
C	-4.7869	-2.80136	0.08123
C	-4.72786	2.0999	0.24012
C	-3.74938	2.12731	1.25075
C	-2.76102	3.10567	1.27092
C	-2.7153	4.11218	0.29073
C	-3.76201	4.16052	-0.64432
C	-4.74812	3.16908	-0.67187

C	-1.50449	4.97431	0.18032
C	-1.53081	6.37601	0.11986
C	-0.33427	7.09243	0.01003
C	0.89714	6.43384	-0.06592
C	0.94739	5.0311	-0.03609
C	-0.26066	4.33026	0.10406
C	2.20047	4.23827	-0.17061
C	2.20252	3.114	-1.0151
C	3.2814	2.23968	-1.04602
C	4.412	2.44539	-0.23477
C	4.45349	3.61728	0.53843
C	3.366	4.49839	0.5699
C	5.43197	1.36277	-0.15506
C	6.81992	1.55225	-0.25016
C	7.67984	0.44886	-0.22241
C	7.18569	-0.85622	-0.098
C	5.80613	-1.0658	0.02805
C	4.95933	0.05206	0.00267
C	-4.02479	-3.01369	1.24219
C	-2.93547	-3.87901	1.23784
C	-2.55623	-4.56511	0.0686
C	-3.42915	-4.49105	-1.03281
C	-4.52512	-3.62445	-1.02722
C	-1.15287	-5.04691	-0.03735
C	-0.42044	-5.49558	1.07906
C	0.97218	-5.43969	1.09531
C	1.69147	-4.93509	-0.00521
C	0.96812	-4.70306	-1.18917
C	-0.42342	-4.75701	-1.20476
C	3.04741	-4.33763	0.13004
C	3.94612	-4.21744	-0.94595
C	4.95945	-3.25468	-0.9335
C	5.10731	-2.38101	0.15633
C	4.33005	-2.62663	1.29962
C	3.32464	-3.58931	1.28948
H	-7.50194	-2.44318	-0.6572
H	-8.63794	-0.25495	-0.90839
H	-7.43497	1.86024	-0.47559
H	-3.91298	-0.39704	0.49036
H	-3.74503	1.35308	2.01204
H	-1.99523	3.07785	2.04085
H	-3.75879	4.92984	-1.41177
H	-5.48997	3.18239	-1.46554
H	-2.47807	6.90445	0.18166

H	-0.3626	8.17797	-0.02489
H	1.81264	7.00715	-0.18199
H	-0.23474	3.24603	0.14368
H	1.34102	2.9202	-1.64701
H	3.24644	1.37372	-1.70006
H	5.31189	3.80923	1.17668
H	3.40003	5.35988	1.23147
H	7.22532	2.55212	-0.3786
H	8.75127	0.60664	-0.30968
H	7.87034	-1.69957	-0.08257
H	3.89084	-0.10903	0.10168
H	-4.23646	-2.42801	2.13233
H	-2.30172	-3.92731	2.1177
H	-3.20601	-5.05309	-1.93552
H	-5.11995	-3.51279	-1.9299
H	-0.94592	-5.81947	1.9732
H	1.50172	-5.71955	2.00193
H	1.48439	-4.33074	-2.06869
H	-0.94667	-4.42462	-2.09599
H	3.80328	-4.82233	-1.83723
H	5.57342	-3.11517	-1.81937
H	4.44971	-1.98876	2.17087
H	2.66507	-3.6672	2.14807

Zero-point correction= 0.644441 (Hartree/Particle)
Thermal correction to Energy= 0.679747
Thermal correction to Enthalpy= 0.680692
Thermal correction to Gibbs Free Energy= 0.577267
Sum of electronic and zero-point Energies= -1847.870520
Sum of electronic and thermal Energies= -1847.835214
Sum of electronic and thermal Enthalpies= -1847.834269
Sum of electronic and thermal Free Energies= -1847.937694

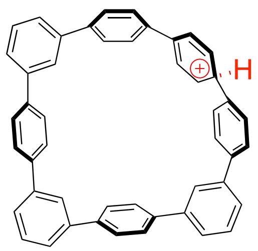

22g

Charge = 1 Multiplicity = 1

C	-3.53407	4.07493	0.18417

C	-6.11147	3.03357	-0.05792
C	-3.76776	2.71127	0.26962
C	-4.61213	4.92469	-0.01897
C	-5.8906	4.40082	-0.13135
C	-5.04115	2.1769	0.14703
H	-2.93564	2.0475	0.38768
H	-4.45631	5.9834	-0.08209
H	-6.72101	5.0624	-0.27814
H	-7.10442	2.64117	-0.15321
C	-2.10897	4.51366	0.23578
C	0.66732	4.82806	0.12206
C	-1.30615	4.11792	1.30006
C	-1.51527	5.15563	-0.84164
C	-0.13954	5.31144	-0.89812
C	0.06397	4.27167	1.24385
H	-1.75858	3.66147	2.15792
H	-2.11997	5.46816	-1.66973
H	0.31049	5.74514	-1.76926
H	0.6741	3.93602	2.05914
C	-5.13038	0.68646	0.17153
C	-4.68859	-2.06901	0.05775
C	-4.69014	-0.00581	1.29373
C	-5.4501	-0.03124	-0.97168
C	-5.2298	-1.3985	-1.02931
C	-4.47178	-1.36693	1.23794
H	-4.48149	0.53449	2.19553
H	-5.79637	0.48909	-1.84236
H	-5.40598	-1.92637	-1.94581
H	-4.09884	-1.88422	2.10003
C	-4.14567	-3.45135	-0.08405
C	-2.89988	-5.92389	-0.47034
C	-2.78464	-3.60744	0.13154
C	-4.88494	-4.55509	-0.4838
C	-4.26259	-5.78194	-0.66575
C	-2.15172	-4.82702	-0.0608
H	-2.20652	-2.74646	0.39822
H	-5.93971	-4.45918	-0.64695
H	-4.84466	-6.63108	-0.9622
H	-2.43214	-6.87758	-0.61576
C	-0.68009	-4.8512	0.10949
C	2.09691	-4.4436	0.26294
C	0.16651	-5.35473	-0.88306
C	-0.11084	-4.24284	1.23046
C	1.24167	-4.04855	1.31089

C	1.52011	-5.14138	-0.81831
H	-0.25509	-5.8506	-1.73288
H	-0.74658	-3.90051	2.0199
H	1.62854	-3.52805	2.15991
H	2.13184	-5.5061	-1.61642
C	3.45195	-3.97367	0.24475
C	5.78816	-2.29808	0.05911
C	4.20171	-3.89842	-0.98627
C	4.07156	-3.45921	1.43463
C	5.16707	-2.69613	1.35615
C	5.28451	-3.11988	-1.07712
H	3.83967	-4.40853	-1.85131
H	3.63765	-3.67711	2.38514
H	5.60069	-2.28548	2.24647
C	5.39904	-0.80078	-0.12799
C	4.58663	1.8749	-0.22969
C	4.23969	-0.4446	-0.79711
C	6.17019	0.19004	0.45838
C	5.76441	1.51401	0.4138
C	3.84943	0.87609	-0.85797
H	3.63441	-1.18658	-1.27405
H	7.07883	-0.06231	0.97097
H	6.3522	2.26228	0.90615
H	2.96363	1.13746	-1.39958
C	4.06787	3.27096	-0.23041
C	2.96489	5.82848	-0.30307
C	2.71378	3.47231	-0.00534
C	4.87676	4.37261	-0.47819
C	4.32266	5.64113	-0.50797
C	2.14665	4.73767	-0.048
H	2.07956	2.63297	0.19567
H	5.92247	4.24102	-0.67425
H	4.95024	6.48738	-0.70386
H	2.5462	6.81453	-0.33324
H	5.79184	-3.01238	-2.01604
H	6.87002	-2.36559	0.11574

Zero-point correction= 0.656603 (Hartree/Particle)
Thermal correction to Energy= 0.692205
Thermal correction to Enthalpy= 0.693149
Thermal correction to Gibbs Free Energy= 0.589468
Sum of electronic and zero-point Energies= -1848.212506
Sum of electronic and thermal Energies= -1848.176904
Sum of electronic and thermal Enthalpies= -1848.175960
Sum of electronic and thermal Free Energies= -1848.279640

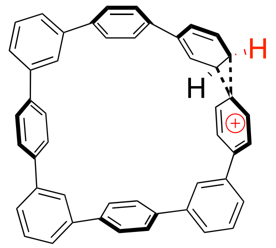

22h

Charge = 1 Multiplicity = 1

C	-4.27847	3.41693	-0.02416
C	-6.66362	1.93378	0.23267
C	-4.26609	2.0222	0.10219
C	-5.51625	4.07325	0.01403
C	-6.69299	3.32855	0.13522
C	-5.43436	1.26189	0.23356
H	-3.31194	1.50603	0.09201
H	-5.56452	5.15392	-0.08342
H	-7.6492	3.84308	0.14887
H	-7.58999	1.3757	0.33166
C	-2.95302	4.07072	-0.20169
C	-0.2252	4.83846	-0.27693
C	-2.47046	5.09149	0.63127
C	-2.07388	3.53328	-1.15797
C	-0.73923	3.90725	-1.19459
C	-1.12513	5.46918	0.59595
H	-3.12986	5.53613	1.37093
H	-2.44354	2.79175	-1.85905
H	-0.07852	3.4603	-1.93165
H	-0.75881	6.1996	1.31201
C	-5.23525	-0.21184	0.33962
C	-4.23172	-2.86149	0.22262
C	-4.37062	-0.70284	1.33201
C	-5.69871	-1.11196	-0.63064
C	-5.20028	-2.41553	-0.69087
C	-3.88536	-2.0022	1.27942
H	-4.0477	-0.03871	2.12821
H	-6.37939	-0.76421	-1.4024
H	-5.49734	-3.0585	-1.51441
H	-3.19865	-2.34693	2.04676
C	-3.44772	-4.1039	-0.0033
C	-1.76298	-6.28574	-0.62975
C	-2.06125	-4.04175	0.19872

C	-3.98281	-5.30017	-0.50536
C	-3.14523	-6.37703	-0.80502
C	-1.20618	-5.106	-0.11501
H	-1.6255	-3.10489	0.52796
H	-5.05406	-5.39123	-0.65792
H	-3.57586	-7.29929	-1.18301
H	-1.12555	-7.13018	-0.87506
C	0.25251	-4.86565	0.03254
C	2.9215	-3.91844	0.16619
C	1.15509	-5.09509	-1.0213
C	0.74726	-4.2558	1.19766
C	2.05722	-3.79858	1.269
C	2.46131	-4.6175	-0.96351
H	0.80679	-5.59181	-1.92158
H	0.08281	-4.11023	2.04308
H	2.38311	-3.28152	2.16729
H	3.12191	-4.77127	-1.81207
C	4.18649	-3.15043	0.16865
C	6.43344	-1.3773	0.3787
C	4.62562	-2.47878	-0.944
C	4.94342	-2.9921	1.39667
C	6.00869	-2.14027	1.50753
C	5.71226	-1.50552	-0.87498
H	4.10172	-2.5779	-1.88839
H	4.6607	-3.59862	2.25151
H	6.56774	-2.06688	2.43373
C	5.27162	-0.03005	-0.29452
C	4.12965	2.54931	-0.08369
C	5.9531	1.09978	-0.80039
C	4.06345	0.15816	0.40649
C	3.51443	1.4166	0.50522
C	5.38516	2.36208	-0.70014
H	6.89717	0.97742	-1.32404
H	3.5457	-0.68299	0.84999
H	2.58502	1.54034	1.04829
H	5.89076	3.20594	-1.15722
C	3.42002	3.84098	-0.07672
C	1.92148	6.21913	-0.03636
C	2.01959	3.82503	-0.17345
C	4.07106	5.08413	0.02109
C	3.31668	6.25586	0.05282
C	1.24901	4.99395	-0.16802
H	1.50369	2.87695	-0.27139
H	5.15139	5.13429	0.11678

H	3.82052	7.21258	0.14749
H	1.35584	7.14588	-0.02757
H	7.38283	-0.85708	0.40763
H	6.25335	-1.32371	-1.79977

Imaginary frequency = -209.2856

Zero-point correction= 0.655230 (Hartree/Particle)
Thermal correction to Energy= 0.690443
Thermal correction to Enthalpy= 0.691387
Thermal correction to Gibbs Free Energy= 0.588700
Sum of electronic and zero-point Energies= -1848.184464
Sum of electronic and thermal Energies= -1848.149251
Sum of electronic and thermal Enthalpies= -1848.148307
Sum of electronic and thermal Free Energies= -1848.250994

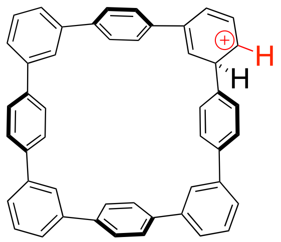

22i

Charge = 1 Multiplicity = 1

C	-4.52537	3.04216	0.06525
C	-6.77793	1.41285	0.30422
C	-4.41441	1.66133	0.08481
C	-5.78499	3.61174	0.18081
C	-6.89993	2.79545	0.29527
C	-5.52191	0.83743	0.20505
H	-3.44326	1.21564	0.01136
H	-5.89668	4.67778	0.16447
H	-7.87226	3.23934	0.37586
H	-7.64821	0.79444	0.39835
C	-3.24851	3.80342	-0.06833
C	-0.62778	4.77069	-0.15247
C	-2.76797	4.6233	0.94196
C	-2.43299	3.54796	-1.16562
C	-1.14001	4.02341	-1.20676
C	-1.46711	5.10374	0.90034

H	-3.38534	4.83223	1.79308
H	-2.80942	2.94815	-1.96999
H	-0.51281	3.79683	-2.04618
H	-1.08818	5.68336	1.71903
C	-5.22635	-0.62899	0.21576
C	-4.0439	-3.15344	0.06196
C	-4.58232	-1.17484	1.31916
C	-5.36701	-1.40189	-0.9265
C	-4.78015	-2.65585	-1.00326
C	-3.99891	-2.42292	1.24384
H	-4.49704	-0.59551	2.21688
H	-5.86844	-0.99494	-1.78206
H	-4.82886	-3.21039	-1.91963
H	-3.46825	-2.81733	2.08787
C	-3.14021	-4.32854	-0.10741
C	-1.26774	-6.3552	-0.52695
C	-1.78243	-4.09488	0.05906
C	-3.55678	-5.59648	-0.48459
C	-2.62195	-6.6008	-0.68566
C	-0.84102	-5.09028	-0.14817
H	-1.45633	-3.10585	0.30994
H	-4.60121	-5.79944	-0.61338
H	-2.95158	-7.58128	-0.96582
H	-0.5556	-7.14106	-0.68456
C	0.59306	-4.70807	-0.00875
C	3.15642	-3.61556	0.15576
C	1.48116	-4.82571	-1.07067
C	1.02946	-4.09633	1.16184
C	2.29379	-3.55071	1.24264
C	2.75237	-4.28296	-0.99174
H	1.16114	-5.3015	-1.97556
H	0.36145	-4.02149	1.99558
H	2.58874	-3.03533	2.13686
H	3.41633	-4.36387	-1.83057
C	4.43757	-2.85697	0.21198
C	6.77245	-1.25907	0.58993
C	4.71106	-1.8632	-0.67586
C	5.36554	-3.06538	1.24918
C	6.52302	-2.28088	1.4361
C	5.86614	-0.94453	-0.53684
H	4.03756	-1.66748	-1.48722
H	5.17375	-3.86642	1.938
H	7.18013	-2.50273	2.25042
C	5.25437	0.48122	-0.36568

C	3.92258	2.91818	-0.17653
C	5.42526	1.44127	-1.34618
C	4.45468	0.7525	0.7345
C	3.79685	1.96085	0.82644
C	4.76597	2.65684	-1.24632
H	6.04332	1.24625	-2.2007
H	4.32684	0.02033	1.50739
H	3.16665	2.16355	1.66831
H	4.87473	3.38484	-2.02455
C	3.07465	4.1442	-0.13385
C	1.37044	6.33882	-0.05827
C	1.69943	3.97488	-0.14768
C	3.60088	5.427	-0.0903
C	2.74521	6.51544	-0.04642
C	0.83502	5.06058	-0.11852
H	1.28849	2.98593	-0.18653
H	4.66263	5.57465	-0.07302
H	3.15143	7.50623	-0.0056
H	0.71824	7.18897	-0.03922
H	6.44089	-0.93267	-1.46345
H	7.63294	-0.63182	0.71544

Zero-point correction= 0.654669 (Hartree/Particle)
Thermal correction to Energy= 0.690732
Thermal correction to Enthalpy= 0.691676
Thermal correction to Gibbs Free Energy= 0.585765
Sum of electronic and zero-point Energies= -1848.193047
Sum of electronic and thermal Energies= -1848.156984
Sum of electronic and thermal Enthalpies= -1848.156040
Sum of electronic and thermal Free Energies= -1848.261951

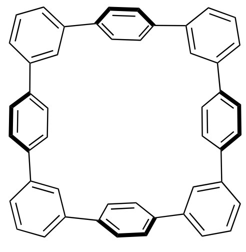

22j

Charge = 0 Multiplicity = 1

C	3.69251	-5.65656	-0.37126
C	2.82488	-6.73699	-0.56081

C	1.44601	-6.59797	-0.37248
C	0.91405	-5.3636	0.03274
C	1.80186	-4.29684	0.23882
C	3.18512	-4.41173	0.03362
C	3.9997	-3.17291	0.16393
C	-0.5404	-5.07628	0.16324
C	-1.46027	-5.41459	-0.84334
C	-2.75415	-4.88545	-0.84292
C	-3.17291	-3.9997	0.16396
C	-2.29256	-3.76769	1.23572
C	-1.00544	-4.29417	1.2354
C	-4.41173	-3.18512	0.03366
C	-5.65657	-3.69251	-0.37122
C	-6.73699	-2.82488	-0.56076
C	-6.59797	-1.44601	-0.37244
C	-5.3636	-0.91405	0.03277
C	-4.29684	-1.80185	0.23885
C	-5.07628	0.5404	0.16326
C	-5.4146	1.46026	-0.84332
C	-4.88546	2.75415	-0.84292
C	-3.9997	3.17291	0.16397
C	-3.76768	2.29257	1.23572
C	-4.29417	1.00545	1.23541
C	-3.18512	4.41173	0.03365
C	-3.69251	5.65656	-0.37123
C	-2.82488	6.73699	-0.56078
C	-1.44602	6.59797	-0.37247
C	-0.91405	5.3636	0.03274
C	-1.80185	4.29684	0.23884
C	3.76769	-2.29257	1.23569
C	4.29418	-1.00545	1.23537
C	5.07628	-0.5404	0.16321
C	5.41459	-1.46027	-0.84337
C	4.88545	-2.75415	-0.84296
C	5.3636	0.91405	0.03272
C	4.29684	1.80185	0.23881
C	4.41173	3.18512	0.03361
C	5.65656	3.69251	-0.37127
C	6.73698	2.82488	-0.56083
C	6.59797	1.44601	-0.37251
C	3.17292	3.9997	0.16393
C	2.29257	3.76769	1.23569
C	1.00545	4.29417	1.23538
C	0.5404	5.07628	0.16323

C	1.46026	5.41459	-0.84336
C	2.75414	4.88545	-0.84295
H	4.75995	-5.78329	-0.52883
H	3.22871	-7.70029	-0.8598
H	0.78789	-7.44772	-0.53097
H	1.39363	-3.32315	0.48782
H	-1.13487	-6.02066	-1.68442
H	-3.41143	-5.08948	-1.68373
H	-2.6126	-3.13979	2.06217
H	-0.33671	-4.07073	2.06162
H	-5.78329	-4.75995	-0.52877
H	-7.7003	-3.22871	-0.85974
H	-7.44772	-0.78789	-0.53094
H	-3.32315	-1.39362	0.48784
H	-6.02067	1.13486	-1.68439
H	-5.08948	3.41143	-1.68372
H	-3.13978	2.61261	2.06217
H	-4.07072	0.33672	2.06163
H	-4.75996	5.78329	-0.52878
H	-3.22871	7.70029	-0.85977
H	-0.78789	7.44772	-0.53097
H	-1.39362	3.32315	0.48783
H	3.1398	-2.61261	2.06214
H	4.07074	-0.33672	2.0616
H	6.02066	-1.13486	-1.68445
H	5.08947	-3.41143	-1.68377
H	3.32316	1.39362	0.48781
H	5.78329	4.75995	-0.52883
H	7.70029	3.22871	-0.85982
H	7.44772	0.78789	-0.53101
H	2.61262	3.13979	2.06214
H	0.33673	4.07073	2.06161
H	1.13486	6.02066	-1.68443
H	3.41142	5.08947	-1.68376

Zero-point correction= 0.644884 (Hartree/Particle)
Thermal correction to Energy= 0.680135
Thermal correction to Enthalpy= 0.681080
Thermal correction to Gibbs Free Energy= 0.578240
Sum of electronic and zero-point Energies= -1847.894196
Sum of electronic and thermal Energies= -1847.858945
Sum of electronic and thermal Enthalpies= -1847.858001
Sum of electronic and thermal Free Energies= -1847.960840

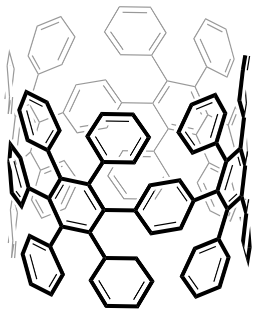

27

Charge = 0 Multiplicity = 1

C	-0.39217	-5.05819	-0.0545
C	-3.10117	-4.24387	0.12235
C	-1.42411	-5.99732	-0.12149
C	-0.73165	-3.70274	0.11487
C	-2.05346	-3.30558	0.19973
C	-2.77067	-5.59236	-0.03769
H	-1.18928	-7.04777	-0.24133
H	0.0639	-2.96862	0.17972
H	-2.30112	-2.25755	0.31939
H	-3.55557	-6.33461	-0.11306
C	1.10196	-5.25455	-0.11655
C	3.74012	-4.28188	-0.08036
C	1.76857	-4.99004	-1.32889
C	1.85297	-5.33915	1.0791
C	3.17751	-4.84307	1.09776
C	3.10572	-4.53437	-1.32062
C	-4.44346	-3.54938	0.14109
C	-5.79047	-1.08791	0.04107
C	-4.83426	-2.91443	1.34034
C	-5.04105	-3.14038	-1.07376
C	-5.71056	-1.89531	-1.12454
C	-5.53781	-1.69294	1.29709
C	4.63357	-3.07138	0.06148
C	5.66406	-0.40903	0.10348
C	5.78822	-2.73045	-0.65611
C	4.05933	-2.07116	0.874
C	4.55502	-0.78306	0.89101
C	6.30187	-1.42005	-0.62854
H	6.26858	-3.46622	-1.28714
H	3.16821	-2.30606	1.44025
H	4.04333	-0.02362	1.46671
H	7.16803	-1.1851	-1.2327
C	-5.66416	0.40916	-0.10366

C	-4.63337	3.07137	-0.06125
C	-4.55499	0.78315	-0.89102
C	-6.30196	1.42018	0.62839
C	-5.78812	2.73051	0.65619
C	-4.05915	2.07118	-0.87382
H	-4.04332	0.02371	-1.46673
H	-7.16824	1.18528	1.23238
H	-6.26847	3.46629	1.28724
H	-3.16795	2.30607	-1.43996
C	5.79022	1.08804	-0.04138
C	4.44335	3.54959	-0.14151
C	5.53735	1.6929	-1.29744
C	5.71046	1.89554	1.12415
C	5.04104	3.14067	1.07331
C	4.83389	2.91443	-1.34073
C	3.10112	4.2442	-0.12267
C	0.3922	5.05872	0.05441
C	2.77074	5.59271	0.03744
C	2.05334	3.30599	-0.20002
C	0.73157	3.70325	-0.11505
C	1.4242	5.99777	0.12136
H	3.55569	6.3349	0.11279
H	2.3009	2.25795	-0.31976
H	1.18947	7.04824	0.24125
C	-3.73991	4.28182	0.08075
C	-1.10193	5.25507	0.11666
C	-3.10528	4.53403	1.3209
C	-3.17762	4.8434	-1.09735
C	-1.85315	5.33961	-1.07886
C	-1.7683	4.99024	1.32906
C	-1.05157	5.17333	2.63748
C	0.15203	5.52399	5.15207
C	-0.20723	4.18644	3.16463
C	-1.26884	6.34575	3.37347
C	-0.66899	6.52163	4.62174
C	0.38471	4.35721	4.41815
H	-0.02316	3.28417	2.59465
H	-1.9228	7.10944	2.96985
H	-0.85063	7.43093	5.18316
H	1.02459	3.58094	4.82222
H	0.60305	5.65105	6.12985
C	-1.24882	5.97763	-2.29553
C	-0.15111	7.28492	-4.53016
C	-0.09952	5.47412	-2.92287

C	-1.8402	7.14635	-2.80335
C	-1.29628	7.7941	-3.91175
C	0.44388	6.12444	-4.03312
H	0.37935	4.58327	-2.54104
H	-2.73116	7.53661	-2.32676
H	-1.76462	8.69549	-4.29058
H	1.34078	5.72452	-4.49009
H	0.274	7.79101	-5.38957
C	-3.9971	4.82437	-2.34927
C	-5.61307	4.81166	-4.65349
C	-3.49515	4.35218	-3.57277
C	-5.32087	5.29247	-2.30177
C	-6.12159	5.2883	-3.44373
C	-4.29835	4.34262	-4.71229
H	-2.47367	4.00007	-3.63058
H	-5.71428	5.66161	-1.36236
H	-7.14009	5.65529	-3.3885
H	-3.89747	3.96835	-5.64725
H	-6.2345	4.80452	-5.54149
C	-3.82669	4.32856	2.61611
C	-5.08757	4.23241	5.1301
C	-3.38526	3.43336	3.60116
C	-4.92393	5.1583	2.90163
C	-5.55587	5.10458	4.14536
C	-4.00169	3.39666	4.85325
H	-2.53751	2.79334	3.39861
H	-5.25821	5.8634	2.14891
H	-6.39713	5.75639	4.35159
H	-3.62614	2.72382	5.61637
H	-5.55905	4.208	6.10595
C	-6.04721	-1.07743	2.56698
C	-7.17299	0.00287	4.90244
C	-7.17918	-1.66523	3.15384
C	-5.48366	0.05555	3.16528
C	-6.04943	0.59441	4.3227
C	-7.73569	-1.13339	4.31713
H	-7.61571	-2.54277	2.69174
H	-4.623	0.53428	2.71597
H	-5.61948	1.49112	4.74734
H	-8.60775	-1.60097	4.76004
H	-7.60922	0.42658	5.79994
C	-4.43171	-3.52785	2.64515
C	-3.7575	-4.72015	5.10201
C	-4.85637	-4.83075	2.94929

C	-3.64901	-2.83714	3.58205
C	-3.31501	-3.42839	4.80086
C	-4.52362	-5.42259	4.16833
H	-5.4603	-5.36853	2.22793
H	-3.31195	-1.83386	3.35733
H	-2.71591	-2.87964	5.51859
H	-4.86815	-6.42567	4.39229
H	-3.51334	-5.17082	6.05769
C	-4.9963	-4.04741	-2.26778
C	-5.03794	-5.85173	-4.42414
C	-6.2121	-4.5636	-2.74722
C	-3.80212	-4.44606	-2.88462
C	-3.82553	-5.34393	-3.95516
C	-6.23322	-5.45627	-3.8176
H	-7.13734	-4.25485	-2.27623
H	-2.8551	-4.06807	-2.52494
H	-2.88927	-5.6475	-4.40735
H	-7.18006	-5.84498	-4.17494
H	-5.05268	-6.55071	-5.25268
C	-6.28334	-1.35814	-2.3982
C	-7.42741	-0.31145	-4.74695
C	-7.58247	-0.82273	-2.3882
C	-5.56794	-1.36262	-3.60667
C	-6.13474	-0.84099	-4.76871
C	-8.15004	-0.3049	-3.55224
H	-8.14199	-0.81927	-1.46066
H	-4.57051	-1.78089	-3.63478
H	-5.56647	-0.84644	-5.6918
H	-9.15422	0.10252	-3.52618
H	-7.86612	0.09248	-5.65205
C	1.05188	-5.17295	-2.63737
C	-0.15155	-5.52297	-5.15211
C	1.26879	-6.34539	-3.3734
C	0.20797	-4.18569	-3.16453
C	-0.3839	-4.35615	-4.41813
C	0.66902	-6.52096	-4.62175
H	1.92242	-7.10935	-2.96975
H	0.02418	-3.28339	-2.5945
H	-1.02346	-3.57962	-4.82221
H	0.85038	-7.43029	-5.18321
H	-0.60249	-5.64977	-6.12996
C	3.82804	-4.33018	-2.61551
C	5.09152	-4.2364	-5.12832
C	4.92578	-5.15999	-2.89906

C	3.38736	-3.43622	-3.60201
C	4.00508	-3.40071	-4.85351
C	5.559	-5.10742	-4.14218
H	5.25945	-5.8642	-2.14524
H	2.53924	-2.79621	-3.40106
H	3.63008	-2.72885	-5.61777
H	6.40065	-5.75923	-4.34684
H	5.56405	-4.21286	-6.10368
C	3.9967	-4.82379	2.34986
C	5.61214	-4.81054	4.65446
C	5.32049	-5.29188	2.30278
C	3.49446	-4.35133	3.57315
C	4.2974	-4.34151	4.71285
C	6.12094	-5.28744	3.44492
H	5.71412	-5.66124	1.36356
H	2.47297	-3.99922	3.63065
H	3.89629	-3.96704	5.64763
H	7.13947	-5.65443	3.39001
H	6.23337	-4.80319	5.5426
C	1.24844	-5.97712	2.29568
C	0.15041	-7.28442	4.53017
C	1.83985	-7.14573	2.80371
C	0.09893	-5.47372	2.92275
C	-0.44462	-6.12404	4.03293
C	1.29578	-7.79349	3.91204
H	2.73097	-7.53591	2.32733
H	-0.37997	-4.58296	2.54077
H	-1.34166	-5.7242	4.48967
H	1.76416	-8.69479	4.29101
H	-0.27482	-7.79051	5.38952
C	4.43108	3.52767	-2.64556
C	3.7563	4.71961	-5.10243
C	3.64818	2.83681	-3.58218
C	4.85567	4.83053	-2.94998
C	4.52264	5.42218	-4.16904
C	3.31389	3.42789	-4.801
H	3.3112	1.83355	-3.35725
H	5.45975	5.36841	-2.22883
H	4.86711	6.42523	-4.39322
H	2.71463	2.87903	-5.51851
H	3.51192	5.17013	-6.05812
C	6.04641	1.07714	-2.56734
C	7.1715	-0.00352	-4.90296
C	5.48277	-0.05603	-3.16521

C	7.17813	1.66492	-3.15469
C	7.73431	1.1329	-4.31806
C	6.0482	-0.59505	-4.32272
H	4.62232	-0.53476	-2.7155
H	7.61473	2.54259	-2.69291
H	8.60618	1.60047	-4.76135
H	5.61823	-1.49191	-4.74703
H	7.60746	-0.42735	-5.80053
C	6.28326	1.35845	2.39783
C	7.42733	0.31189	4.74664
C	5.56785	1.36297	3.60628
C	7.5824	0.82306	2.38786
C	8.14996	0.3053	3.55194
C	6.13464	0.84141	4.76836
H	4.57041	1.78121	3.63436
H	8.14192	0.81956	1.46033
H	9.15416	-0.1021	3.52591
H	5.56637	0.84689	5.69144
H	7.86604	-0.09198	5.65177
C	4.99647	4.04781	2.26724
C	5.03847	5.85234	4.42343
C	3.80237	4.44683	2.884
C	6.21238	4.56375	2.74668
C	6.23368	5.45651	3.81698
C	3.82595	5.3448	3.95445
H	2.85527	4.06906	2.52429
H	7.13756	4.25471	2.27577
H	7.1806	5.84502	4.17432
H	2.88975	5.64865	4.40656
H	5.05335	6.5514	5.2519
H	-0.06405	2.9692	-0.17986

Zero-point correction= 1.931339 (Hartree/Particle)
Thermal correction to Energy= 2.044732
Thermal correction to Enthalpy= 2.045676
Thermal correction to Gibbs Free Energy= 1.764975
Sum of electronic and zero-point Energies= -5543.278889
Sum of electronic and thermal Energies= -5543.165497
Sum of electronic and thermal Enthalpies= -5543.164552
Sum of electronic and thermal Free Energies= -5543.445253

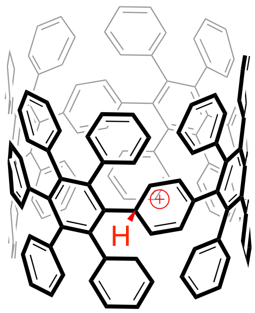

27b

Charge = 1 Multiplicity = 1

C	-5.67492	-1.47009	0.95287
C	-3.48602	-3.25307	0.81657
C	-5.83463	-2.74367	0.35219
C	-4.49562	-1.18533	1.67824
C	-3.39909	-2.07633	1.5919
C	-4.73591	-3.63651	0.28771
C	-6.76304	-0.36092	0.63156
C	-5.70674	2.07206	-0.57736
C	-6.63455	0.92924	1.36501
C	-6.71899	-0.12626	-0.84532
C	-6.21736	1.00309	-1.4001
C	-6.16306	2.06788	0.78623
H	-6.90253	0.95172	2.41333
H	-7.06048	-0.92959	-1.48684
H	-6.17232	1.08919	-2.47464
H	-6.00813	2.94006	1.40394
C	-2.21648	-4.00598	0.53772
C	0.44986	-4.96312	0.21369
C	-1.76335	-5.13011	1.23747
C	-1.3589	-3.4525	-0.4244
C	-0.06284	-3.91681	-0.58035
C	-0.45539	-5.60651	1.07303
H	-2.41354	-5.61608	1.95863
H	-1.69109	-2.6016	-1.01373
H	0.59509	-3.40931	-1.27567
H	-0.1296	-6.44984	1.67028
C	-4.64903	2.98873	-1.00107
C	-2.07615	4.13894	-0.86715
C	-4.46415	4.26979	-0.3735
C	-3.59023	2.46088	-1.81594
C	-2.29931	3.00381	-1.68578
C	-3.17965	4.8468	-0.32597
C	1.95101	-5.04468	0.22437

C	4.34463	-3.61135	0.1569
C	2.66753	-4.94299	-0.99897
C	2.64885	-4.81471	1.43724
C	3.84928	-4.07518	1.39679
C	3.8835	-4.22168	-1.03362
C	-0.65426	4.50676	-0.56594
C	2.16818	4.63385	-0.19186
C	-0.0253	3.76272	0.44374
C	0.12704	5.41347	-1.2905
C	1.5139	5.48307	-1.10008
C	1.34655	3.82862	0.62512
H	-0.6077	3.07497	1.05171
H	-0.33236	6.03892	-2.04981
H	2.08802	6.16534	-1.71494
H	1.80532	3.18244	1.36346
C	4.22763	3.79314	-1.37114
C	5.1478	2.83934	1.11943
C	3.62086	4.24722	-0.17183
C	5.10207	2.68713	-1.30986
C	5.38393	2.09147	-0.06141
C	4.24609	3.9262	1.0648
C	4.92053	-2.21659	0.12271
C	5.4328	0.5856	0.01598
C	3.88636	-1.27394	0.27759
C	6.2149	-1.72723	-0.07782
C	6.46841	-0.34193	-0.1304
C	4.13403	0.08265	0.22208
H	2.87078	-1.62662	0.4309
H	7.04151	-2.41863	-0.2063
H	7.4846	0.00191	-0.29383
H	3.31014	0.78228	0.32778
C	3.86714	4.71669	2.27662
C	3.14263	6.29214	4.50179
C	3.50504	4.12033	3.49643
C	3.85513	6.11996	2.19845
C	3.50021	6.90003	3.29827
C	3.14488	4.89885	4.59441
H	3.50956	3.03971	3.58674
H	4.14067	6.59878	1.2669
H	3.5094	7.98296	3.21489
H	2.86895	4.41503	5.52703
H	2.87036	6.89726	5.36158
C	5.89054	2.49262	2.37782
C	7.42465	1.94652	4.67828

C	6.81645	3.42888	2.86856
C	5.74813	1.27649	3.06299
C	6.50889	1.00865	4.20266
C	7.57479	3.16063	4.00625
H	6.9407	4.37341	2.34769
H	5.04318	0.53218	2.71227
H	6.38443	0.05637	4.70931
H	8.28635	3.89919	4.36389
H	8.01789	1.73383	5.56277
C	3.93355	4.46387	-2.68095
C	3.47905	5.81789	-5.10635
C	4.70715	5.57919	-3.03725
C	2.92415	4.04279	-3.55596
C	2.6995	4.71415	-4.75766
C	4.48514	6.24944	-4.24114
H	5.49144	5.91694	-2.36607
H	2.29934	3.19866	-3.28849
H	1.91125	4.3716	-5.42187
H	5.09877	7.10692	-4.5014
H	3.30503	6.33732	-6.04409
C	5.6976	2.09035	-2.54781
C	6.89878	1.04627	-4.87016
C	7.08211	2.19619	-2.75199
C	4.92806	1.43357	-3.51807
C	5.52245	0.9206	-4.67117
C	7.67851	1.68131	-3.90324
H	7.68892	2.70146	-2.00638
H	3.85922	1.32725	-3.36875
H	4.90868	0.42663	-5.41941
H	8.75041	1.78129	-4.04605
H	7.35903	0.65333	-5.7719
C	-2.95488	6.21851	0.23745
C	-2.5272	8.81379	1.22483
C	-3.46699	7.33431	-0.44086
C	-2.21789	6.42167	1.41285
C	-2.0108	7.71026	1.90514
C	-3.25314	8.62235	0.04848
H	-4.03533	7.19145	-1.35507
H	-1.79895	5.57192	1.9417
H	-1.43888	7.85001	2.81735
H	-3.65304	9.47552	-0.49102
H	-2.36127	9.81653	1.60637
C	-1.17701	2.35633	-2.4501
C	0.75334	0.982	-3.96221

C	-1.00016	2.64188	-3.81063
C	-0.35483	1.39189	-1.8521
C	0.60529	0.71034	-2.60074
C	-0.0463	1.95599	-4.56281
H	-1.62726	3.38908	-4.28737
H	-0.4717	1.16867	-0.79653
H	1.23391	-0.03469	-2.12182
H	0.06556	2.17675	-5.62007
H	1.48768	0.44021	-4.55038
C	-3.78947	1.34655	-2.79835
C	-4.15266	-0.65894	-4.73149
C	-4.34393	1.65341	-4.05128
C	-3.40874	0.02517	-2.53199
C	-3.59355	-0.97045	-3.49089
C	-4.5246	0.65744	-5.01123
H	-4.63222	2.67735	-4.27226
H	-2.9784	-0.22666	-1.56837
H	-3.29327	-1.99042	-3.27038
H	-4.95109	0.91094	-5.97698
H	-4.29045	-1.43473	-5.47824
C	-5.66939	4.9813	0.13595
C	-8.00944	6.26279	1.03792
C	-6.81627	5.05039	-0.67735
C	-5.7176	5.57502	1.41103
C	-6.87853	6.19905	1.85805
C	-7.97279	5.69127	-0.233
H	-6.78907	4.62435	-1.67539
H	-4.8477	5.53499	2.05597
H	-6.90134	6.64024	2.84972
H	-8.84112	5.74589	-0.88228
H	-8.90884	6.75974	1.38787
C	-4.39192	-0.04983	2.65365
C	-4.17463	1.97066	4.60334
C	-5.09692	-0.13812	3.86612
C	-3.55591	1.05788	2.44865
C	-3.4523	2.06036	3.41018
C	-4.99585	0.86559	4.83047
H	-5.72474	-1.00544	4.05131
H	-2.99495	1.13784	1.5235
H	-2.80253	2.91228	3.23243
H	-5.54936	0.77856	5.76058
H	-4.08847	2.75033	5.35391
C	-2.16583	-1.77209	2.40014
C	0.02504	-1.0502	4.0148

C	-2.1393	-2.10076	3.76274
C	-1.06468	-1.09846	1.85439
C	0.02193	-0.73997	2.65369
C	-1.05701	-1.73929	4.56517
H	-2.98224	-2.62708	4.20026
H	-1.05889	-0.85038	0.79781
H	0.8645	-0.21346	2.21438
H	-1.06313	-1.98938	5.62194
H	0.86417	-0.76071	4.64001
C	-4.89221	-4.99914	-0.31864
C	-5.17654	-7.57837	-1.40053
C	-5.66756	-5.96807	0.33504
C	-4.25697	-5.34444	-1.51985
C	-4.40147	-6.62279	-2.05858
C	-5.80731	-7.24794	-0.20037
H	-6.16243	-5.71587	1.268
H	-3.63935	-4.61287	-2.03088
H	-3.90204	-6.87271	-2.98981
H	-6.40809	-7.98673	0.32155
H	-5.28547	-8.57467	-1.81811
C	-7.16481	-3.16382	-0.20072
C	-9.68319	-3.96273	-1.17671
C	-8.26356	-3.29783	0.66426
C	-7.3501	-3.44679	-1.56346
C	-8.59819	-3.83926	-2.04665
C	-9.51165	-3.69428	0.18161
H	-8.12966	-3.11319	1.72718
H	-6.50837	-3.36698	-2.24425
H	-8.72115	-4.05533	-3.1036
H	-10.34569	-3.80158	0.86859
H	-10.65255	-4.27348	-1.55372
C	4.69538	-4.11427	-2.2906
C	6.30116	-4.04048	-4.60325
C	4.93101	-2.90458	-2.96007
C	5.28152	-5.28484	-2.79995
C	6.07478	-5.25015	-3.94502
C	5.72705	-2.87061	-4.10644
H	4.49601	-1.98362	-2.5878
H	5.10979	-6.2279	-2.29008
H	6.51872	-6.16796	-4.31946
H	5.90445	-1.92068	-4.60043
H	6.92263	-4.01008	-5.49342
C	2.06595	-5.55268	-2.22577
C	0.8983	-6.78191	-4.48031

C	1.91159	-4.84977	-3.43222
C	1.61942	-6.88414	-2.17548
C	1.04495	-7.49417	-3.28991
C	1.33243	-5.45644	-4.54495
H	2.25075	-3.82199	-3.50033
H	1.73628	-7.44535	-1.25348
H	0.71659	-8.52764	-3.22768
H	1.22341	-4.8925	-5.46702
H	0.45487	-7.2557	-5.35121
C	2.12001	-5.32635	2.74586
C	1.20348	-6.38765	5.18551
C	1.2151	-4.61109	3.54081
C	2.55343	-6.58485	3.18919
C	2.1015	-7.11117	4.40021
C	0.76203	-5.13729	4.75031
H	0.85352	-3.64523	3.20637
H	3.25364	-7.14994	2.58091
H	2.45237	-8.08519	4.72846
H	0.06084	-4.56604	5.3516
H	0.85163	-6.79501	6.1288
C	4.59666	-3.7063	2.6414
C	6.08329	-3.07404	4.94875
C	4.05506	-2.88439	3.64032
C	5.90293	-4.19113	2.81306
C	6.63913	-3.88213	3.95649
C	4.79054	-2.57248	4.7838
H	3.05365	-2.48635	3.52008
H	6.33521	-4.82585	2.0452
H	7.64486	-4.27512	4.07268
H	4.35116	-1.94038	5.55048
H	6.65128	-2.83863	5.84404
H	-7.7406	-0.80296	0.87464

Zero-point correction= 1.943208 (Hartree/Particle)
Thermal correction to Energy= 2.056632
Thermal correction to Enthalpy= 2.057576
Thermal correction to Gibbs Free Energy= 1.777841
Sum of electronic and zero-point Energies= -5543.637155
Sum of electronic and thermal Energies= -5543.523730
Sum of electronic and thermal Enthalpies= -5543.522786
Sum of electronic and thermal Free Energies= -5543.802522

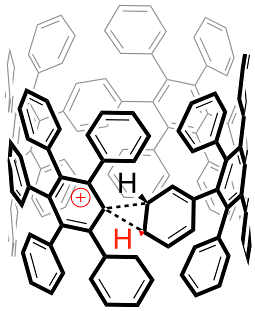

27c

Charge = 1 Multiplicity = 1

C	5.55448	1.17054	-1.16536
C	4.94872	3.06423	0.87125
C	6.00207	0.97391	0.17971
C	4.55703	2.13762	-1.35634
C	4.12973	2.96976	-0.27828
C	5.89512	2.04221	1.11981
C	7.2228	-0.55647	0.38303
C	5.13834	-2.51156	-0.08851
C	7.35194	-1.54555	-0.64714
C	5.96782	-0.496	1.06871
C	4.93475	-1.4679	0.77793
C	6.38172	-2.50167	-0.83017
H	8.26503	-1.58659	-1.22923
H	4.01859	-1.40547	1.35184
H	6.5615	-3.28957	-1.55165
H	8.09634	-0.07219	0.80007
H	5.96665	-0.14238	2.09379
C	2.77877	3.60205	-0.35049
C	0.07224	4.46362	-0.28696
C	1.72125	2.67783	-0.41523
C	2.45816	4.96517	-0.27742
C	1.12259	5.38854	-0.25677
C	0.40322	3.10022	-0.37408
H	1.94146	1.61599	-0.46269
H	3.24971	5.70553	-0.23149
H	0.90373	6.45008	-0.20499
H	-0.39673	2.36666	-0.40604
C	4.04743	-3.54716	-0.14307
C	1.40632	-4.49282	0.04139
C	3.32368	-3.81837	-1.32976
C	3.55888	-4.05073	1.09506
C	2.24304	-4.55724	1.17988
C	1.98288	-4.25992	-1.22507

C	-1.40813	4.71749	-0.21311
C	-4.10655	3.96357	-0.16502
C	-2.18238	4.86489	-1.38694
C	-2.06243	4.55031	1.02492
C	-3.43473	4.22258	1.05783
C	-3.54514	4.48805	-1.36337
C	-5.08802	2.82553	-0.24068
C	-6.10605	0.14464	-0.05404
C	-6.12341	2.49246	0.64912
C	-4.70952	1.81907	-1.15255
C	-5.20029	0.5313	-1.06468
C	-6.62251	1.18424	0.73926
H	-6.516	3.23542	1.33241
H	-3.93127	2.0217	-1.87799
H	-4.78374	-0.22046	-1.72296
H	-7.37704	0.97598	1.4878
C	-0.08952	-4.4475	0.18881
C	-2.88232	-3.96345	0.44188
C	-1.00082	-5.50731	0.24693
C	-0.59524	-3.14043	0.27804
C	-1.95435	-2.90627	0.40094
C	-2.37963	-5.26801	0.3698
H	-0.64344	-6.53083	0.19172
H	0.09329	-2.30022	0.2444
H	-2.32084	-1.88525	0.44317
H	-3.06211	-6.11124	0.39532
C	-6.07902	-1.33111	0.23287
C	-4.30233	-3.46677	0.4819
C	-5.95733	-2.26092	-0.83625
C	-5.7037	-1.76461	1.53445
C	-4.7518	-2.79699	1.6432
C	-5.06742	-3.35276	-0.70425
C	4.01254	2.4072	-2.73029
C	3.04925	2.94408	-5.31399
C	4.47133	3.52181	-3.44506
C	3.05536	1.57241	-3.32237
C	2.57349	1.84051	-4.6035
C	3.99782	3.78511	-4.73034
H	5.21068	4.17833	-2.99629
H	2.68033	0.71346	-2.77401
H	1.82907	1.18643	-5.04776
H	4.37026	4.6476	-5.2744
H	2.68343	3.14633	-6.31609
C	6.67045	2.0131	2.39664

C	8.1838	1.95771	4.76459
C	8.07309	2.08785	2.35359
C	6.03791	1.91386	3.64745
C	6.7909	1.87939	4.81987
C	8.82332	2.06745	3.52926
H	8.57372	2.2	1.3957
H	4.955	1.87131	3.69888
H	6.28787	1.79977	5.77844
H	9.90515	2.14294	3.47952
H	8.76698	1.93934	5.67985
C	4.81521	4.20736	1.83099
C	4.64807	6.39812	3.57846
C	5.85416	5.14801	1.89923
C	3.68864	4.38058	2.64676
C	3.60902	5.46861	3.51577
C	5.77145	6.23644	2.76668
H	6.72849	5.02643	1.26657
H	2.86447	3.6765	2.5962
H	2.72403	5.59154	4.13116
H	6.58236	6.95749	2.80449
H	4.58058	7.24632	4.25285
C	-1.55631	5.46376	-2.61264
C	-0.40947	6.70223	-4.86762
C	-1.96639	6.74374	-3.01859
C	-0.55928	4.81644	-3.35666
C	0.0071	5.43044	-4.47479
C	-1.39986	7.35762	-4.1343
H	-2.73977	7.25562	-2.45415
H	-0.2247	3.82617	-3.0661
H	0.77862	4.91016	-5.03441
H	-1.7331	8.34807	-4.43013
H	0.03197	7.17905	-5.73787
C	-4.41608	4.58219	-2.57601
C	-6.13642	4.79586	-4.80003
C	-5.6763	5.1929	-2.46089
C	-4.038	4.07884	-3.83167
C	-4.88959	4.18151	-4.92949
C	-6.52659	5.30188	-3.56011
H	-5.98466	5.59056	-1.49894
H	-3.07299	3.59825	-3.94869
H	-4.57875	3.77833	-5.88896
H	-7.49382	5.78254	-3.44645
H	-6.79763	4.8779	-5.65748
C	-1.3007	4.7279	2.30892

C	0.01131	5.10006	4.77322
C	-1.33849	5.96727	2.9633
C	-0.57563	3.68343	2.90009
C	0.07168	3.86537	4.12301
C	-0.69016	6.15335	4.18458
H	-1.89741	6.78437	2.51739
H	-0.53502	2.71707	2.40719
H	0.6081	3.03678	4.57782
H	-0.7434	7.11787	4.68088
H	0.49675	5.23615	5.73553
C	-4.15763	4.14987	2.36514
C	-5.47664	4.22675	4.85186
C	-5.13957	5.11864	2.63174
C	-3.85941	3.21221	3.36276
C	-4.50736	3.25576	4.5964
C	-5.79569	5.1568	3.86215
H	-5.37601	5.85445	1.86845
H	-3.11132	2.4505	3.17368
H	-4.24857	2.53213	5.36348
H	-6.54864	5.91688	4.0488
H	-5.978	4.25899	5.81453
C	-6.73631	-2.00475	-2.08676
C	-8.29217	-1.4789	-4.38119
C	-8.10137	-1.68669	-1.98102
C	-6.1683	-2.05054	-3.37121
C	-6.93637	-1.78853	-4.50355
C	-8.87194	-1.42891	-3.11368
H	-8.55963	-1.65024	-0.99768
H	-5.11644	-2.28777	-3.4846
H	-6.4732	-1.8247	-5.48543
H	-9.92584	-1.19035	-3.00423
H	-8.8899	-1.27845	-5.26538
C	-4.95694	-4.40108	-1.77119
C	-4.86727	-6.44412	-3.70778
C	-6.06494	-5.23587	-1.99152
C	-3.80208	-4.60996	-2.53809
C	-3.75971	-5.62272	-3.49795
C	-6.02263	-6.24619	-2.95032
H	-6.96612	-5.08392	-1.40536
H	-2.93206	-3.97975	-2.38874
H	-2.85353	-5.76803	-4.07754
H	-6.89193	-6.87905	-3.10337
H	-4.8311	-7.23191	-4.45454
C	-4.21756	-3.21238	2.98515

C	-3.31561	-4.01605	5.52963
C	-4.80962	-4.29653	3.64884
C	-3.15288	-2.54953	3.61119
C	-2.70723	-2.94412	4.87344
C	-4.36536	-4.69489	4.90986
H	-5.63288	-4.82171	3.17391
H	-2.67509	-1.71459	3.10787
H	-1.8912	-2.40679	5.3492
H	-4.84402	-5.53181	5.40977
H	-2.97791	-4.31603	6.51748
C	-6.34491	-1.14991	2.73686
C	-7.71071	-0.14307	4.98636
C	-7.7237	-1.3703	2.90668
C	-5.66791	-0.40334	3.71037
C	-6.34499	0.09095	4.82489
C	-8.40136	-0.8744	4.01961
H	-8.25949	-1.94959	2.16067
H	-4.61071	-0.19907	3.5903
H	-5.80387	0.66625	5.5689
H	-9.46503	-1.06291	4.13195
H	-8.23227	0.24243	5.85734
C	3.94105	-3.69388	-2.68882
C	4.95721	-3.75004	-5.31769
C	3.46091	-2.80725	-3.66384
C	4.9516	-4.59955	-3.05467
C	5.45953	-4.62543	-4.35424
C	3.95896	-2.83891	-4.96668
H	2.6667	-2.11335	-3.41071
H	5.30947	-5.31738	-2.32138
H	6.22919	-5.34462	-4.61801
H	3.55797	-2.15708	-5.71089
H	5.3323	-3.78477	-6.33612
C	1.18252	-4.52344	-2.47028
C	-0.19556	-5.04544	-4.86569
C	1.35541	-5.73956	-3.14549
C	0.29126	-3.58111	-3.00162
C	-0.38957	-3.83778	-4.19207
C	0.67295	-5.99938	-4.33399
H	2.03791	-6.4811	-2.74108
H	0.13396	-2.64083	-2.48292
H	-1.07087	-3.09377	-4.59437
H	0.82333	-6.94541	-4.84541
H	-0.71863	-5.24102	-5.79704
C	1.75297	-5.21868	2.4346

C	0.88534	-6.58288	4.73664
C	2.3512	-6.42843	2.82236
C	0.7085	-4.70635	3.21692
C	0.28132	-5.38336	4.36001
C	1.92223	-7.10525	3.96245
H	3.15728	-6.83972	2.22236
H	0.21748	-3.78268	2.93078
H	-0.53776	-4.97553	4.94338
H	2.39567	-8.0417	4.24224
H	0.54668	-7.11013	5.62348
C	4.4573	-4.04638	2.2946
C	6.19909	-4.05923	4.50966
C	4.08848	-3.43415	3.50306
C	5.71449	-4.66726	2.22053
C	6.57648	-4.67718	3.31708
C	4.9516	-3.43766	4.59764
H	3.11518	-2.96178	3.58794
H	6.009	-5.16046	1.29858
H	7.53926	-5.17403	3.24112
H	4.64585	-2.96044	5.52409
H	6.8667	-4.06901	5.36585
C	6.33893	0.65235	-2.3328
C	7.96801	-0.05662	-4.51501
C	5.84173	-0.16107	-3.35442
C	7.66455	1.11835	-2.42895
C	8.47175	0.76981	-3.51043
C	6.65378	-0.51846	-4.43042
H	4.83126	-0.54263	-3.30245
H	8.05474	1.77541	-1.65756
H	9.48729	1.14977	-3.56936
H	6.25474	-1.16856	-5.20063
H	8.59156	-0.33404	-5.35931

Imaginary frequency = -313.0625

Zero-point correction= 1.941683 (Hartree/Particle)
Thermal correction to Energy= 2.054891
Thermal correction to Enthalpy= 2.055835
Thermal correction to Gibbs Free Energy= 1.777650
Sum of electronic and zero-point Energies= -5543.599551
Sum of electronic and thermal Energies= -5543.486344
Sum of electronic and thermal Enthalpies= -5543.485399
Sum of electronic and thermal Free Energies= -5543.763585

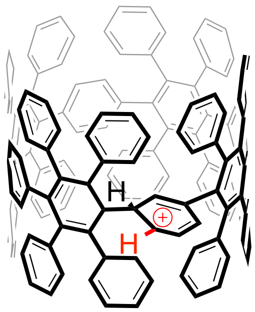

27d

Charge = 1 Multiplicity = 1

C	5.11019	2.50818	-0.07098
C	5.93375	-0.23857	-0.28321
C	6.1595	1.99598	0.70744
C	4.59322	1.65126	-1.06201
C	4.98548	0.33202	-1.1609
C	6.56739	0.65747	0.59959
H	6.63844	2.62214	1.45223
H	3.79158	2.00204	-1.70156
H	4.46932	-0.2981	-1.87264
H	7.35152	0.31038	1.26009
C	4.25251	3.71841	0.16265
C	1.67596	4.8127	0.17847
C	3.80686	4.47761	-0.95304
C	3.57772	3.87802	1.39838
C	2.27658	4.42033	1.40015
C	2.50277	5.02303	-0.94985
C	5.86344	-1.73913	-0.22065
C	3.97929	-3.78426	-0.07298
C	5.66997	-2.40622	1.02073
C	5.53778	-2.46576	-1.40574
C	4.5721	-3.49257	-1.32902
C	4.69352	-3.42588	1.09354
C	0.19006	4.63211	0.03845
C	-2.49988	3.81357	-0.40315
C	-0.78792	5.5482	-0.37148
C	-0.21849	3.30163	0.24955
C	-1.52588	2.90223	0.03427
C	-2.11522	5.14548	-0.5877
H	-0.51668	6.58393	-0.54588
H	0.51962	2.56833	0.55584
H	-1.80043	1.86185	0.18891
H	-2.84432	5.87967	-0.9161
C	2.51551	-4.12185	0.01391

C	-0.31565	-4.21709	0.3151
C	1.70204	-3.01405	-0.29715
C	1.8762	-5.28636	0.45522
C	0.48021	-5.33259	0.60175
C	0.3243	-3.05937	-0.15299
H	2.17469	-2.09499	-0.62963
H	2.46428	-6.16259	0.70713
H	0.01778	-6.24784	0.95858
H	-0.26721	-2.17773	-0.38572
C	-3.84572	3.18672	-0.63937
C	-5.85707	1.24948	-0.79616
C	-4.10952	2.55361	-1.87495
C	-4.75498	3.03662	0.43091
C	-5.76391	2.04798	0.35981
C	-5.12704	1.57581	-1.95946
C	-6.50596	-0.15834	-0.72063
C	-6.91412	-2.38103	1.01952
C	-7.77088	-0.35863	0.00227
C	-5.46394	-1.16854	-0.42124
C	-5.64222	-2.25883	0.40304
C	-7.95393	-1.45519	0.81741
H	-8.59007	0.32117	-0.1961
H	-4.50746	-1.02746	-0.91307
H	-8.90668	-1.61364	1.31029
H	-7.10132	-3.23439	1.6622
C	-1.8063	-4.09717	0.48396
C	-4.46101	-3.17706	0.57291
C	-2.33599	-3.59003	1.69121
C	-2.66662	-4.32932	-0.61364
C	-4.00192	-3.87726	-0.56639
C	-3.67252	-3.12709	1.74029
C	-4.24238	-2.59838	3.02081
C	-5.2466	-1.74783	5.51172
C	-3.78031	-1.4136	3.61317
C	-5.22526	-3.34216	3.69523
C	-5.72539	-2.92111	4.92909
C	-4.27398	-0.9947	4.84868
H	-3.01014	-0.83288	3.11665
H	-5.56177	-4.28407	3.26994
H	-6.47133	-3.52077	5.44213
H	-3.89145	-0.0836	5.29907
H	-5.61679	-1.42878	6.4814
C	-4.97942	-4.16204	-1.66614
C	-6.90674	-4.72785	-3.63928

C	-4.87619	-3.6041	-2.94759
C	-6.06704	-5.00729	-1.39043
C	-7.01945	-5.29344	-2.36822
C	-5.83454	-3.88172	-3.92345
H	-4.04366	-2.95099	-3.18463
H	-6.15003	-5.46005	-0.40617
H	-7.8449	-5.96093	-2.13896
H	-5.7421	-3.43173	-4.90652
H	-7.64626	-4.94846	-4.40295
C	-2.17061	-5.05257	-1.83143
C	-1.32575	-6.42644	-4.13264
C	-1.52153	-4.37215	-2.87033
C	-2.38215	-6.43196	-1.95727
C	-1.95971	-7.11495	-3.09813
C	-1.10762	-5.05274	-4.0155
H	-1.34102	-3.30494	-2.78156
H	-2.8844	-6.9703	-1.15887
H	-2.12959	-8.18431	-3.17914
H	-0.61735	-4.50826	-4.81734
H	-1.00295	-6.95664	-5.02338
C	-1.50378	-3.51849	2.93878
C	-0.12005	-3.32045	5.37821
C	-0.5956	-2.47408	3.16143
C	-1.68901	-4.47511	3.94624
C	-1.00243	-4.37851	5.15657
C	0.08781	-2.37319	4.37341
H	-0.43099	-1.7321	2.38648
H	-2.3863	-5.29191	3.78492
H	-1.1632	-5.12566	5.92796
H	0.77855	-1.55093	4.53538
H	0.40329	-3.23434	6.32571
C	4.45311	-4.1794	2.36964
C	4.11517	-5.70187	4.71347
C	5.4289	-5.10439	2.7759
C	3.30261	-4.03417	3.1555
C	3.13683	-4.79021	4.31755
C	5.26477	-5.8565	3.93751
H	6.32363	-5.22786	2.17343
H	2.53142	-3.33047	2.8603
H	2.23549	-4.66537	4.90853
H	6.03354	-6.56441	4.23337
H	3.98303	-6.28821	5.61813
C	6.46311	-2.01145	2.22128
C	8.07424	-1.39462	4.45063

C	7.86582	-2.06944	2.1403
C	5.8852	-1.63368	3.44248
C	6.68314	-1.33224	4.54528
C	8.66474	-1.76324	3.24142
H	8.32662	-2.37234	1.20476
H	4.80542	-1.58566	3.52926
H	6.21741	-1.05179	5.48557
H	9.74594	-1.81992	3.156
H	8.69193	-1.16208	5.31287
C	6.18688	-2.06257	-2.69022
C	7.50973	-1.28536	-5.05927
C	7.57167	-1.82061	-2.70341
C	5.47993	-1.90313	-3.89482
C	6.13287	-1.51754	-5.06309
C	8.22687	-1.43859	-3.87297
H	8.13716	-1.94326	-1.78514
H	4.40993	-2.07717	-3.91562
H	5.56336	-1.39607	-5.98005
H	9.29849	-1.26284	-3.8561
H	8.01735	-0.98805	-5.97204
C	4.25908	-4.3698	-2.50843
C	3.8285	-6.13875	-4.66367
C	5.29889	-5.17486	-3.00609
C	2.99726	-4.47292	-3.11152
C	2.78742	-5.3491	-4.17841
C	5.08892	-6.04731	-4.0712
H	6.28101	-5.10992	-2.54845
H	2.17299	-3.86822	-2.75272
H	1.79975	-5.41618	-4.62412
H	5.91001	-6.65806	-4.43502
H	3.66066	-6.81956	-5.49299
C	4.73501	4.63154	-2.11685
C	6.57305	4.94186	-4.23193
C	6.03702	5.10452	-1.88122
C	4.37538	4.31431	-3.4363
C	5.28507	4.46508	-4.48111
C	6.94572	5.26216	-2.92642
H	6.33126	5.35659	-0.86695
H	3.37923	3.94128	-3.64629
H	4.98721	4.20646	-5.49315
H	7.94472	5.63462	-2.71943
H	7.27943	5.06148	-5.04799
C	1.99964	5.82749	-2.1126
C	1.1005	7.43214	-4.24274

C	2.50143	7.12508	-2.29663
C	1.03676	5.34895	-3.01165
C	0.5918	6.14473	-4.06798
C	2.05794	7.9205	-3.35266
H	3.24966	7.5064	-1.6083
H	0.6294	4.35145	-2.88331
H	-0.15791	5.75541	-4.75
H	2.46116	8.92102	-3.47882
H	0.75372	8.05038	-5.06559
C	1.49782	4.5925	2.67003
C	0.0702	5.03544	5.06058
C	1.10719	5.88682	3.04996
C	1.14254	3.5225	3.50486
C	0.43686	3.74071	4.68758
C	0.40388	6.10865	4.23334
H	1.37365	6.7257	2.41408
H	1.42694	2.51296	3.23033
H	0.18281	2.89817	5.32496
H	0.12423	7.12045	4.51197
H	-0.46234	5.20695	5.99185
C	4.2235	3.47911	2.69284
C	5.41235	2.8646	5.16652
C	4.89122	4.46556	3.43301
C	4.1727	2.17611	3.20474
C	4.75721	1.87377	4.43402
C	5.48159	4.16243	4.66043
H	4.9392	5.47844	3.04409
H	3.68222	1.39488	2.6319
H	4.70823	0.85971	4.81591
H	5.99241	4.94084	5.21963
H	5.86852	2.62566	6.12264
C	-5.43787	0.88899	-3.25337
C	-6.09422	-0.32714	-5.71003
C	-4.48586	0.13588	-3.95905
C	-6.73015	1.00962	-3.79949
C	-7.05617	0.40484	-5.01466
C	-4.80932	-0.45907	-5.17768
H	-3.48058	0.03852	-3.56436
H	-7.47009	1.61401	-3.28137
H	-8.05449	0.52353	-5.42468
H	-4.05304	-1.02174	-5.71686
H	-6.33981	-0.78359	-6.66391
C	-3.33304	2.92402	-3.10616
C	-1.98321	3.56725	-5.4873

C	-2.0404	2.43807	-3.34707
C	-3.93192	3.75	-4.06749
C	-3.26179	4.07215	-5.24793
C	-1.37345	2.75193	-4.53158
H	-1.55618	1.80978	-2.60666
H	-4.93137	4.13658	-3.89106
H	-3.7418	4.7127	-5.9816
H	-0.37727	2.35741	-4.70902
H	-1.46553	3.80558	-6.41153
C	-4.6568	3.92468	1.63675
C	-4.52379	5.65385	3.84806
C	-3.66033	3.76397	2.60855
C	-5.58474	4.96575	1.78853
C	-5.51986	5.82366	2.88587
C	-3.59433	4.62329	3.70542
H	-2.9213	2.97747	2.50064
H	-6.35527	5.10935	1.0366
H	-6.24325	6.62762	2.98413
H	-2.80219	4.49493	4.43607
H	-4.46844	6.32487	4.69994
C	-6.71019	1.84515	1.50174
C	-8.53793	1.50228	3.61134
C	-6.32742	1.18169	2.67563
C	-8.02113	2.35246	1.41094
C	-8.92731	2.18442	2.45782
C	-7.2373	1.0028	3.71628
H	-5.31867	0.79622	2.77159
H	-8.31847	2.89481	0.51739
H	-9.93061	2.59132	2.37486
H	-6.92598	0.4741	4.61098
H	-9.23983	1.37052	4.42923
H	-6.77784	-0.38723	-1.78412

Zero-point correction= 1.941409 (Hartree/Particle)
Thermal correction to Energy= 2.055362
Thermal correction to Enthalpy= 2.056306
Thermal correction to Gibbs Free Energy= 1.774428
Sum of electronic and zero-point Energies= -5543.644913
Sum of electronic and thermal Energies= -5543.530959
Sum of electronic and thermal Enthalpies= -5543.530015
Sum of electronic and thermal Free Energies= -5543.811893

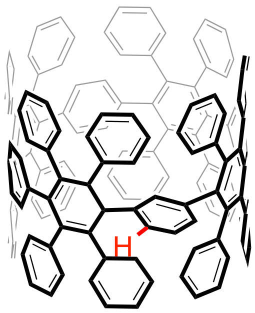

27e

Charge = 0 Multiplicity = 1

C	-5.38648	-1.07825	-0.06884
C	-5.15915	1.75571	0.16122
C	-6.49847	-0.24816	-0.2449
C	-4.15895	-0.45974	0.23619
C	-4.0496	0.91133	0.35185
C	-6.38592	1.15145	-0.13168
H	-7.46499	-0.68073	-0.48235
H	-3.27543	-1.07702	0.36596
H	-3.08234	1.35283	0.57092
H	-7.26745	1.76467	-0.29039
C	-5.19034	-2.56679	-0.21373
C	-3.2203	-4.5399	-0.40156
C	-4.85325	-3.09024	-1.48105
C	-4.87745	-3.33308	0.93738
C	-3.87326	-4.3223	0.84362
C	-3.88094	-4.11049	-1.58032
C	-4.7315	3.19999	0.25093
C	-2.51036	4.88856	0.43133
C	-4.32746	3.68656	1.51537
C	-4.30276	3.88865	-0.90814
C	-3.17628	4.7381	-0.81605
C	-3.22652	4.56427	1.6117
C	-1.74238	-4.82086	-0.41955
C	1.09585	-4.57648	-0.67223
C	-1.01665	-5.62819	-1.30984
C	-0.99135	-3.96572	0.4137
C	0.38421	-3.85421	0.29858
C	0.37379	-5.49604	-1.44238
H	-1.53507	-6.33493	-1.94713
H	-1.5079	-3.33088	1.12406
H	0.91575	-3.14882	0.93051
H	0.8871	-6.09709	-2.18719
C	-1.00868	4.98244	0.45276
C	1.7832	4.41461	0.69738

C	-0.37132	4.02033	-0.35774
C	-0.18793	5.71803	1.32241
C	1.17938	5.42857	1.45055
C	0.98236	3.75263	-0.24711
H	-0.96592	3.42984	-1.04468
H	-0.6174	6.49399	1.94542
H	1.76572	5.98353	2.17687
H	1.42297	2.97378	-0.86224
C	2.53408	-4.16659	-0.84491
C	4.92106	-2.66745	-0.67963
C	2.87445	-3.27046	-1.88024
C	3.48765	-4.46261	0.15651
C	4.68954	-3.7197	0.2352
C	4.07447	-2.5284	-1.8023
C	5.90478	-1.56845	-0.37989
C	7.5003	0.66339	0.28476
C	7.3006	-1.66965	-0.39113
C	5.33857	-0.3359	-0.02243
C	6.10688	0.78844	0.31495
C	8.08221	-0.55856	-0.06354
H	7.77615	-2.61082	-0.64827
H	4.25687	-0.24853	-0.00578
H	9.16477	-0.64659	-0.07978
H	8.12896	1.51483	0.52564
C	3.1681	3.84405	0.8535
C	5.31751	2.02867	0.63896
C	3.42745	2.93636	1.90236
C	4.11817	3.99224	-0.18383
C	5.20533	3.09171	-0.28544
C	4.50727	2.03091	1.79655
C	4.82529	1.09156	2.92269
C	5.4572	-0.54677	5.1277
C	3.95124	0.07584	3.33726
C	6.03053	1.2563	3.62255
C	6.34433	0.44822	4.71533
C	4.26208	-0.73343	4.42989
H	3.01781	-0.07551	2.80671
H	6.7187	2.03695	3.31234
H	7.27891	0.60131	5.2475
H	3.5672	-1.50906	4.73898
H	5.69256	-1.17084	5.98494
C	6.2515	3.28164	-1.34423
C	8.26425	3.69438	-3.2682
C	6.42344	2.38097	-2.40368

C	7.10781	4.39089	-1.2624
C	8.1061	4.59561	-2.21419
C	7.41822	2.58886	-3.35931
H	5.77679	1.51523	-2.48635
H	6.98451	5.09808	-0.44757
H	8.75853	5.46037	-2.13199
H	7.52683	1.87894	-4.17385
H	9.04012	3.85346	-4.01174
C	3.92119	5.0755	-1.1984
C	3.53765	7.1398	-3.07432
C	3.75337	4.78604	-2.5607
C	3.88871	6.41848	-0.79389
C	3.7027	7.44206	-1.72232
C	3.56129	5.80752	-3.48926
H	3.77518	3.75298	-2.89222
H	4.01838	6.65841	0.25728
H	3.68602	8.47564	-1.3883
H	3.42904	5.5618	-4.53906
H	3.39027	7.93564	-3.79861
C	2.55224	2.87386	3.12079
C	1.06267	2.5794	5.49019
C	1.34573	2.15977	3.12269
C	2.98331	3.46355	4.31688
C	2.24484	3.32081	5.492
C	0.61251	2.00457	4.2993
H	0.99273	1.70951	2.19989
H	3.91717	4.01765	4.32838
H	2.60191	3.77602	6.41136
H	-0.30574	1.42367	4.28954
H	0.49804	2.446	6.40846
C	-2.84346	5.1536	2.93874
C	-2.23415	6.37255	5.4015
C	-3.53489	6.29796	3.36565
C	-1.83918	4.63372	3.76424
C	-1.53662	5.23815	4.98456
C	-3.23624	6.90159	4.5875
H	-4.31467	6.71206	2.73301
H	-1.27665	3.76368	3.44575
H	-0.74738	4.8203	5.60171
H	-3.78592	7.78479	4.90057
H	-1.99752	6.84157	6.35232
C	-5.04664	3.18943	2.73065
C	-6.47511	2.27987	4.98282
C	-6.42124	3.44183	2.86381

C	-4.40472	2.45981	3.74218
C	-5.11072	2.01008	4.85763
C	-7.12921	2.99539	3.9792
H	-6.931	4.00365	2.08672
H	-3.34674	2.24051	3.65161
H	-4.59306	1.45041	5.63161
H	-8.19089	3.20857	4.0646
H	-7.02264	1.93684	5.85604
C	-5.05222	3.72304	-2.19706
C	-6.55069	3.52808	-4.57369
C	-5.76045	4.82844	-2.69666
C	-5.1098	2.51679	-2.90997
C	-5.85321	2.42235	-4.08777
C	-6.50078	4.73429	-3.87331
H	-5.72335	5.76849	-2.15459
H	-4.57439	1.64655	-2.54667
H	-5.89069	1.4751	-4.61597
H	-7.04035	5.60285	-4.24031
H	-7.12975	3.45062	-5.48953
C	-2.62019	5.44973	-2.00943
C	-1.562	6.87608	-4.20007
C	-2.36014	6.8274	-1.91923
C	-2.3357	4.80194	-3.22253
C	-1.8104	5.50627	-4.30382
C	-1.83965	7.5345	-3.00221
H	-2.5765	7.3447	-0.98948
H	-2.52536	3.73859	-3.31904
H	-1.59287	4.98205	-5.23008
H	-1.65074	8.60009	-2.90811
H	-1.15451	7.42397	-5.04474
C	-5.48277	-2.49942	-2.70327
C	-6.73315	-1.46782	-5.00698
C	-6.86832	-2.62613	-2.88979
C	-4.73815	-1.82848	-3.68399
C	-5.35599	-1.32171	-4.82708
C	-7.48907	-2.11722	-4.03072
H	-7.45556	-3.14531	-2.13798
H	-3.66868	-1.70797	-3.55089
H	-4.75919	-0.81656	-5.58147
H	-8.5614	-2.23397	-4.15887
H	-7.212	-1.0794	-5.90122
C	-3.57278	-4.73251	-2.91199
C	-3.11858	-6.00011	-5.38389
C	-4.39361	-5.78687	-3.34159

C	-2.51808	-4.32803	-3.73907
C	-2.29268	-4.95669	-4.96386
C	-4.17195	-6.41412	-4.568
H	-5.21344	-6.11185	-2.70739
H	-1.85618	-3.53209	-3.4182
H	-1.4634	-4.62881	-5.58286
H	-4.82148	-7.22603	-4.88289
H	-2.94191	-6.48777	-6.33838
C	-3.42778	-5.11628	2.03071
C	-2.5882	-6.69155	4.214
C	-3.32174	-6.51267	1.918
C	-3.10014	-4.52623	3.26249
C	-2.68324	-5.30447	4.34029
C	-2.90983	-7.29302	2.99729
H	-3.5733	-6.98576	0.97372
H	-3.17053	-3.45	3.37619
H	-2.4302	-4.82479	5.28155
H	-2.84046	-8.37137	2.88589
H	-2.2656	-7.29693	5.05611
C	-5.65391	-3.11849	2.20573
C	-7.24426	-2.84135	4.51884
C	-6.48139	-4.16577	2.6464
C	-5.63979	-1.9287	2.94948
C	-6.42757	-1.79407	4.09434
C	-7.26667	-4.03118	3.78928
H	-6.50466	-5.09306	2.08215
H	-5.01196	-1.10149	2.64171
H	-6.40076	-0.85927	4.6463
H	-7.89872	-4.85577	4.10687
H	-7.85784	-2.73265	5.40864
C	4.47753	-1.62226	-2.92893
C	5.23099	-0.06043	-5.15169
C	3.74748	-0.4785	-3.28477
C	5.60483	-1.95463	-3.69579
C	5.9786	-1.18386	-4.79695
C	4.11729	0.29243	-4.38664
H	2.87777	-0.1958	-2.70205
H	6.18223	-2.83591	-3.43241
H	6.84946	-1.46712	-5.38135
H	3.53177	1.16869	-4.65001
H	5.51212	0.53554	-6.01508
C	1.96194	-3.03834	-3.04994
C	0.42264	-2.42479	-5.32472
C	0.87913	-2.15184	-2.96331

C	2.24075	-3.63578	-4.28641
C	1.47716	-3.33421	-5.41475
C	0.12188	-1.83964	-4.09244
H	0.64387	-1.69129	-2.00869
H	3.079	-4.32138	-4.36739
H	1.71752	-3.79792	-6.36725
H	-0.6957	-1.1283	-4.01272
H	-0.15921	-2.17167	-6.20624
C	3.17886	-5.53264	1.15763
C	2.57142	-7.56784	3.00601
C	3.0725	-5.25027	2.52745
C	2.9705	-6.85295	0.73153
C	2.67417	-7.86279	1.64626
C	2.76936	-6.25712	3.44246
H	3.22873	-4.23452	2.87604
H	3.04831	-7.08676	-0.32613
H	2.52163	-8.87948	1.29528
H	2.68725	-6.0166	4.49857
H	2.33756	-8.3524	3.71975
C	5.72816	-4.06853	1.26047
C	7.71579	-4.78395	3.12004
C	6.0458	-3.21979	2.32911
C	6.42516	-5.28056	1.13694
C	7.41096	-5.63543	2.05694
C	7.02873	-3.57686	3.25245
H	5.52365	-2.27661	2.44248
H	6.18771	-5.9484	0.31423
H	7.93948	-6.57771	1.94282
H	7.25393	-2.90327	4.0739
H	8.48257	-5.05955	3.83852

Zero-point correction= 1.931147 (Hartree/Particle)
Thermal correction to Energy= 2.044673
Thermal correction to Enthalpy= 2.045617
Thermal correction to Gibbs Free Energy= 1.764399
Sum of electronic and zero-point Energies= -5543.314139
Sum of electronic and thermal Energies= -5543.200614
Sum of electronic and thermal Enthalpies= -5543.199669
Sum of electronic and thermal Free Energies= -5543.480887

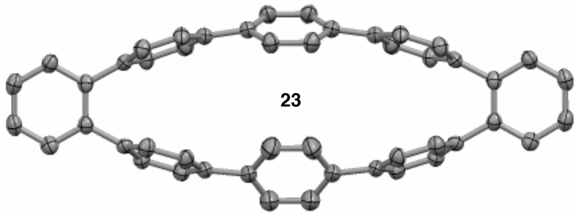

Charge = 0 Multiplicity = 1

H	1.21529	4.08375	-1.45731
C	0.69351	3.52389	-0.70132
C	-0.69133	2.1318	1.23057
C	1.41463	2.78524	0.23617
C	-0.69351	3.52389	-0.70132
C	-1.41463	2.78524	0.23617
C	0.69133	2.1318	1.23057
H	-1.21529	4.08375	-1.45731
H	1.20836	1.5403	1.96338
H	-1.20836	1.5403	1.96338
C	2.87861	2.52452	0.11653
C	5.48393	1.46485	-0.09699
C	3.43676	2.23078	-1.12554
C	3.69303	2.38613	1.23923
C	4.97144	1.86112	1.13399
C	4.71862	1.71468	-1.22952
H	2.84118	2.32715	-2.01529
H	3.31475	2.65094	2.2106
H	5.55641	1.71492	2.0251
H	5.09845	1.44289	-2.19836
C	6.77509	0.70305	-0.18845
C	9.19049	-0.69234	-0.33923
C	7.98801	1.37997	-0.26325
C	6.77509	-0.70305	-0.18845
C	7.98801	-1.37997	-0.26325
C	9.19049	0.69234	-0.33923
H	7.9867	2.45568	-0.26248
H	7.9867	-2.45568	-0.26248
H	10.11667	1.23605	-0.39793
H	10.11667	-1.23605	-0.39793
C	5.48393	-1.46485	-0.09699
C	2.87861	-2.52452	0.11653
C	4.97144	-1.86112	1.13399
C	4.71862	-1.71468	-1.22952
C	3.43676	-2.23078	-1.12554
C	3.69303	-2.38613	1.23923

H	5.55641	-1.71492	2.0251
H	5.09845	-1.44289	-2.19836
H	2.84118	-2.32715	-2.01529
H	3.31475	-2.65094	2.2106
C	1.41463	-2.78524	0.23617
C	-1.41463	-2.78524	0.23617
C	0.69133	-2.1318	1.23057
C	0.69351	-3.52389	-0.70132
C	-0.69351	-3.52389	-0.70132
C	-0.69133	-2.1318	1.23057
H	1.20836	-1.5403	1.96338
H	1.21529	-4.08375	-1.45731
H	-1.21529	-4.08375	-1.45731
H	-1.20836	-1.5403	1.96338
C	-2.87861	-2.52452	0.11653
C	-5.48393	-1.46485	-0.09699
C	-3.69303	-2.38613	1.23923
C	-3.43676	-2.23078	-1.12554
C	-4.71862	-1.71468	-1.22952
C	-4.97144	-1.86112	1.13399
H	-3.31475	-2.65094	2.2106
H	-2.84118	-2.32715	-2.01529
H	-5.09845	-1.44289	-2.19836
H	-5.55641	-1.71492	2.0251
C	-2.87861	2.52452	0.11653
C	-5.48393	1.46485	-0.09699
C	-3.69303	2.38613	1.23923
C	-3.43676	2.23078	-1.12554
C	-4.71862	1.71468	-1.22952
C	-4.97144	1.86112	1.13399
H	-3.31475	2.65094	2.2106
H	-2.84118	2.32715	-2.01529
H	-5.09845	1.44289	-2.19836
H	-5.55641	1.71492	2.0251
C	-6.77509	-0.70305	-0.18845
C	-9.19049	0.69234	-0.33923
C	-6.77509	0.70305	-0.18845
C	-7.98801	-1.37997	-0.26325
C	-9.19049	-0.69234	-0.33923
C	-7.98801	1.37997	-0.26325
H	-7.9867	-2.45568	-0.26248
H	-10.11667	-1.23605	-0.39793
H	-7.9867	2.45568	-0.26248
H	-10.11667	1.23605	-0.39793

Zero-point correction= 0.644125 (Hartree/Particle)
Thermal correction to Energy= 0.679749
Thermal correction to Enthalpy= 0.680693
Thermal correction to Gibbs Free Energy= 0.575672
Sum of electronic and zero-point Energies= -1847.880698
Sum of electronic and thermal Energies= -1847.845074
Sum of electronic and thermal Enthalpies= -1847.844130
Sum of electronic and thermal Free Energies= -1847.949151

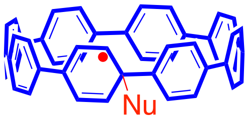

25

Nu = H

Charge = 0 Multiplicity = 2

H	1.97913	4.40604	-2.09818
C	2.5188	4.18461	-1.19634
C	3.94306	3.52035	1.09816
C	1.94415	4.48934	0.06132
C	3.71171	3.48001	-1.29984
C	4.42107	3.02776	-0.15013
C	2.74738	4.21916	1.19778
H	4.05461	3.22154	-2.28222
H	2.408	4.51654	2.17297
H	4.46551	3.28857	2.00578
C	0.51313	4.81132	0.17865
C	-2.33606	4.66066	0.12349
C	-0.23624	5.39654	-0.86757
C	-0.21746	4.31672	1.27864
C	-1.60446	4.22805	1.23956
C	-1.63034	5.32641	-0.8904
H	0.27126	5.86375	-1.69205
H	0.29675	3.87413	2.1101
H	-2.10914	3.71543	2.03769
H	-2.16014	5.72784	-1.73655
C	5.43345	1.99359	-0.22396
C	6.65873	-0.67677	-0.1481
C	6.15738	1.53511	0.93822
C	5.67769	1.25277	-1.43731
C	6.29602	0.04022	-1.43156

C	6.78048	0.32244	0.97414
H	6.2216	2.16846	1.80362
H	5.34573	1.64637	-2.37874
H	6.44571	-0.48653	-2.35764
H	7.31742	0.02339	1.85834
C	-3.71217	4.08217	-0.06381
C	-5.62715	1.98283	-0.1189
C	-3.97508	3.37546	-1.24727
C	-4.5978	3.89077	1.01086
C	-5.53531	2.85843	0.98781
C	-4.92923	2.3618	-1.28541
H	-3.33929	3.51539	-2.10225
H	-4.48671	4.47741	1.90591
H	-6.12868	2.67375	1.8656
H	-4.99565	1.75541	-2.16899
C	-6.04048	0.56973	0.0163
C	-5.51254	-2.22243	-0.01019
C	-6.40188	-0.23069	-1.09237
C	-5.68343	-0.11357	1.19813
C	-5.40152	-1.47664	1.17386
C	-6.14833	-1.60228	-1.10067
H	-6.78747	0.23307	-1.98285
H	-5.4442	0.44112	2.08578
H	-4.95207	-1.92083	2.04253
H	-6.34119	-2.16353	-1.99807
C	-4.5918	-3.40324	-0.18033
C	-2.08451	-4.76038	-0.15943
C	-3.75411	-3.40328	-1.30637
C	-4.25173	-4.27073	0.87082
C	-3.02207	-4.93097	0.88549
C	-2.54039	-4.08201	-1.30904
H	-3.98405	-2.76613	-2.13971
H	-4.90346	-4.36279	1.72264
H	-2.76357	-5.52371	1.74412
H	-1.88614	-3.94693	-2.14905
C	-0.63651	-4.97385	0.01394
C	2.1462	-4.34332	0.25832
C	0.24372	-5.07549	-1.09138
C	-0.0372	-4.7716	1.28005
C	1.31342	-4.46102	1.3966
C	1.59632	-4.77051	-0.97321
H	-0.1452	-5.32154	-2.06266
H	-0.64806	-4.73942	2.16249
H	1.70968	-4.24185	2.37103
H	2.20001	-4.75931	-1.8622

C	3.4194	-3.60426	0.3101
C	5.60953	-1.77234	0.13295
C	4.46559	-3.79757	-0.61912
C	3.56011	-2.52622	1.2083
C	4.62148	-1.62853	1.1143
C	5.53514	-2.90624	-0.69686
H	4.43633	-4.63542	-1.29248
H	2.7891	-2.32841	1.92852
H	4.64026	-0.78599	1.77768
H	6.29958	-3.07647	-1.43609
H	7.61715	-1.1753	-0.28439

Zero-point correction= 0.655984 (Hartree/Particle)
Thermal correction to Energy= 0.691238
Thermal correction to Enthalpy= 0.692182
Thermal correction to Gibbs Free Energy= 0.588842
Sum of electronic and zero-point Energies= -1848.253981
Sum of electronic and thermal Energies= -1848.218727
Sum of electronic and thermal Enthalpies= -1848.217783
Sum of electronic and thermal Free Energies= -1848.321123

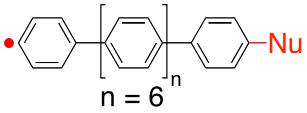

26

Nu = H

Charge = 0 Multiplicity = 2

H	-0.60931	-2.05833	5.36156
C	-0.31868	-1.16064	5.87461
C	0.31793	1.16053	7.26537
C	0.	0.	5.13405
C	-0.31793	-1.16053	7.26537
C	0.	0.	8.00401
C	0.31868	1.16064	5.87461
H	-0.60678	-2.05789	7.78056
H	0.60931	2.05833	5.36156
H	0.60678	2.05789	7.78056
C	0.	0.	3.66512
C	0.	0.	0.79293
C	0.25535	-1.17626	2.92408
C	-0.25535	1.17626	2.92408
C	-0.25436	1.17645	1.53365
C	0.25436	-1.17645	1.53365

H	0.49737	-2.08853	3.43666
H	-0.49737	2.08853	3.43666
H	-0.49518	2.08871	1.02039
H	0.49518	-2.08871	1.02039
C	0.	0.	-0.6756
C	0.	0.	-3.54775
C	-0.33713	-1.15556	-1.41626
C	0.33713	1.15556	-1.41626
C	0.3377	1.15536	-2.80667
C	-0.3377	-1.15536	-2.80667
H	-0.64219	-2.04842	-0.90293
H	0.64219	2.04842	-0.90293
H	0.64364	2.04813	-3.31936
H	-0.64364	-2.04813	-3.31936
C	0.	0.	-5.01621
C	0.	0.	-7.88879
C	0.23772	-1.17993	-5.7573
C	-0.23772	1.17993	-5.7573
C	-0.23801	1.17983	-7.14772
C	0.23801	-1.17983	-7.14772
H	0.46597	-2.09563	-5.24449
H	-0.46597	2.09563	-5.24449
H	-0.46587	2.09558	-7.66065
H	0.46587	-2.09558	-7.66065
C	0.	0.	9.47667
C	0.	0.	12.32438
C	-0.31831	1.16061	10.21055
C	0.31831	-1.16061	10.21055
C	0.31982	-1.1581	11.60363
C	-0.31982	1.1581	11.60363
H	-0.59848	2.05966	9.69359
H	0.59848	-2.05966	9.69359
H	0.58452	-2.05551	12.13407
H	-0.58452	2.05551	12.13407
C	0.	0.	13.82198
C	0.	0.	16.58004
C	0.99934	0.68788	14.53057
C	-0.99934	-0.68788	14.53057
C	-1.00924	-0.6953	15.93206
C	1.00924	0.6953	15.93206
H	1.76773	1.20876	13.9874
H	-1.76773	-1.20876	13.9874
H	-1.77402	-1.21953	16.47552
H	1.77402	1.21953	16.47552
C	0.	0.	-9.35768

C	0.	0.	-12.22742
C	-0.33862	-1.15508	-10.09814
C	0.33862	1.15508	-10.09814
C	0.3378	1.15494	-11.4889
C	-0.3378	-1.15494	-11.4889
H	-0.64545	-2.04752	-9.58532
H	0.64545	2.04752	-9.58532
H	0.64234	2.04693	-12.00445
H	-0.64234	-2.04693	-12.00445
C	0.	0.	-13.70021
C	0.	0.	-16.53054
C	0.31956	-1.16405	-14.43089
C	-0.31956	1.16405	-14.43089
C	-0.32106	1.16272	-15.82483
C	0.32106	-1.16272	-15.82483
H	0.59743	-2.061	-13.90892
H	-0.59743	2.061	-13.90892
H	-0.57997	2.06107	-16.35634
H	0.57997	-2.06107	-16.35634
H	0.	0.	-17.60566

Zero-point correction= 0.655298 (Hartree/Particle)
Thermal correction to Energy= 0.692392
Thermal correction to Enthalpy= 0.693336
Thermal correction to Gibbs Free Energy= 0.580013
Sum of electronic and zero-point Energies= -1848.309135
Sum of electronic and thermal Energies= -1848.272041
Sum of electronic and thermal Enthalpies= -1848.271097
Sum of electronic and thermal Free Energies= -1848.384420

X-ray Crystallography. Diffraction intensities for **Juasti22** were collected at 173(2) on a Bruker Apex2 CCD diffractometer using a micro-focus Incoatec IμS CuKα source, λ= 1.54178 Å. Space group were determined based on systematic absences. Absorption correction was applied by SADABS[8]. Structure was solved by direct methods and Fourier techniques and refined on F^2 using full matrix least-squares procedures. All non-H atoms were refined with anisotropic thermal parameters. H atoms in the main molecule were refined in calculated positions in a rigid group model. The molecule has a C*m* symmetry. A solvent molecule CH_2Cl_2 is located on a mirror plane and disordered over six possible positions (two positions for the carbon atom and three positions for Cl atoms); two C positions and one Cl position are on a mirror plane and two other Cl positions are related by the mirror plane. Refinement shown that Cl atoms of the solvent molecule is disordered over there mentioned positions with the same occupation factor (0.667) and occupation factors for two carbon positions are slightly different (0.37 vs. 0.13). All calculations were performed by the Bruker SHELXTL package[9].

Crystallographic Data for Juasti22: $C_{49}H_{34}Cl_2$, ($C_{48}H_{32}$•CH_2Cl_2), M = 693.66, 0.23, 0.14 x 0.11 x 0.03 mm, T = 173(2) K, orthorhombic, space group *Abm*2, a = 13..3249(9) Å, b = 26.5982(15) Å, c = 10.0862(6) Å, V = 3574.7(4) Å^3, Z = 4, D_c = 1.289 Mg/m^3, μ = 1.892 mm^{-1}, F(000) = 1448, $2\theta_{max}$ = 133.34°, 10794 reflections, 2516 independent reflections [R_{int} = 0.0349], R1 = 0.0750, wR2 = 0.2148 and GOF = 1.073 for 2516

---

[8] G. M. Sheldrick, *Bruker/Siemens Area Detector Absorption Correction Program*, Bruker AXS, Madison, WI, 1998.

[9] Sheldrick, G. M. (2008). *Acta Cryst*. A64, 112-122.

reflections (239 parameters) with I>2σ(I), R1 = 0.0809, wR2 = 0.2231 and GOF = 1.068 for all reflections, max/min residual electron density +0.973/-0.342 eÅ^3.

Calculation of Theoretical Yield of Nanotubes from Growth

There are 4.22 CPPs along the length of 1 nm of an armchair CNT and 1,000,000 nm in 1 mm. Multiplying 1 mg by 4,220,000 (4.22 X 1,000,000) gives approximately 4 kg.

There are 989 X 10^{15} molecules of [8]CPP in 1 mg, giving the potential to produce 989 X 10^{15} nanotubes from 1 mg of template.

HOMO Orbital Coefficients of Fully Aryl-Substituted 27.

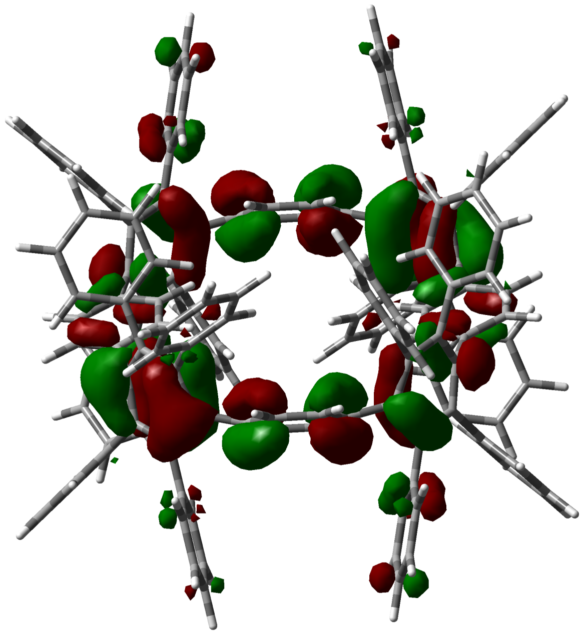

Supplement: Supplementary file 1 [file SC-007-C5SC04218F-s001.pdf]
